# Subphthalocyanine–triangulene dyads: Property tuning for light‐harvesting device applications

**DOI:** 10.1002/ese3.1071

**Published:** 2022-01-17

**Authors:** Mads Georg Rasmussen, Malte Frydenlund Jespersen, Olivier Blacque, Kurt V. Mikkelsen, Michal Juríček, Mogens Brøndsted Nielsen

**Affiliations:** ^1^ Department of Chemistry University of Copenhagen Copenhagen Denmark; ^2^ Department of Chemistry University of Zurich Zurich Switzerland

**Keywords:** chromophores, conjugation, molecular engineering, redox‐active molecules, structure–property relationships

## Abstract

Organic photovoltaics relies on the development of stable chromophores and redox‐active organic molecules with tailor‐made HOMO/LUMO energies. Here, we present the synthesis and properties of novel dyads composed of boron subphthalocyanine (SubPc) and triangulene units, connected either at the peripheral position of the subphthalocyanine or at the axial boron. The connectivity has strong implications for the absorption and fluorescence properties of the dyads, as well as their redox properties. While the SubPc unit has a bowl shape, triangulene is a planar structural unit that allows dyads to dimerize in the solid state on account of π‐stacking interactions as shown by X‐ray crystallography of one of the dyads. The electronic properties were also studied computationally by density functional theory methods. Excellent agreement between experimental and computed data were obtained, showing that our computational method is a strong tool in the rational design of optimum molecules to ultimately obtain finely tuned molecules for device applications.

## Graphical abstract

Novel subphthalocyanine–triangulene dyads were synthesized and investigated both experimentally and computationally for their optical and redox properties; these properties were found to depend strongly on the connectivity and spatial orientations of the two units. Experimentally determined redox potentials for the molecules were found to match excellently with calculated potentials, showing the strength of our computational method for tailoring molecules with specific electronic properties.

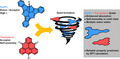

## 1 INTRODUCTION

**Figure 1:**
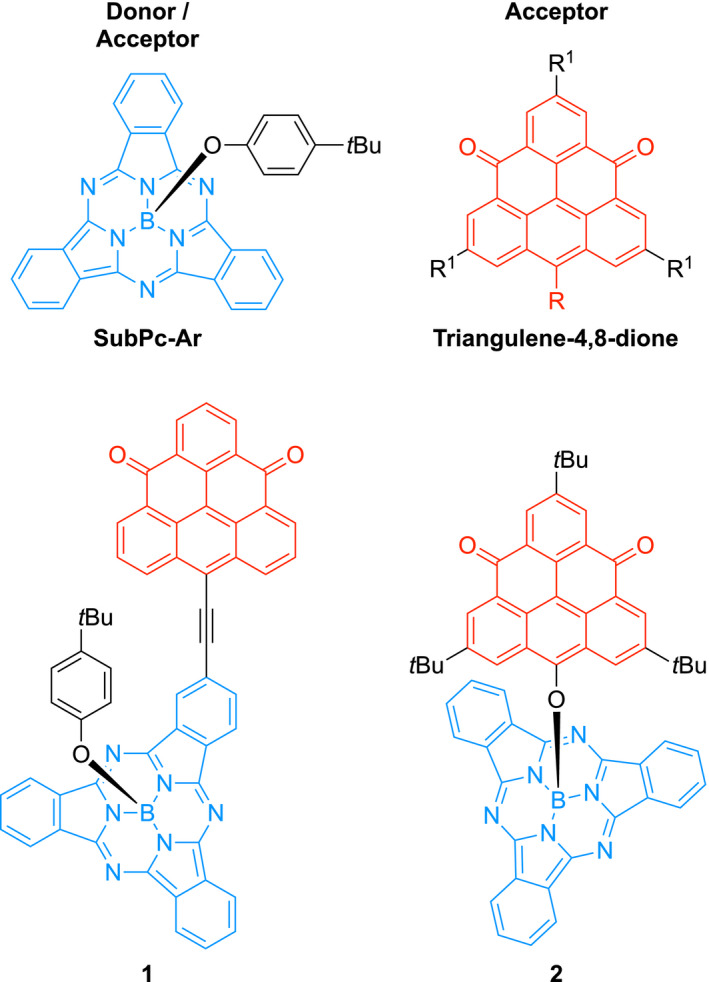
**SubPc‐Ar** (blue) and **triangulene‐4,8‐dione** (red) and target dyad systems **1** and **2**

Boron subphthalocyanine (SubPc) is a concavo‐convex 14‐π aromatic chromophore consisting of three aza‐linked isoindole units with an sp^3^‐hybridized boron metalloid in the center at which an axial substituent is placed.^1^ Via peripheral and axial substitutions, SubPc has tunable reduction and oxidation potentials as well as optical properties.^2^, ^3^, ^4^ For example, the structure **SubPc‐Ar** shown in Figure 1 is characterized by an intense absorption in the visible region (563 nm, ε ≈ 90,000 M^–1^ cm^–1^ in toluene),^5^ naturally sparking an interest for application of SubPc derivatives in organic light‐emitting diodes (OLEDs) and organic photovoltaic devices (OPVs).^2^, ^3^, ^4^, ^6^, ^7^, ^8^, ^9^, ^10^, ^11^ Additionally, the bowl shape enables axial modification and provides a convenient way for introduction of solubility‐enhancing substituents such as 4‐*tert*‐butylphenolate as shown in substrate **SubPc‐Ar**. This geometry also conveniently prevents strong associations between molecules.

As demonstrated in the work of Takui^12^, ^13^ and Morita,^14^, ^15^ trioxo‐triangulene and like derivatives have proven as excellent electron acceptors with very high thermal stability in conducting materials. Strong intermolecular interactions of the triangulene scaffold, as a result of mixing of the singly occupied molecular orbitals (SOMOs),^16^ provide self‐assembly ability of the triangular‐shaped acceptor. As recently shown by Juríček et al.,^17^, ^18^, ^19^ **triangulene‐4,8‐dione** molecular motifs synthetically modified for further functionalization (Figure 1) can be obtained via robust synthetic routes on gram scale. In addition to exhibiting significantly higher solubility, these triangulene‐4,8‐diones are visible light/near infrared emitters with large Stokes shifts and high quantum yields, while maintaining inherent triangulene redox properties.^17^

The light‐harvesting and donor‐and‐acceptor capabilities of SubPc paired with the acceptor and photophysical properties of the triangulene‐4,8‐dione scaffold make these two molecules powerful molecular building blocks for the formation of tunable donor–acceptor dyad systems or novel non‐fullerene acceptors with acceptor properties altered relative to the individual units as a consequence of being linked together. Indeed, calculations predict SubPc derivatives to be efficient donor molecules in photovoltaics devices,^11^, ^20^, ^21^and, for example, SubPc derivatives with bithiophene or quaterthiophene as axial ligands were experimentally used as donors together with C_60_ as the acceptor.^11^ The different geometrical shapes of the SubPc and triangulene units, bowl shape and planar, respectively, could potentially also be important in regard to tuning the materials morphology, a particularly important factor to take into account for optimum performance of an organic solar cell.^22^

In this work, we present the syntheses and properties of two SubPc–triangulene‐4,8‐dione dyads **1** and **2** in addition to four new triangulene‐4,8‐dione derivatives. Supported by density functional theory (DFT) computational studies, we elucidate on redox and photophysical property‐tuning of the triangulene‐4,8‐dione and SubPc chromophores. Indeed, being able to tailor donor and acceptor HOMO and LUMO energy levels as well as spectral window for light absorption with precision is crucial for a rational design of organic materials.^23^, ^24^, ^25^ For example, the open circuit voltage for a donor–acceptor system, corresponding to the maximum voltage that can be drawn from a photovoltaic device, relates to the HOMO and LUMO energies of the donor and acceptor.^26^, ^27^ One major objective of this work is to benchmark computations with experiments in order to reliably predict fundamental electronic properties of constituents alone and when linked together.

## 2 METHODS

See Supporting information.

## 3 RESULTS AND DISCUSSION

**Scheme 1:**
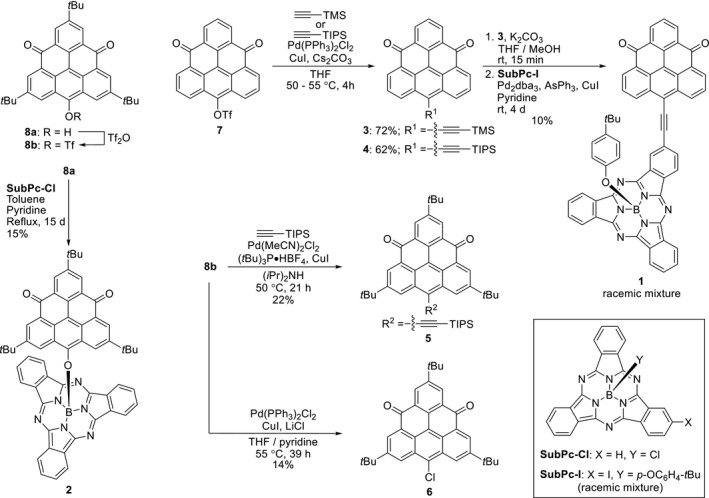
Synthesis of dyads **1** and **2**

Synthesis of the dyads **1** and **2** in addition to the triangulene‐4,8‐dione derivatives **3**, **4**, **5**, and **6** proceeded as depicted in Scheme 1. From the known triflate **7**,^18^ compounds **3** and **4** could conveniently be obtained by Sonogashira coupling reactions in acceptable yields within short reaction times of 4 h. For the in‐situ deprotonation step of the added silyl‐protected alkyne, the use of Cs_2_CO_3_ as an alternative to amine base derivatives proved critical; this was to suppress an otherwise competing decomposition of the starting material **7**. Desilylation of **3** could be obtained under mild conditions; however, the corresponding deprotected, terminal alkyne (**3a**) exhibited extremely poor solubility. Nevertheless, by using neat pyridine as a solvent, sufficient solubility was provided to facilitate a successful Sonogashira coupling with the known **SubPc‐I** ^28^ to give **1** as a racemic mixture in 10% yield over 4 days. For the reaction, a catalyst system of Pd_2_dba_3_/AsPh_3_/CuI was employed as this has previously proved very reliable for Pd‐catalyzed couplings of iodo‐SubPcs.^5^, ^10^, ^29^, ^30^ Formation of dyad **2** was initiated from trioxo‐triangulene **8a** ^17^ and **SubPc‐Cl** ^31^ that were both synthesized as described in the literature. Increasing the temperature of the reaction of **8a** and **SubPc‐Cl** to reflux in a solvent mixture of pyridine/toluene proved necessary to afford dyad formation. After 15 days of reflux, the axially coupled dyad **2** could successfully be obtained in a 15% yield after chromatographic purification. Via the known triflate **8b**,^17^ the triangulene derivatives **5** and **6** were obtained. Initially, for the formation of **5**, reaction conditions analogous to those for the formation of **3** and **4** were applied. The triflate **8b**, however, proved to be unstable under these conditions. Successful Sonogashira couplings could nonetheless be achieved by employing an alternative catalyst system of Pd(MeCN)_2_Cl/(*t*Bu)_3_P•HPF_4_/CuI in neat diisopropylamine, which has been shown to perform well for coupling of sterically hindered aryl substrates, as shown by Márquez et al.^32^

X‐Ray crystallographic analysis of **5** reveals some distortion of the planarity of the triangulene core. In addition, the silyl‐group is bent significantly out of plane (1.33 Å; see Figure S70) as a result of the limited space provided by the neighboring *t*Bu groups. These observations reflect well with unfavorable formation of **5** and the low isolated yield of 22%. Triflate **8b** was shown to be able to form the chlorinated triangulene **6** under the applied coupling conditions when chloride ions were present. As similarly observed by Stille and Echavarren for electron deficient aryl triflates, this formation could be promoted by addition of LiCl to the reaction mixture, suggesting formation of **6** might proceed via a reductive elimination pathway.^33^

Compounds **1**–**6** were all characterized by ^1^H/^13^C NMR spectroscopies and high‐resolution mass spectrometry (HRMS; ESI/APCI; Figures S1–S37). Dyad **1** and its triangulene precursor **3** were further analyzed by 2D NMR spectroscopic methods (COSY, HSQC and HMBC) to provide unambiguous structural confirmation of **1** (Figure S18). Additionally, dyad **2** and triangulenes **4**, **5**, and **6** were analyzed by single‐crystal X‐ray diffraction (Figure 2; Figures S61–S78). In the crystal packing of all four substrates, a head‐to‐head packing of the triangulene moiety is observed with intermolecular distances of 3.4–3.5 Å.

**Figure 2:**
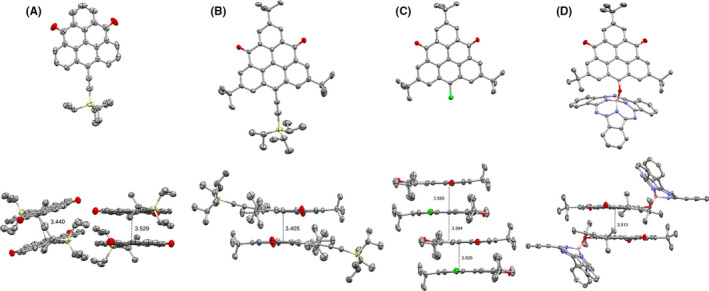
X‐Ray crystallographic perspective view of solid‐state structures (top) and side view of triangulene solid‐state packing with distances in Å (bottom): a) **4** (CCDC 2113702), b) **5** (CCDC 2113700), c) **6** (CCDC 2113699), and d) **2** (CCDC 2113701). Crystals of **4** were grown from CH_2_Cl_2_/hexane bilayer system; crystals of **2**, **5**, and **6** were grown from CH_2_Cl_2_/MeOH bilayer system. Thermal ellipsoids are displayed at a 50% probability level. Hydrogen atoms are omitted for clarity. CH_2_Cl_2_ solvent molecules are omitted for **4**

The alkyne‐extended triangulene derivative **4** was shown to crystalize in two alternating π‐stacking columns with staggered head‐to‐head packing and different packing distances of 3.4 and 3.5 Å. For the analog but more sterically strained *t*Bu triangulene **5**, a slipped π‐stacking in separate dimer pairs with a monoclinic crystal system is observed. For the chlorinated triangulene **6**, crystal packing exhibits staircase‐like motif of staggered head‐to‐head π‐dimers (3.5 Å distance), with a 3.4 Å distance between stacked pairs. The dyad **2** proved to crystallize in a triclinic crystal system with slipped π‐dimer packing motif in respect to triangulene with a packing distance of 3.5 Å. A slipped packing of the SubPc bowl‐cavities of **2** facing one another is seen, with alternating boron–boron distances of 8.3 and 10.6 Å along the *a*‐axis. This packing orientation provides distinct three‐dimensional SubPc and triangulene domains alternating within the crystal (Figures S71–S78).

The photophysical properties of **1**–**6** were investigated by UV–vis and fluorescence spectroscopic methods, and the results are summarized in Table 1, and all spectra are shown in Figures S38–S56. The absorption profiles of the triangulene‐4,8‐dione structures **3**, **4**, **5**, and **6** measured in CH_2_Cl_2_ all exhibit characteristic triangulene absorption profiles consisting of two bands between 508–561 nm with a molar absorptivity (ε) in the range ≈ 9000–17,000 M^–1^ cm^–1^ and a sharp intense band in the UV region (277–283 nm). For the chlorinated triangulene **6**, a small hypsochromic shift of λ_max_ (~15 nm) is observed, when compared to the alkynyl‐silyl‐containing derivatives **3**, **4**, and **5**.

**Table 1:** Summarized data from photophysical, electrochemical and DFT computational studies

Compound	*λ*_Abs (nm)_	ε (10^4^ M^–1^ cm^–1^)	*λ*_calc_ (nm)	*f*_calc_	*λ*_PL_ (nm)	*Ф*_PL_	Solvent	*E*_ox_ (V)^a^	*E*_red_ (V)^a^	*E*_ox,calc_ (V)^b^	*E*_red, calc_ (V)^b^
**1**	283 311 558 581 621	5.98 3.92 4.82 5.36 6.25	–	–	645 701	0.41	Toluene	–	–	–	–
	278 310 558 582 616	9.38 4.22 4.92 5.01 5.77	315 340 493 500 558	0.16 0.21 0.28 0.40 1.29	–	–	CH_2_Cl_2_	0.54	–1.11 (–1.43)	0.61	–1.15
**2**	285 400 524 567	7.50 0.39 2.44 6.12	–	–	622 677	–^d^	Toluene	–	–	–	–
	279 403 513 567	9.01 0.49 2.27 6.12	391 477 507 512	0.04 0.40 0.44 0.40	640 683	–^d^	CH_2_Cl_2_	0.66 (0.95)	–1.49 (–1.60)	0.62	–1.62
**3**	277 405 523 558	4.89 0.32 1.05 0.88	–	–	590 636	0.67	CH_2_Cl_2_	1.14	–1.15 (–1.68)	–	–
**3a** ^e^	–	–	266 298 343 479	0.20 0.09 0.11 0.45	–	–	–	–	–	1.20	–1.13
**4**	277 404 525 561	7.83 0.45 1.72 1.49	268 298 343 364 490	0.21 0.07 0.10 0.06 0.58	592 640	0.68	CH_2_Cl_2_	1.15	–1.16 (–1.70)	1.12	–1.16
**5**	283 417 525 556	7.32 0.59 1.48 1.48	254 303 357 509	0.76 0.08 0.13 0.58	605 645	0.71	CH_2_Cl_2_	1.01	–1.27 (–1.78)		–1.21
**6**	282 410 508 540	7.97 0.58 1.21 1.10	251 270 351 364 480	1.69 0.19 0.12 0.04 0.36	588 628	0.62	CH_2_Cl_2_	1.09	–1.29 (–1.80)	1.08	–1.31
**8a**	–	–	252 269 397 501	1.09 0.74 0.08 0.30	–	–	CH_2_Cl_2_	–	–	0.77	–1.45

^a^ First and (second) oxidation and reduction potentials vs. Fc/Fc^+^ couple. Solvent: CH_2_Cl_2_; supporting electrolyte: 0.1 M Bu_4_NPF_6_; scan rate 0.1 V/s.

^b^ Calculated first reduction and oxidation potentials in CH_2_Cl_2_ using (CAM‐B3LYP/6‐31+g(d,p)); Obtained eV‐values have been referenced versus eV‐correlated Fc/Fc^+^‐couple value determined by Ree et al.^35^ to obtain approximate value in V (Supporting information, Equation 1).

^c^ No emission observed.

^d^ No emission observed within the limit of integration sphere and detector.

^e^ Compound with the TMS of **3** replaced by a hydrogen atom.

**Figure 3:**
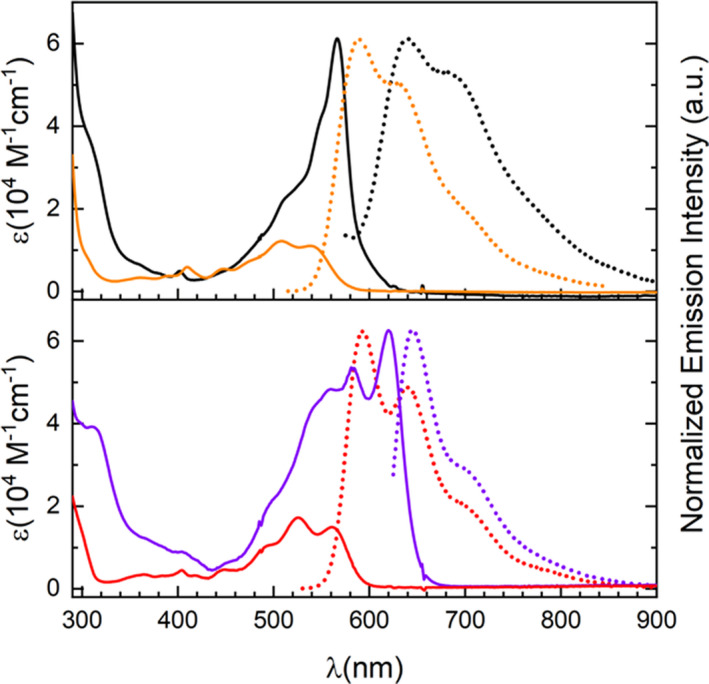
Stacked UV–vis (full) and normalized emission (dotted) spectra of: **2** (black; in CH_2_Cl_2_), **6** (orange; in CH_2_Cl_2_), **1** (purple; in toluene), and **4** (red; in CH_2_Cl_2_)

UV–vis spectroscopic analysis of the axially coupled dyad **2** measured in CH_2_Cl_2_ (Figure 3, black) revealed an absorption spectrum that resembles the superimposed absorption profiles for that of monomeric **SubPc‐Ar** and *t*Bu triangulene. A sharp Q‐band absorption (λ_max_ = 567 nm, ε ≈ 61,200 M^–1^ cm^–1^), characteristic of the SubPc S_0_–S_1_ transition can be observed, in addition to a lower intensity shoulder band (490–520 nm) corresponding well with triangulene‐4,8‐dione absorption. In contrast, dyad **1** with conjugated electronic connectivity between the SubPc and triangulene moiety displays a sharp and intense Q‐band absorption (Figure 3, purple; measured in toluene) at λ_max_ = 621 nm (ε ≈ 62,500 M^–1^ cm^–1^) with a bathochromic shift of 58 nm, when compared to that of monomeric **SubPc‐Ar**, which correlates nicely with calculated values of 51–60 nm (Tables S1–S2 and S5–S6). An enhancement of the molar absorptivity of **1** in the absorption window of ~500–570 nm is also observed. This becomes very evident when comparison with the absorption profile of the corresponding alkynyl‐extended triangulene fragment **4** is made (Figure 3, red). This increase occurs without a significant compromise of the absorption intensity for the red‐shifted Q‐band absorption of the SubPc core. TD‐DFT calculations reveal that this enhancement of absorptivity is caused partly by a small red‐ and blue‐shift of the transitions corresponding to the triangulene and SubPc unit, respectively (Tables S1–S2 and S5–S8). These combined effects lead to a better overlap between the two absorption peaks and hence enhanced absorption in this region. In the SubPc monomer, two electronic transitions separated by 1–2 nm, according to TD‐DFT calculations, are responsible for the strong absorption observed experimentally around 570 nm; these two transitions are separated by 56–66 nm in **1**, however, a significant increase in the oscillator strength is observed for the red‐shifted transition in **1**, which counteract the decrease expected by separating the two overlapping peaks. Additionally, **1** showed solvatochromic behavior. A color change visible by eye from deep purple (in toluene) to dark blue (in CH_2_Cl_2_) was observed. This is a result of subtle broadening of the absorption profile toward higher wavelengths, annotated to an increase in self‐aggregation of **1** in CH_2_Cl_2_.

For a summary of excitation energy calculations for the various compounds, see Tables S1–S16.

Substrates **2**–**6** proved fluorescent in CH_2_Cl_2_ at room temperature, whereas complete quenching of the emission for **1** was observed in CH_2_Cl_2_. However, in solvents counteracting π‐stacking such as toluene, **1** showed to be emissive (Figure S48).

**Figure 4:**
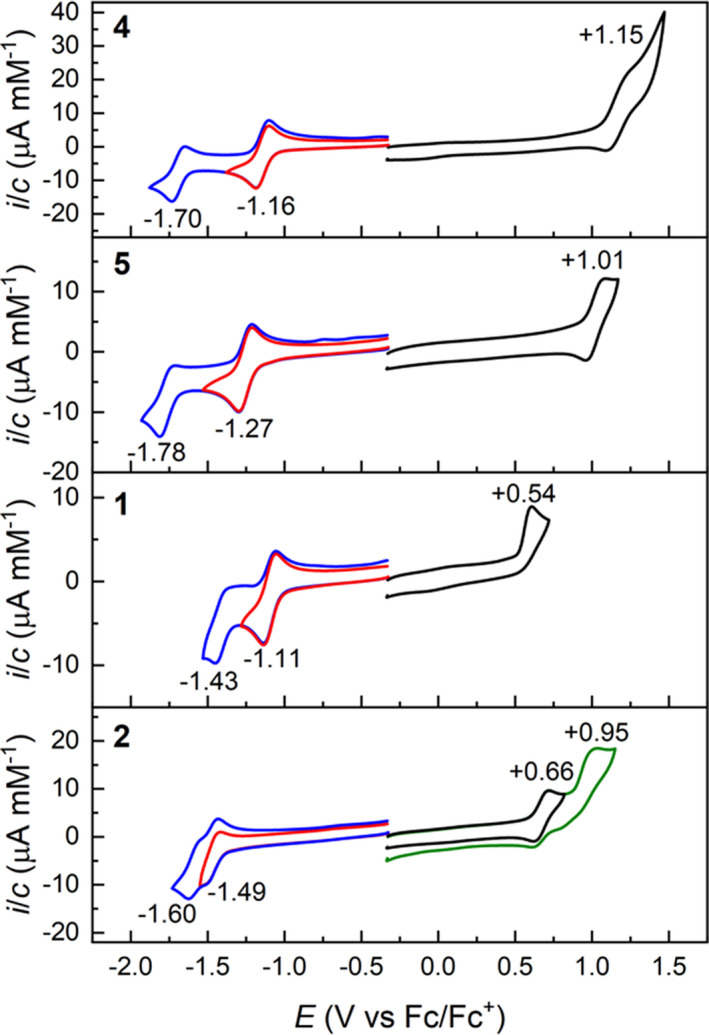
Stacked cyclic voltammograms (CV) of **1**, **2**, **4**, and **5** in CH_2_Cl_2_ with Bu_4_NPF_6_ (0.1 M) as supporting electrolyte; scan rate: 0.1 V/s; potentials vs Fc/Fc^+^. Listed redox potentials were determined from differential pulse voltammograms (DPV)

Triangulene derivatives **3** and **4** (Figures 3 and 4: red) both exhibit broad tailing emission profiles with 32‐ and 31‐nm Stokes shifts and photoluminescence quantum yields (QY) of 67% and 68%, respectively.

Comparable emission profiles were observed for the triangulene derivatives **5** and **6** (Figures 3 and 6: orange), with larger Stokes shifts (~49 nm), as a result of the introduction of electron‐donating *t*Bu groups. Compound **5** exhibited a QY of 71%, whereas a slightly lower QY (61%) was observed for the chlorinated substrate **6**. In toluene, dyad **1** proved emissive (Figure 3, purple) with a QY of 41%, moderately higher than monomeric **SubPc‐Ar** (30% in CHCl_3_).^34^ Excitation spectra of **1** recorded at 645 and 700 nm emission displayed clear indication that the broad emission profile of **1** with a 24‐nm Stokes shift stems from one component. For the electronically disconnected dyad **2**, a broad emission profile (Figure 3, black) was observed resembling that of the triangulene core with a high Stokes shift‐value of 73 nm in CH_2_Cl_2_ (53 nm in toluene), generally not observed for SubPc substrates. Excitation spectra of **2** recorded at both 620 and 680 nm confirms that emission stems from only one component, suggesting a possible energy‐transfer process from the SubPc core to the triangulene. The QY of **2**, however, proved to be very low, which can be attributed to possible non‐radiative lone‐pair quenching of the triangulene emission from the axially positioned oxygen‐bridge. Hence, no value for QY of **2** could be quantified, because no data of sufficient quality could be collected within the limit of the integration sphere and detector setup used for QY determination of the substrates **1** and **3**–**6**.

The redox properties of **1**–**6** were investigated by cyclic voltammetry (CV) and differential pulse voltammetry (DPV) in CH_2_Cl_2_ with ferrocene/ferrocenium (Fc/Fc^+^) couple as a reference (measured in a separate experiment; Table 1 and Figure 4; see also Figures S57–S60). Initially, an investigation of the oxidation potentials as a function of concentration for the simpler substrate **4** in CH_2_Cl_2_ was carried out (Figure S57). This study was performed because NMR spectroscopy had made it evident that self‐aggregation of all the substrates **1**–**6** occurred at ~1.0 mM concentration in CD_2_Cl_2_. From the experiment, it was concluded that no observable change in the oxidation potential of **4** could be seen within 0.10–0.52 mM concentration range. Hence, the CV and DPV experiments for **1**–**6** were carried out at ~0.5 mM concentrations. Similarly to previously reported analogous triangulene‐4,8‐dione substrates,^17^ compounds **3**, **4**, **5**, and **6** all showed one irreversible one‐electron oxidation wave and two one‐electron reduction waves, the first being reversible. The alkyne‐extended triangulene‐4,8‐dione derivatives **3** and **4** exhibited comparable electrochemical properties with very small potential difference for the first oxidation wave (*E*_ox_ = 1.14 and 1.15 V). Likewise, comparable potentials of *E*_red,1_ = –1.15 and –1.16 V for the reversible first reduction was observed, followed by an irreversible second reduction at *E*_red,2_ = –1.68 and –1.70 V, respectively.

For the *t*Bu‐functionalized derivative **5**, a shift toward more negative potential (*E*_red,1_ = –1.27 V) for the reversible first reduction is observed when compared to **4**. This is a result of introduction of the electron‐donating *t*Bu substituents. Similarly, the subsequent second irreversible reduction of **5** (*E*_red,2_ = –1.78 V) is moved toward slightly more negative potential. For the first oxidation wave of **5**, an expected lower oxidation potential (*E*_ox,1_ = 1.01 V) was observed, in line with increased donor strength of the triangulene core. An electrochemical reduction profile similar to that of **5** was seen for the chlorinated triangulene **6**, with a reversible first reduction at *E*_red,1_ = –1.29 V, followed by an irreversible second reduction at *E*_red,2_ = –1.80 V. In contrast, the first oxidation of **6** was more difficult, occurring at *E*_ox,1_ = 1.09 V. For dyad **1**, an irreversible one‐electron oxidation of *E*_ox,1_ = 0.54 V was measured, annotated to oxidation of the SubPc core, as the measured value is in good agreement with the previously determined oxidation potential of *E*_ox,1_ = 0.55 V for **SubPc‐Ar** in CH_2_Cl_2_.^34^ Upon reduction of **1**, a reversible one‐electron first reduction of *E*_red,1_ = –1.11 V was observed, attributed to the formation of the radical anionic species of the triangulene‐4,8‐dione. This interpretation is supported by the calculated LUMO of **1** and the HOMO of its radical anion (Figures S88 and S101), as both orbitals are primarily located on the triangulene core (but also including part of the conjugated SubPc unit). Moreover, the similar, computationally determined reduction potentials of the monomeric alkyne‐functionalized triangulene fragment **3a** (where the TMS group of **3** was replaced by a hydrogen atom) of *E*_calc,red,1_ = –1.13 V and of dyad **1** *E*_calc,red,1_ = –1.15 V (Table 1) support that the first reduction occurs on the triangulene core. These values were determined from the corresponding eV‐value obtained using a CAM‐B3LYP/6‐31+g(d,p) method, and a following correction versus theoretically estimated eV‐correlated Fc/Fc^+^‐value previously determined by Mikkelsen et al.^35^ A second irreversible reduction for formation of the anionic species of **1** at a potential of *E*_red,2_ = –1.43 V was observed, attributed to the reduction of the SubPc core. Interestingly, when compared to the potential of the first reduction of monomeric **SubPc‐Ar** at *E*_red,1_ = –1.56 V (in CH_2_Cl_2_),^34^ the second reduction of **1** occurs more readily, despite the introduction of Coulombic repulsion from the already formed radical anion species. These observations suggest highly localized charge distribution environments on the corresponding triangulene and SubPc moieties within dyad **1**, both upon oxidation and reduction. For the axially coupled dyad **2**, two consecutive one‐electron reduction waves are observed at *E*_red,1_ = –1.49 V and *E*_red,2_ = –1.60 V, attributed to the formation of the radical anionic species localized on the triangulene and SubPc core, respectively. Placing the negative charge of the monoanion of **2** mainly at the triangulene core is supported by the HOMO of this anion. Yet, the LUMO of **2** is actually mainly at the SubPc unit (Figures S96 and S102). As filled orbitals are better described than empty orbitals within DFT, formation of a negatively charged triangulene unit after the first reduction seems most reliable (based on HOMO of anion). As the corresponding trioxo‐triangulene molecular fragment **8a** suffers from limited solubility (previously investigated as anionic ammonium complex by Takui et al.^12^, ^13^), no experimental values for comparison of redox potentials could be obtained in CH_2_Cl_2_. First oxidation and reduction potential values were, however, estimated by DFT calculations (CAM‐B3LYP/6‐31+g(d,p)) for the trioxo‐triangulene fragment **8a** in CH_2_Cl_2_ with values of *E*_ox,calc1_ = 0.77 V and *E*_red,calc1_ = –1.45 V. This reflects well the experimentally determined *E*_red,1_ = –1.49 V value of **2**, which again supports a first reduction mostly located on the triangulene core. Compound **2** has a significantly higher first reduction potential compared to that of compounds **1** and **3**–**6**. This is annotated to the introduction of an additional donating heteroatom on the triangulene core. For the oxidation of **2**, two one‐electron waves at *E*_ox,1_ = 0.66 V (oxidation of the SubPc core, corresponding to location of HOMO on **2** (Figure S97)) and *E*_ox,2_ = 0.95 V (attributed to subsequent oxidation of the trioxo‐triangulene core) are observed. The value for the second oxidation is in good correlation with the value obtained by DFT calculations for **8a** (*E*_ox,calc1_ = 0.77 V) when Coulombic repulsion from the already closely residing radical cationic charge on the SubPc (3.4 Å from the triangulene core; Figure S78) is taken into account. For an overview of calculated frontier orbitals of **1** and **2**, See Figures S85–S102.

**Figure 5:**
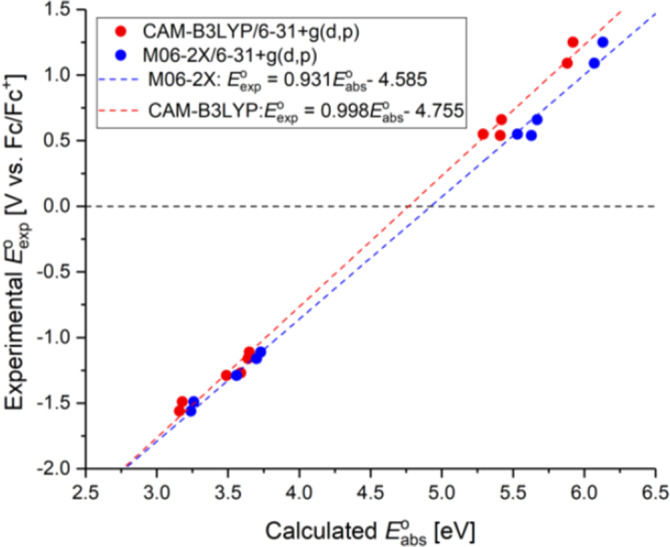
Linear correlation plot (LCP) of experimental and calculated standard redox potentials using different functionals with diffuse methods

Validation of our computational method used for determination of redox potentials was supported by a linear correlation plot (LCP) of experimental (*E*_exp_) and calculated absolute (*E*_abs_) redox potentials (Figure 5; Figure S110). A nearly perfect correlation between experimental and calculated redox potentials using CAM‐B3LYP/6‐31+g(d,p) is observed, validating the good performance of this method previously observed in the work of Ree et al.^35^ The LCP of M06‐2X/6‐31+G(d,p) shows much worse correlation, which can be tracked back as a trend to overestimate the oxidation potential; M06‐2X/6‐31+G(d,p) should not be disregarded as a method as its predictions and hence errors are systematic. Omitting diffuse functions (6‐31G(d,p)) significantly deteriorates the correlation observed for the CAM‐B3LYP and the M06‐2X methods (Figure S109). For lists of calculated redox potentials in comparison to experimental ones, see Tables S17 and S18.

For a summary of computed HOMO and LUMO energies, see Tables S17 and S18. Mulliken charge distributions of **1** and **2** are given in Figures S103–S108.

Internal reorganization energies of **1** and **2** are given in the Table S19. It appears that both hole and electron reorganization energies are higher for **2** than for **1**, which will lead to more efficient electron transfer in **1**. The calculated reorganization energies are in a similar range as recently calculated by Abbas et al.^36^ for iodo‐substituted SubPc derivatives with different axial groups.

**Figure 6:**
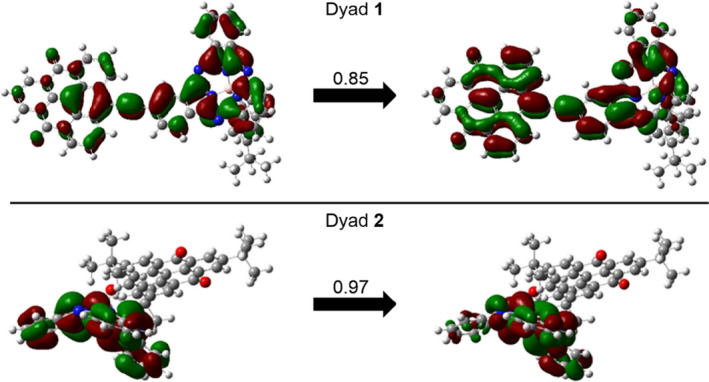
Natural transition orbitals (NTOs) for first excitation of **1** (top) and **2** (bottom) calculated with M06‐2X/6‐31g(d,p) with coefficients of transition

Natural transition orbitals (NTO) were calculated to visualize which orbitals are involved in the electronic transitions. Figure 6 shows the NTO for the lowest energy transition in **1** and **2**. The NTO clearly shows that the red‐shift observed in **1** is caused by the extension of the conjugated π‐systems, which enables the transition to occur over the entire π‐system, whereas for the disconnected π‐system in **2** the lowest electronic transition reassembles that of an isolated **SubPc‐Ar** monomer. In Supporting information, the second and third NTO of **1** and **2** are shown (Figures S79–S84). The position and nature of the three lowest transitions in **1** and **2** are responsible for the change in optical properties observed in the visible region and could be a possible target for further optimization to enhance the systems performance in future photovoltaic applications.

## 4 CONCLUSIONS

In conclusion, we have presented two new SubPc–triangulene‐4,8‐dione dyad systems **1** and **2**, illustrating the impact of structural design. This is demonstrated in the observed changes of inherent photophysical and redox properties, as a consequence of the differences in electronic connectivity and spatial orientation. Furthermore, we have presented the new triangulene‐4,8‐dione derivatives **3**, **4**, **5**, and **6** with discrete structural changes allowing to elucidate on tuning of physical properties of the triangulene core. X‐Ray crystallographic analyses of **2**, **4**, **5**, and **6** confirm the power of triangulene self‐assembly as a tool for morphologic control in the solid state, especially underlined by the self‐assembled formation of alternating donor and acceptor domains within the crystal packing of **2**.

While the axially connected dyad **2** had a longest‐wavelength absorption maximum similar to that of a SubPc monomer, the peripherally connected dyad **1** displayed a significantly red‐shifted longest‐wavelength absorption on account of the extended π‐conjugation, in agreement with a natural orbital analysis of the first excitation. The extended conjugation had, however, little influence on the first oxidation potential of **1**, which was close to that of a SubPc monomer, and the first reduction potential was close to that of a related triangulene monomer. Experimentally determined redox potentials for the entire series of molecules were found to match well with calculated potentials using the CAM‐B3LYP/6‐31+g(d,p) functional. This benchmarking study implies that fine‐tuning of electronic properties of SubPc–triangulene dyads for future device applications can be reliably predicted computationally.

## CONFLICT OF INTEREST

The authors declare no conflict of interest.

## Supporting information

Supplementary Material
