# Supplementary material for: Subphthalocyanine–triangulene dyads: Property tuning for light‐harvesting device applications
Source: Energy Sci Eng. 2022 Jan 17;10(5):1752–62. doi: 10.1002/ese3.1071 (PMC9306930; doi:10.1002/ese3.1071)
Supplement: Supplementary file 1 — Supplementary Material [file ESE3-10-1752-s001.docx]

**Subphthalocyanine-Triangulene Dyads: Property tuning for light-harvesting device applications**

**Supporting information**

Mads Georg Rasmussen^1^, Malte Frydenlund Jespersen^1^, Olivier Blacque^2^, Kurt V. Mikkelsen^1^, Michal Juríček^2^, Mogens Brøndsted Nielsen^1^

^1^Department of Chemistry, University of Copenhagen, Denmark

^2^Department of Chemistry, University of Zurich, Switzerland

**Correspondence:**
Mogens Brøndsted Nielsen, Department of Chemistry, University of Copenhagen, Universitetsparken 5, 2100 Copenhagen Ø. E-mail: mbn@chem.ku.dk

Kurt Valentin Mikkelsen, Department of Chemistry, University of Copenhagen, Universitetsparken 5, 2100 Copenhagen Ø. E-mail: kmi@chem.ku.dk

Michal Juríček, Department of Chemistry, University of Zurich, Winterthurerstrasse 190, CH-8057 Zurich. E-mail: michal.juricek@chem.uzh.ch

Table of contents

[Synthesis S2](#_Toc84612845)

[NMR spectra S7](#_Toc84612846)

HRMS of dyads **1** and **2** ………………………………………………………………………. S26

[UV-vis and fluorescence data S27](#_Toc84612847)

[Electrochemistry S37](#_Toc84612848)

[X-Ray crystallographic data S40](#_Toc84612849)

[Computational studies S57](#_Toc84612850)

[References S120](#_Toc84612851)

Synthesis

General methods

Compounds **SubPc-Cl**^[1]^, **SubPc-I**^[2]^, **7**^[3]^, **8a**^[4]^ and **8b**^[4]^ were all synthesized according to literature produdures. Chemicals and solvents have been purchased from commercial suppliers and used as received, unless otherwise stated. For thin layer chromatography (TLC) aluminium sheets precoated with silica gel and fluorescence indicator were used; visualized under UV light (254 or 360 nm). Flash column chromatography was carried out using SiO_2_ with particle size of 40 – 63 μm. ^1^H- and ^13^C NMR spectra were recorded on a Bruker 600 MHz instrument with an inverse triple-resonance cryo-probe or on a Bruker 500 MHz instrument with non-inverse cryo-prope. As reference, the deuterated solvents were used: CD_2_Cl_2_ (­^1^H NMR: δ = 5.32 ppm, ^13^C NMR: *δ* = 54.00 ppm) and benzene-*d_6_* _­_(­^1^H NMR: δ = 7.16 ppm, ^13^C NMR: *δ* = 128.06 ppm). HRMS data were collected with a **high-resolution Orbitrap HPLC-MS** instrument fitted with an electrospray **(ESI**) or atmospheric pressure (APCI) ion source. Melting points were measured on a temperature-controlled microscope and are uncorrected. UV-vis spectra were measured in 1-cm path-length cuvettes. Fluorescence measurements was carried out using an Edinburgh Instrument FS5 Spectrofluorometer with a SC-25 temperature-controlled holder TE-Cooled-Standard cell for emission spectra and a SC-30 Integrating Sphere cell for obtaining quantum yields. All solvents used for spectrophotometric analysis were of analytical grade. Electrochemical data were collected on an Autolab PGSTAT12 instrument setup using NOVA 1.11 software; all measurements were carried at rt in a CH_2_Cl_2_ solution with Bu_4_NPF_6_ (0.1 M) as supporting electrolyte which had been argon purged with a CH_2_Cl_2_ saturated argon stream prior to measuring. As working electrode, a glassy carbon disk (*d* = 3 mm) was used, as counter electrode a platinum wire and as reference electrode a silver wire separated from the substrate solution by a ceramic frit. The measured potentials were referenced against ferrocene/ferrocenium (Fc/Fc^+^) couple (1.0 mM), which was measured in separate experiments before and after the experiment.

**SubPc-Triangulene dyad (1)**

A flask was charged with a reaction mixture of **3** (41.5 mg, 103 µmol) and K_2_CO_3_ (43 mg, 311 µmol) in 5:1 THF/MeOH (30 mL) and stirred for 15 min. The formed dark precipitate (containing the terminal alkyne; **3a**) was isolated by filtration (pore size 4), washed with H_2_O (5 mL), then MeOH (10 mL), dried *in vacuo* and transferred to an argon-purged flask containing Pd_2_dba_3_ (10.4 mg, 11.3 µmol), AsPh_3_ (27.6 mg, 87.5 µmol), CuI (1.9 mg, 10 µmol) and **SubPc-I** (90 mg, 134 µmol). Argon degassed anhydrous pyridine (25 mL) was canulated into the sealed flask and the resulting reaction mixture was stirred at rt for 4 d, before being concentrated *in vacuo*. Purification by repeated flash column chromatography (SiO_2_, 10 – 15% EtOAc/toluene) followed by trituration with MeOH (3 x 4 mL), yielded the title compound **1** (9.1 mg, 10%) as a dark purple solid.

R*_f_*  = 0.26 (10% EtOAc/toluene) M.p.: >300 °C. ^1^H NMR (600 MHz, Benzene-*d_6_*): *δ* = 9.36 (dd, *J* = 1.4, 0.8 Hz, 1H), 8.97 (dd, *J* = 6.9, 1.2 Hz, 2H), 8.95 (dd, *J* = 8.4, 1.2 Hz, 2H), 8.83 (dd, *J* = 8.1, 0.8 Hz, 1H), 8.82 (d, *J* = 7.6 Hz, 2H), 8.80 – 8.77 (m, 1H), 8.77 – 8.75 (m, 1H), 8.75 – 8.70 (m, 2H), 7.83 (dd, *J* = 8.1, 1.4 Hz, 1H), 7.44 (dd, *J* = 8.4, 6.9 Hz, 2H), 7.41 – 7.37 (m, 2H), 7.37 – 7.34 (m, 2H), 7.18 (t, 7.6 Hz, 1H), 6.72 (d, *J* = 8.7 Hz, 2H), 5.70 (d, *J* = 8.7 Hz, 2H), 0.96 (s, 9H) ppm. ^13^C NMR (151 MHz, Benzene-*d_6_*): *δ* = 182.19, 152.92, 152.87, 152.04, 151.99, 151.09, 150.31, 150.23, 144.04, 135.17, 134.01, 133.18, 132.57, 132.34, 132.21, 132.11, 132.00, 131.97, 131.88, 131.73, 131.06 (two signals), 130.26, 130.24, 130.13, 130.11, 129.49, 128.86, 127.68, 126.79, 126.32, 126.24, 123.70, 122.99, 122.80, 122.60, 122.59, 122.46, 122.41, 118.64, 118.43, 105.85, 88.64, 33.88, 31.39. HRMS (ESI+): *m/z* = 873.27895 [M + H^+^], calcd. for [C_58_H_34_BN_6_O_3_^+^]: *m/z* = 873.27800.

**SubPc-Triangulene dyad (2)**

A flame-dried flask was charged with a suspension of **SubPc-Cl** (49 mg, 114 µmol) and **8a** (88 mg, 180 µmol) in anhydrous toluene (5 mL) and anhydrous pyridine (5 mL) under N_2_-atmosphere and refluxed for 15 days, after which the reaction mixture was cooled down and dried *in vacuo*. Purification by flash column chromatography (SiO_2_, 10% EtOAc/toluene) repeated twice, followed flash column chromatography (SiO_2_, 4.5% EtOAc/toluene) yielded the title compound **2** (15 mg, 15%) as a dark purple solid. M.p.: >300 °C. ^1^H NMR (600 MHz, Benzene-*d_6_*): *δ* = 9.05 (br s, 2H), 8.91 (s, 2H), 8.65 – 8.61 (m, 6H), 7.32 – 7.28 (m, 6H), 1.33 (s, 18H), 1.26 (s, 9H); (one signal missing, masked by solvent signal). ^13^C NMR (151 MHz, Benzene-*d_6_*): Strong aggregation of **2** did not allow recording of an unequivocal ^13^C NMR spectrum. HRMS (ESI+): *m/z* = 885.37225 [M + H^+^], calcd. for [C_58_H_46_BN_6_O_3_^+^]: *m/z* = 855.37190.

**12-((Trimethylsilyl)ethynyl)dibenzo[*cd*,*mn*]pyrene-4,8-dione (3)**

Argon-degassed THF (25 mL) was cannulated into a sealed argon-flushed flask containing **7** (153 mg, 337 µmol), Pd(PPh_3_)_2_Cl_2_ (24 mg, 34 µmol), CuI (8.5 mg, 44 µmol) and Cs_2_CO_3_ (220 mg, 674 µmol). Trimethylsilylacetylene (0.14 mL, 1.01 mmol) was added, and the solution was degassed for 2 min under sonication. The resulting reaction mixture was heated to 50 °C, stirred for 4 h and poured into sat. aq. NH_4_Cl (50 mL) and extracted with CH_2_Cl_2_ (3 x 75 mL). The combined organic fractions were dried over MgSO_4_, filtered and concentrated *in vacuo*. Purification by flash column chromatography (SiO_2_, 1 – 2% MeOH/CH_2_Cl_2_) yielded the title compound **3** (98 mg, 72%) as a deep-red solid. R*_f_*  = 0.15 (CH_2_Cl_2_). M.p.: Decomp. >200 °C. ^1^H NMR (600 MHz, CD_2_Cl_2_): *δ* = 9.02 (dd, *J* = 8.5, 1.2 Hz, 2H), 8.90 (dd, *J* = 7.1, 1.2 Hz, 2H), 8.75 (d, *J* = 7.7 Hz, 2H), 7.95 (dd, *J* = 8.5 Hz, 7.1 Hz, 2H), 7.80 (t, *J* = 7.7 Hz, 1H), 0.49 (s, 9H) ppm. ^13^C NMR (151 MHz, CD_2_Cl_2_): *δ* = 182.21, 135.31, 135.20, 133.43, 133.12, 132.23, 130.96, 129.18, 128.97, 127.91, 126.67, 124.33, 117.48, 113.12, 100.45, 0.03 ppm. HRMS (ESI+): *m/z* = 403.11509 [M + H^+^], calcd. for [C_27_H_19_O_2_Si^+^]: *m/z* = 403.11488.

**12-((Triisopropylsilyl)ethynyl)dibenzo[*cd*,*mn*]pyrene-4,8-dione (4)**

Argon-degassed THF (25 mL) was cannulated into a sealed argon-flushed flask containing **7** (135 mg, 297 µmol), Pd(PPh_3_)_2_Cl_2_ (23 mg, 33 µmol), CuI (5.7 mg, 30 µmol) and Cs_2_CO_3_ (193 mg, 594 µmol). (Triisopropylsilyl)acetylene (0.18 mL, 799 µmol) was added, and the solution was degassed for 2 min under sonication. The resulting reaction mixture was heated to 55 °C, stirred for 3.5 h and poured into sat. aq. NH_4_Cl (50 mL) and extracted with CH_2_Cl_2_ (3 x 75 mL). The combined organic fractions were dried over MgSO_4_, filtered and concentrated *in vacuo*. Purification by flash column chromatography (SiO_2_, 1 – 2% MeOH/CH_2_Cl_2_) yielded the title compound **4** (89 mg, 62%) as a deep-red solid. R*_f_*  = 0.17 (CH_2_Cl_2_). M.p.: Decomp. >200 °C. ^1^H NMR (500 MHz, CD_2_Cl_2_): *δ* = 9.09 (dd, *J* = 8.6, 1.1 Hz, 2H), 8.91 (dd, *J* = 7.1, 1.1 Hz, 2H), 8.75 (d, *J* = 7.7 Hz, 2H), 7.97 (dd, *J* = 8.6, 7.1, 2H), 7.80 (t, *J* = 7.7, 1H), 1.44 – 1.28 (m, 21H) ppm. ^13^C NMR (151 MHz, CD_2_Cl_2_): *δ* = 182.78, 135.30, 135.14, 133.41, 133.10, 132.40, 130.92, 129.18, 128.93, 127.93, 126.71, 124.68, 117.33, 110.32, 102.54, 19.07, 11.91 ppm. HRMS (ESI+): *m/z* = 487.20906 [M + H^+^], calcd. for [C_33_H_31_O_2_Si^+^]: *m/z* = 487.20878.

**2,6,10-tri-*tert*-butyl-12-((triisopropylsilyl)ethynyl)dibenzo[*cd*,*mn*]pyrene-4,8-dione (5)**

An argon-flushed sealed flask was charged with a solution of **8b** (50 mg, 80 µmol), Pd(MeCN)_2_Cl_2_ (3.8 mg, 14 µmol), (*t*Bu)_3_P•HBF_4_ (9.3 mg, 32 µmol) and CuI (3.5 mg, 18 µmol) in argon-degassed diisopropylamine (10 mL). (Triisopropylsilyl)acetylene (55 µL, 241 µmol) was added, and the resulting reaction mixture was additionally degassed for 5 min under sonication, before it was heated to 50 °C and let to stir for 21 h. The crude mixture was poured into sat. aq. NH_4_Cl (50 mL), extracted with CH_2_Cl_2_ (3 x 50 mL), washed with brine (50 mL), dried over MgSO_4_, filtered and dried *in vacuo*. Purification by flash column chromatography (SiO_2_, 50% CH_2_Cl_2_/cyclohexane) yielded the title compound **5** (11 mg, 22%) as a dark red solid. M.p.: Decomp. >200 °C. R*_f_*  (50% CH_2_Cl_2_/cyclohexane) = 0.52. ^1^H NMR (500 MHz, CD_2_Cl_2_): *δ* = 9.09 (d, *J* = 2.0 Hz, 2H), 9.06 (d, *J* = 2.0 Hz, 2H), 8.85 (s, 2H), 1.59 (s, 18H), 1.54 (s, 9H), 1.36 – 1.30 (m, 21 H) ppm. ^13^C NMR (126 MHz, CD_2_Cl_2_): *δ* = 183.42, 152.53, 151.05, 133.25, 132.96, 131.97, 131.08, 130.39, 129.95, 129.41, 124.77, 122.87, 116.87, 108.94, 103.24, 36.07, 35.81, 31.41, 31.30, 19.15, 11.88 ppm. HRMS (ESI+): *m/z* = 655.39657 [M + H^+^], calcd. for [C_45_H_55_O_2_Si^+^]: *m/z* = 655.39658.

**2,6,10-tri-*tert*-butyl-12-chlorodibenzo[*cd*,*mn*]pyrene-4,8-dione (6)**

An argon-degassed solution of anhydrous THF (4 mL) and anhydrous pyridine (1 mL) was cannulated into a flame-dried and argon-flushed sealed flask containing **8b** (42.5 mg, 68.3 µmol), Pd(PPh_3_)_2_Cl_2_ (11.5 mg, 16.3 µmol), CuI (2.0 mg, 10 µmol) and LiCl (10.1 mg, 238 µmol). The resulting reaction mixture was heated to 55 °C and stirred for 39 h, before being poured into sat. aq. NH_4_Cl (30 mL), extracted with CH_2_Cl_2_ (3 x 25 mL), washed with brine (30 mL), dried over MgSO_4_, filtered and dried *in vacuo*. Purification by flash column chromatography (SiO_2_, 50% CH_2_Cl_2_/cyclohexane) yielded the title compound **6** (5 mg, 14%) as a red solid). M.p.: Decomp. >200 °C. R*_f_*  (50% CH_2_Cl_2_/cyclohexane) = 0.55. ^1^H NMR (500 MHz, CD_2_Cl_2_): *δ* = 9.09 (d, *J* = 2 Hz, 2H), 8.93 (d, *J* = 2 Hz, 2H), 8.85 (s, 2H), 1.60 (s, 18H),1.54 (s, 9H) ppm. ^13^C NMR (126 MHz, CD_2_Cl_2_): 183.05, 152.38, 151.20, 133.28, 132.27, 130.79, 130.49, 129.37, 129.36, 129.13, 127.58, 125.53, 115.82, 36.18, 35.78, 31.40, 31.18 ppm. HRMS (APCI+): *m/z* = 509.22357 [M + H^+^], calcd. for [C_34_H_34_O_2_Cl^+^]: *m/z* = 509.22418.

# NMR spectra

Figure S1: ^1^H NMR (600 MHz, CD_2_Cl_2_) spectrum of **3** (1.7 x 10^-3^ M).

Figure S2: ^13^C NMR (151 MHz, CD_2_Cl_2_) spectrum of **3** (1.7 x 10^-3^ M). Strong aggregation of **3** did not allow data-collection at higher concentrations as severe line-broadening was observed.

Figure S3: ^1^H-^1^H DQF-COSY (600 MHz, CD_2_Cl_2_) spectrum (aromatic region selected) of **3**.

Figure S4: ^1^H-^13^C HSQC (600 MHz, CD_2_Cl_2_) spectrum (aromatic region selected) of **3**.

Figure S5: ^1^H-^13^C HBMC (600 MHz, CD_2_Cl_2_) spectrum (aromatic region selected) of **3**.

Figure S6: ^1^H-^13^C HBMC (600 MHz, CD_2_Cl_2_) spectrum (aliphatic region selected) of **3**.

Figure S7: Assignment of recorded ^1^H- and ^13^C NMR resonances for **3**, based on corresponding 2D correlation spectroscopic data.

Figure S8: ^1^H NMR (600 MHz, Benzene-d_6_) spectrum of **1** (0.71 x 10^-^**^3^** M, saturated).

******

Figure S9: ^13^C NMR (151 MHz, Benzene-d_6_) spectrum of **1** (0.71 x 10^-^**^3^** M, saturated). Strong aggregation of **1** did not allow data-collection at higher concentrations due to precipitation.

Figure S10: ^1^H-^1^H DQF-COSY (600 MHz, Benzene-d_6_) spectrum (aromatic region selected) of **1**.

Figure S11: ^1^H-^13^C HSQC (600 / 151 MHz, Benzene-d_6_) spectrum (aromatic region selected, 9.45 – 8.60 ppm) of **1**.

**

Figure S12: ^1^H-^13^C HSQC (600 / 151 MHz, Benzene-d_6_) spectrum (aromatic region selected 7.85 –5.65 ppm) of **1**.

**

Figure S13: ^1^H-^13^C HSQC (600 / 151 MHz, Benzene-d_6_) spectrum (aliphatic region selected) of **1**.

Figure S14: ^1^H-^13^C HMBC (600 / 151 MHz, Benzene-d_6_) spectrum (aromatic region selected, overview) of **1**.

Figure S15: ^1^H-^13^C HMBC (600 / 151 MHz, Benzene-d_6_) spectrum (aromatic region selected, zoom) of **1**.

Figure S16: ^1^H-^13^C HMBC (600 / 151 MHz, Benzene-d_6_) spectrum (aromatic region selected, zoom) of **1**.

Figure S17: ^1^H-^13^C HMBC (600 / 151 MHz, Benzene-d_6_) spectrum (aliphatic region selected) of **1**.

Figure S18: Assignment of recorded ^1^H- and ^13^C NMR resonances for **1**, based on corresponding 2D correlation spectroscopic data.

Figure S19: ^1^H NMR (600 MHz, CD_2_Cl_2_) spectrum of **4** (1.6 x 10^-3^ M). Strong aggregation of **4** did not allow data-collection at higher concentrations as severe line-broadening was observed.

Figure S20: ^13^C NMR (151 MHz, CD_2_Cl_2_) spectrum of **4** (1.6 x 10^-3^ M). Strong aggregation of **4** did not allow data-collection at higher concentrations as severe line-broadening was observed.

Figure S21: ^1^H-^1^H DQF-COSY (600 MHz, CD_2_Cl_2_) spectrum (aromatic region selected) of **4**.

Figure S22: ^1^H-^13^C HSQC (600 MHz, CD_2_Cl_2_) spectrum (aromatic region selected) of **4**.

Figure S23: ^1^H-^13^C HSQC (600 MHz, CD_2_Cl_2_) spectrum (aliphatic region selected) of **4**.

Figure S24: ^1^H-^13^C HBMC (600 MHz, CD_2_Cl_2_) spectrum (aromatic region selected) of **4**.

Figure S25: ^1^H-^13^C HBMC (600 MHz, CD_2_Cl_2_) spectrum (aliphatic region selected) of **4**.

**

Figure S26: ^1^H NMR (600 MHz, Benzene-d_6_) spectrum of **2**.

Figure S27: ^1^H-^1^H DQF-COSY (600 MHz, Benzene-d_6_) spectrum (aromatic region selected) of **2**.

Figure S28: ^1^H-^13^C HSQC (600 / 151 MHz, Benzene-d_6_) spectrum (aromatic region selected, zoom) of **2**, showing correlation of masked signal under benzene solvent signal. Solvent correlation is suppressed.

Figure S29: ^1^H-^13^C HMBC (600 / 151 MHz, Benzene-d_6_) spectrum (aromatic region selected, zoom) of **2**, showing correlation of masked signal under benzene solvent signal.

Figure S30: ^1^H NMR (500 MHz, CD_2_Cl_2_) spectrum of **6**.

Figure S31: ^13^C NMR (126 MHz, CD_2_Cl_2_) spectrum of **6**.

Figure S32: ^1^H-^1^H COSY (500 MHz, CD_2_Cl_2_) spectrum (aromatic region selected) of **6**.

Figure S33: ^1^H NMR (500 MHz, CD_2_Cl_2_) spectrum of **5**.

Figure S34: ^13^C NMR (126 MHz, CD_2_Cl_2_) spectrum of **5**.

Figure S35: ^1^H-^1^H COSY (500 MHz, CD_2_Cl_2_) spectrum (aromatic region selected) of **5**.

HRMS of dyads **1** and **2**


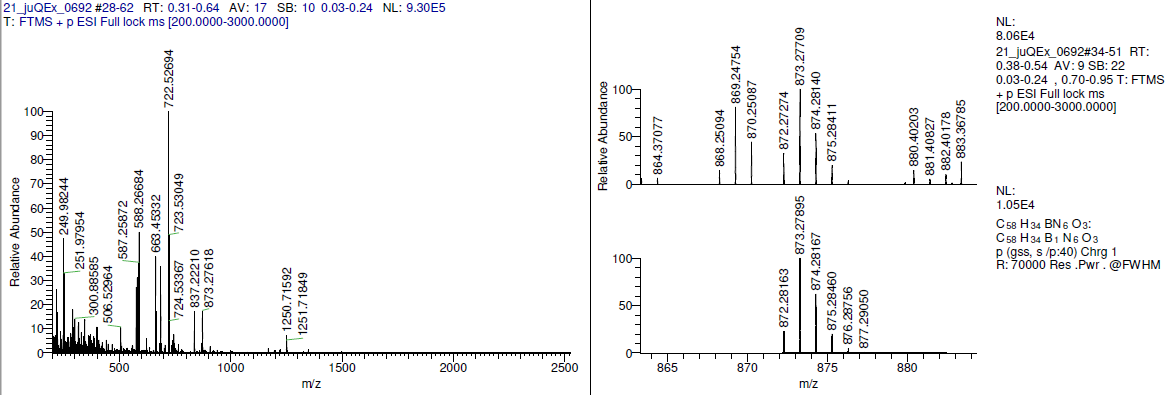


Figure S36: HRMS spectra of **1**.


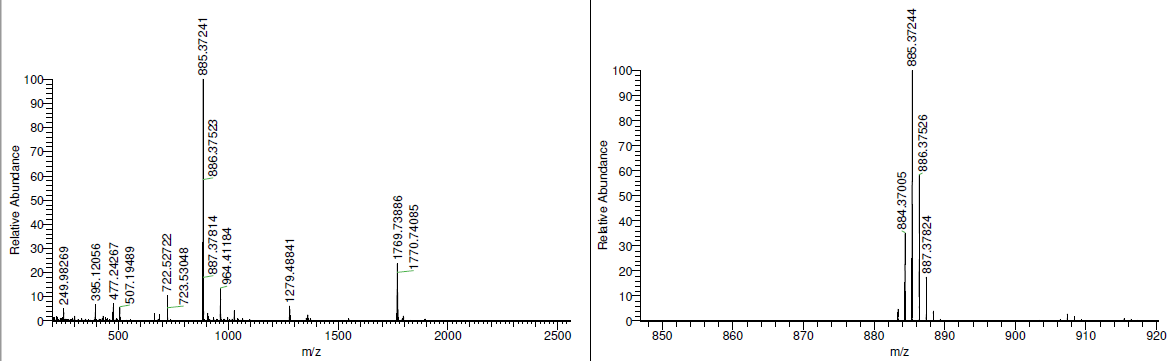


Figure S37: HRMS spectra of **2**.

# UV-vis and fluorescence data

Figure S38: UV-vis absorption spectrum of **1** in CH_2_Cl_2_.

Figure S39: UV-vis absorption spectrum of **1** in toluene.

Figure S40: Stacked UV-vis absorption spectra **1** in toluene (purple) and CH_2_Cl_2_ (blue).

Figure S41: UV-vis absorption spectrum of **2** in toluene.

Figure S42: UV-vis absorption spectrum of **2** in CH_2_Cl_2_.

Figure S43: Stacked UV-vis absorption spectra of **2** in toluene (dashed) and CH_2_Cl_2_ (full).

Figure S44: UV-vis absorption spectrum of **3** in CH_2_Cl_2_.

Figure S45: UV-vis absorption spectrum of **4** in CH_2_Cl_2_.

Figure S46: UV-vis absorption spectrum of **5** in CH_2_Cl_2_.

Figure S47: UV-vis absorption spectrum of **6** in CH_2_Cl_2_.

******

Figure S48: UV-vis absorption and normalized emission spectra of **1** in toluene; excitation at 620 nm.

Figure S49: Excitation spectra of **1** recorded at 645 nm (dashed) and 700 nm (full) emission in toluene.

Figure S50: UV-vis absorption and normalized emission spectra of **2** in toluene; excitation at 566 nm.

Figure S51: UV-vis absorption and normalized emission spectra of **2** in CH_2_Cl_2_; excitation at 566 nm.

Figure S52: Excitation spectra of **2** recorded at 620 nm (black) and 680 nm (red) emission in toluene.

Figure S53: UV-vis absorption and normalized emission spectra of **3** in CH_2_Cl_2_; excitation at 522 nm.

Figure S54: UV-vis absorption and normalized emission spectra of **4** in CH_2_Cl_2_; excitation at 525 nm.

Figure S55: UV-vis absorption and normalized emission spectra of **5** in CH_2_Cl_2_; excitation at 522 nm.

Figure S56: UV-vis absorption and normalized emission spectra of **6** in CH_2_Cl_2_; excitation at 508 nm.

# Electrochemistry

Figure S57: Stacked cyclic voltammograms of **4** (left) and stacked differential pulse voltammograms of **4** (right) (0.52 – 0.10 mM); potentials vs. Fc/Fc^+^. Solvent: CH_2_Cl_2_; supporting electrolyte: 0.1 M Bu_4_NPF_6_; scan rate 0.1 V/s.

Figure S58: Stacked cyclic voltammograms and differential pulse voltammograms of **4** (left, conc. 0.52 mM) and **3** (right, 0.32 mM); potentials vs. Fc/Fc^+^. Solvent: CH_2_Cl_2_; supporting electrolyte: 0.1 M Bu_4_NPF_6_; scan rate 0.1 V/s.

Figure S59: Stacked cyclic voltammograms and differential pulse voltammograms of **5** (left, conc. 0.57 mM) and **6** (right, 0.50 mM); potentials vs. Fc/Fc^+^. Solvent: CH_2_Cl_2_; supporting electrolyte: 0.1 M Bu_4_NPF_6_; scan rate 0.1 V/s.

Figure S60: Stacked cyclic voltammograms and differential pulse voltammograms of **2** (left, conc. 0.41 mM) and **1** (right, 0.50 mM); potentials vs. Fc/Fc^+^. Solvent: CH_2_Cl_2_; supporting electrolyte: 0.1 M Bu_4_NPF_6_; scan rate 0.1 V/s.

# X-Ray crystallographic data


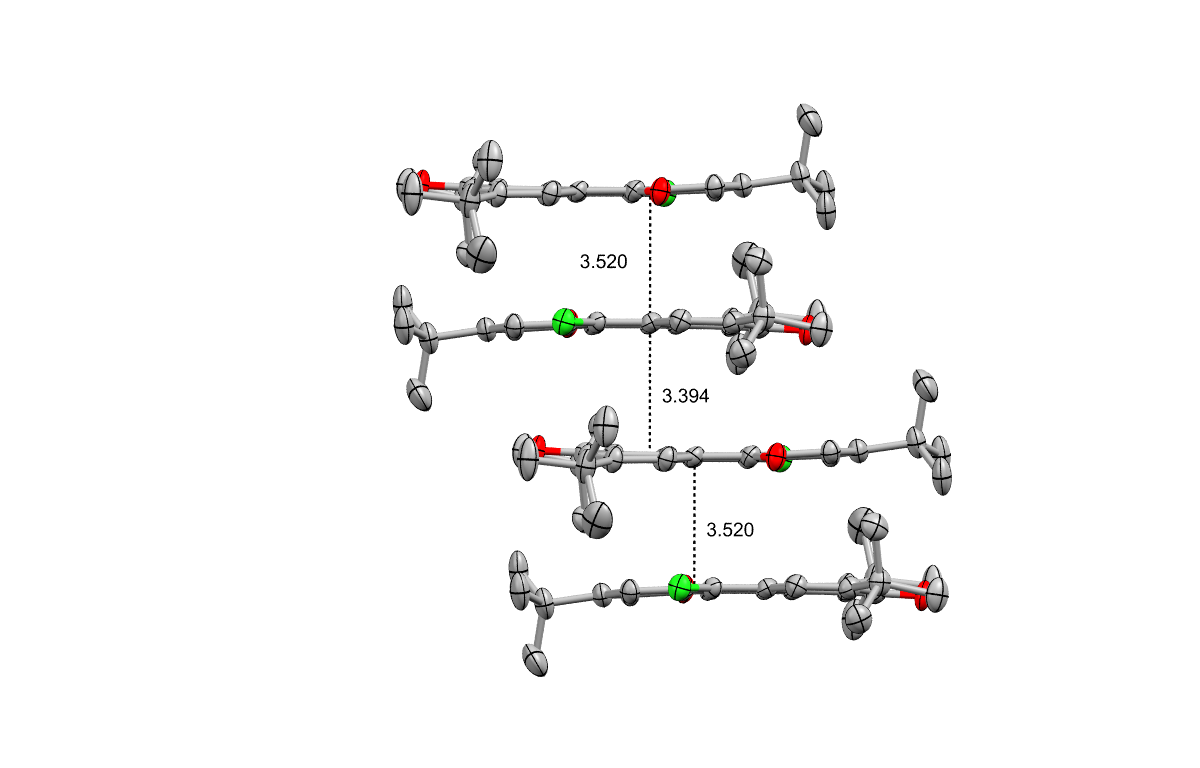


Figure S61: Sideview of **6** crystal packing. Distance in Å measured between central C-atom and triangulene plane. Thermal ellipsoids are displayed at 50% probability level. Hydrogen atoms are omitted for clarity.


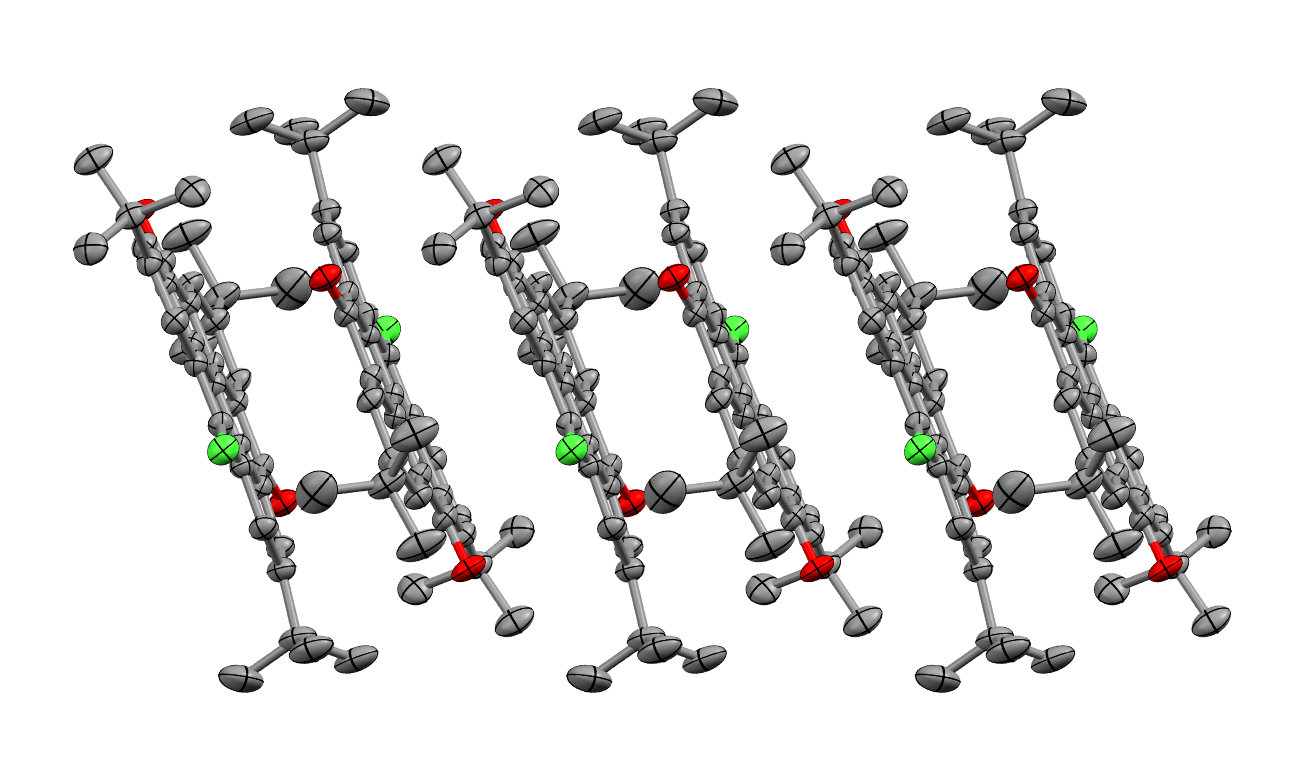


Figure S62: View of crystal packing along the *c*-axis of **6**. Thermal ellipsoids are displayed at 50% probability level. Hydrogen atoms are omitted for clarity.


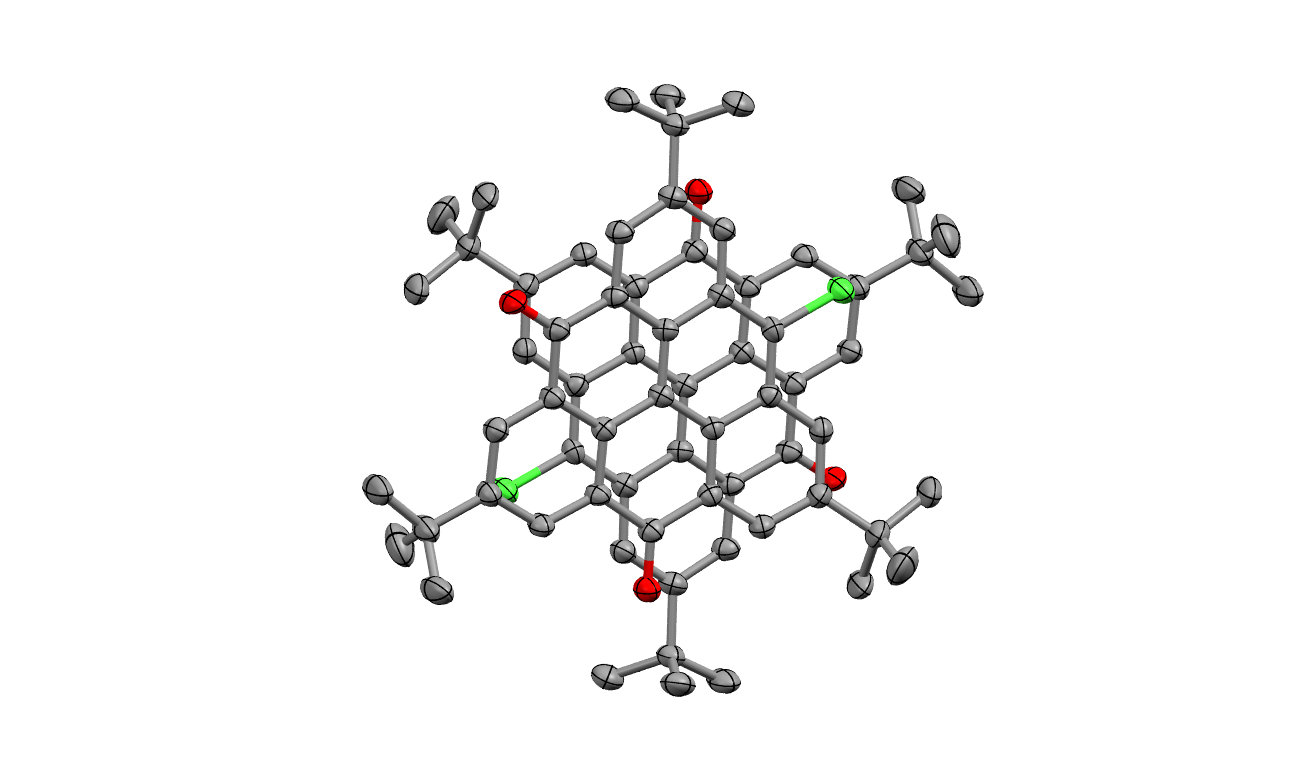


Figure S63: View of crystal packing along the *a*-axis of **6**. Thermal ellipsoids are displayed at 50% probability level. Hydrogen atoms are omitted for clarity.


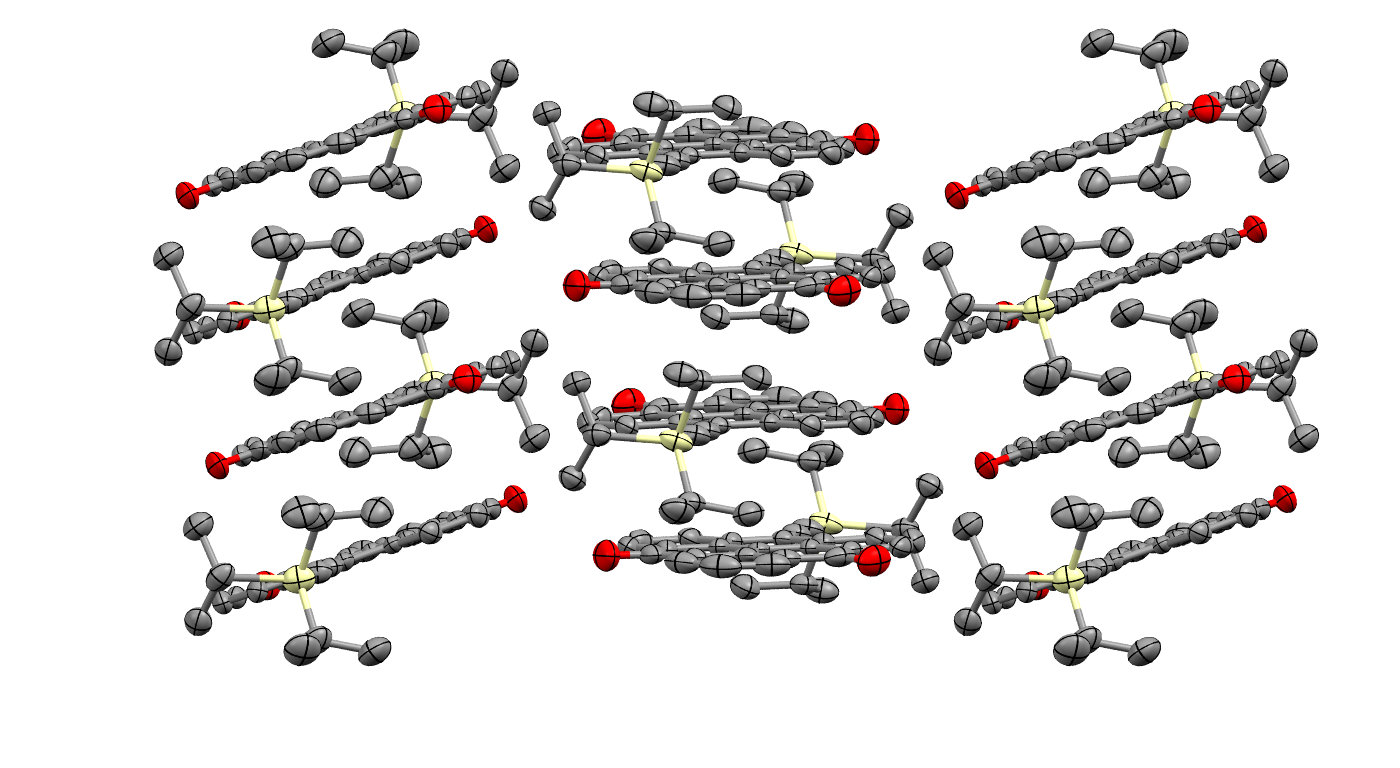


Figure S64: View of crystal packing along the b-axis of **4** crystal packing. Thermal ellipsoids are displayed at 50% probability level. CH_2_Cl_2_ solvent molecules and hydrogen atoms are omitted for clarity.


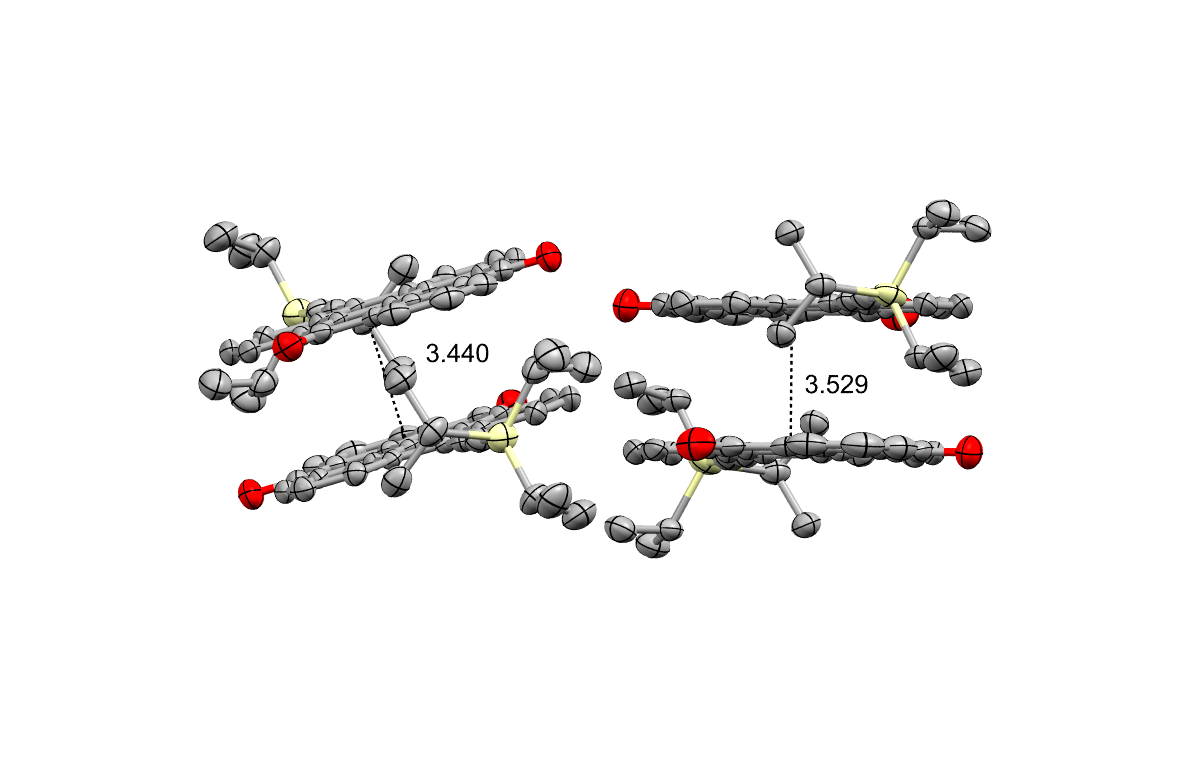


Figure S65: Sideview of **4** crystal packing. Distance in Å measured between central C-atom and triangulene plane. Thermal ellipsoids are displayed at 50% probability level. CH_2_Cl_2_ solvent molecules and hydrogen atoms are omitted for clarity.


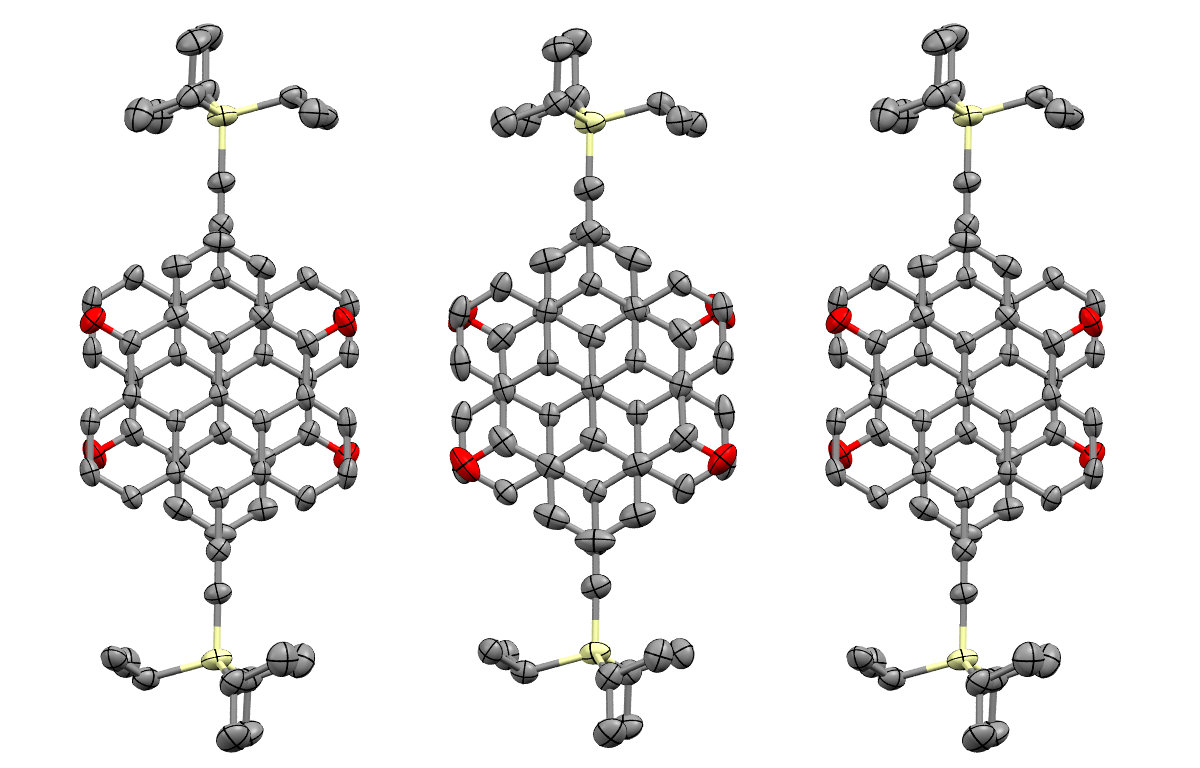


Figure S66: View of crystal packing along the *a*-axis of **4**. Thermal ellipsoids are displayed at 50% probability level. CH_2_Cl_2_ solvent molecules and hydrogen atoms are omitted for clarity.


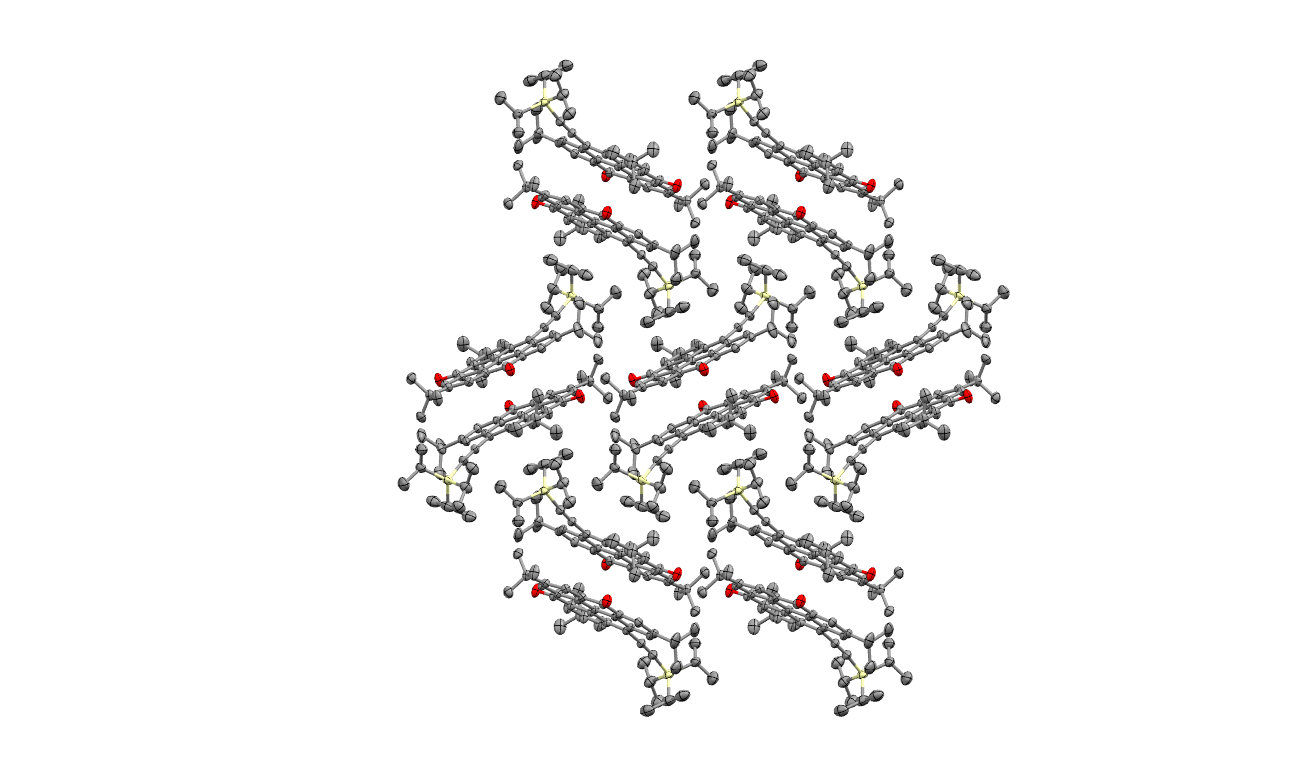


Figure S67: View of crystal packing along the *c*-axis of **5** crystal packing. Thermal ellipsoids are displayed at 50% probability level. Hydrogen atoms are omitted for clarity.


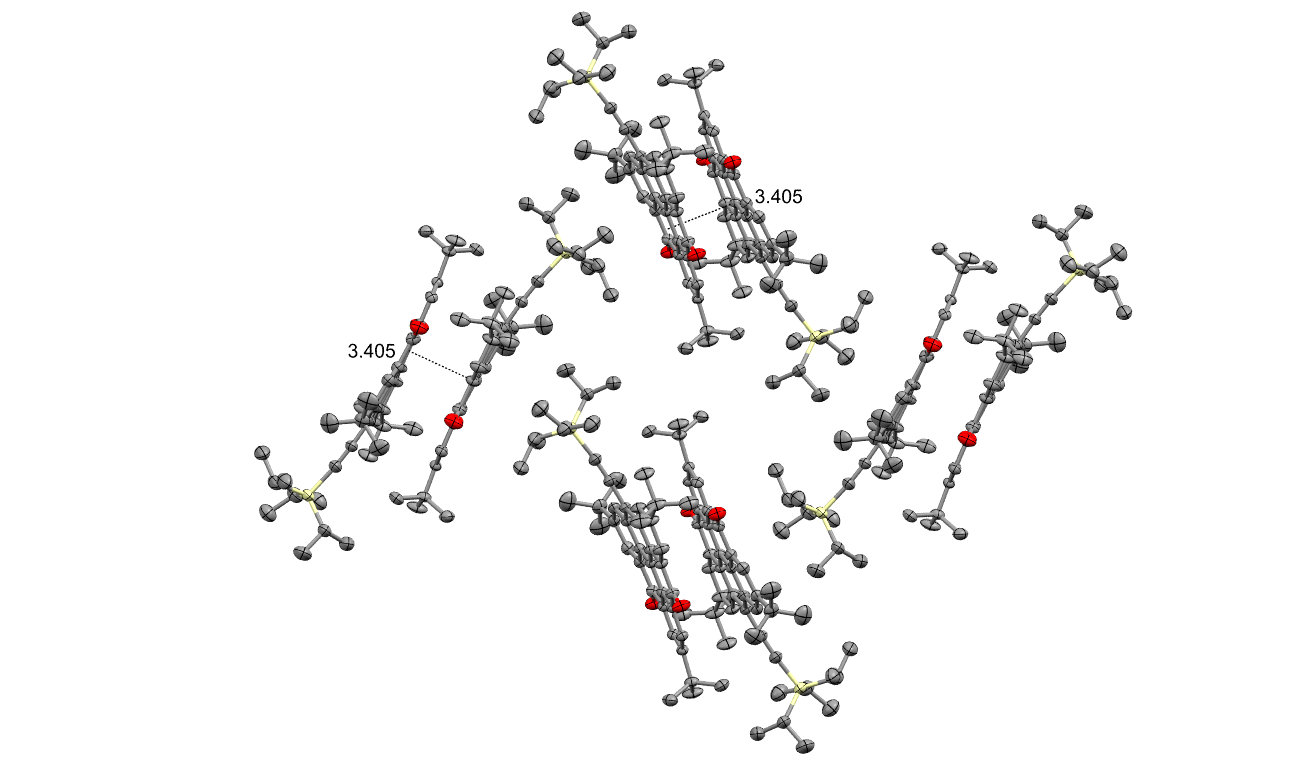


Figure S68: Sideview of **5** crystal packing. Distance in Å measured between central C-atom and triangulene plane. Thermal ellipsoids are displayed at 50% probability level. Hydrogen atoms are omitted for clarity.


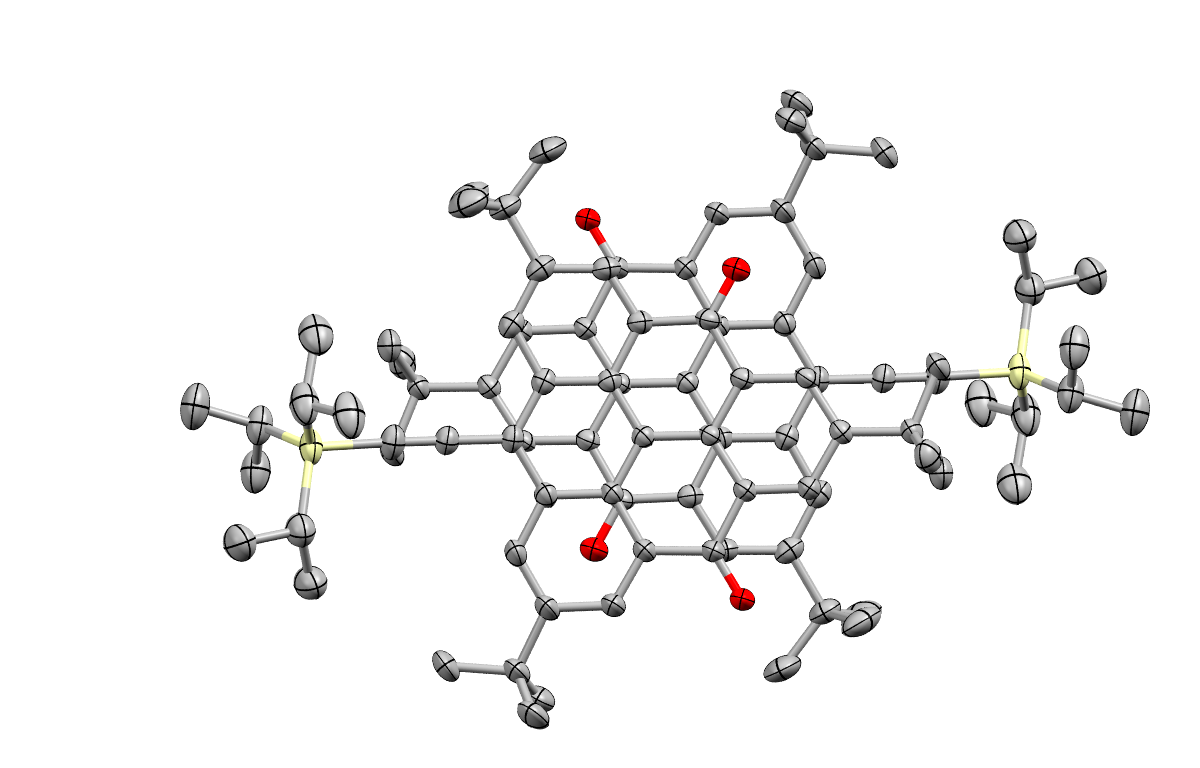


Figure S69: Top view of **5** head-to-head packing. Thermal ellipsoids are displayed at 50% probability level. Hydrogen atoms are omitted for clarity.

*
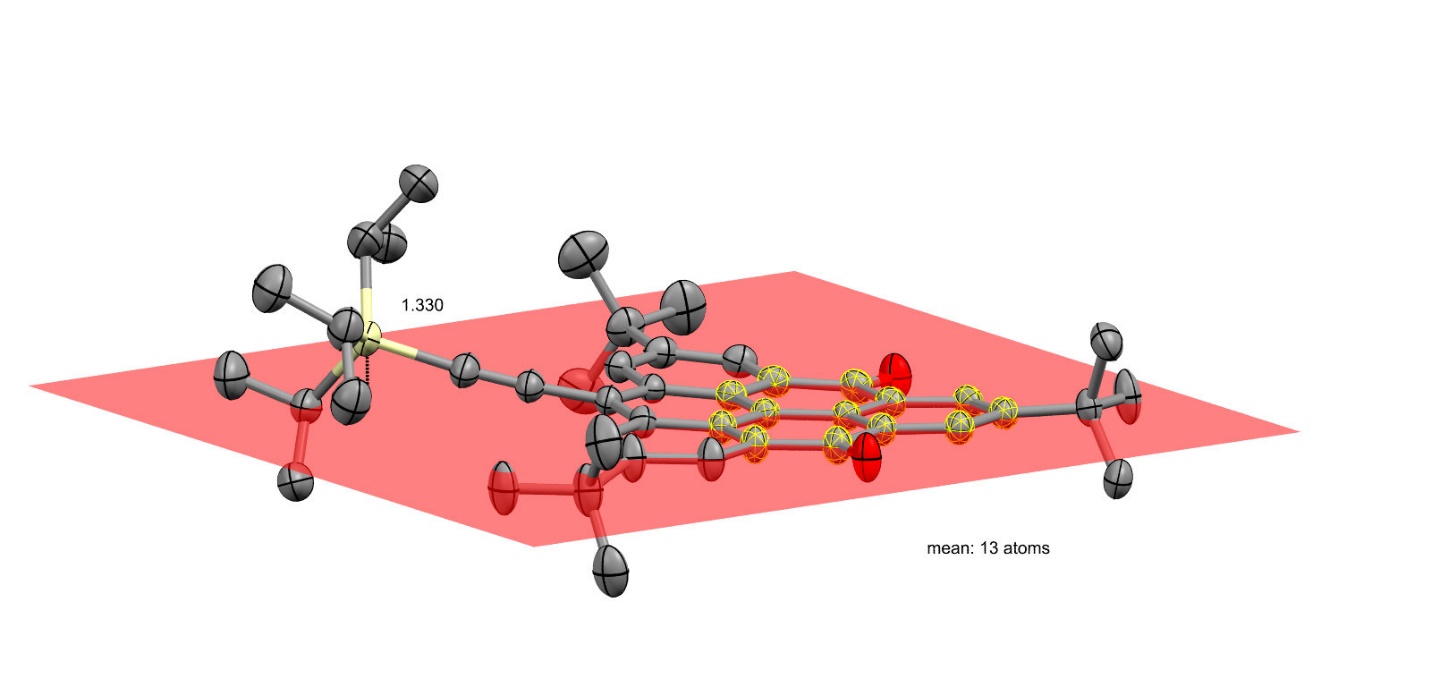
*

Figure S70: Sideview of asymmetric unit of **5**. Distance in Å measured between silicon-atom and triangulene plane (highlighted yellow, mean: 13 atoms). Thermal ellipsoids are displayed at 50% probability level. Hydrogen atoms are omitted for clarity.


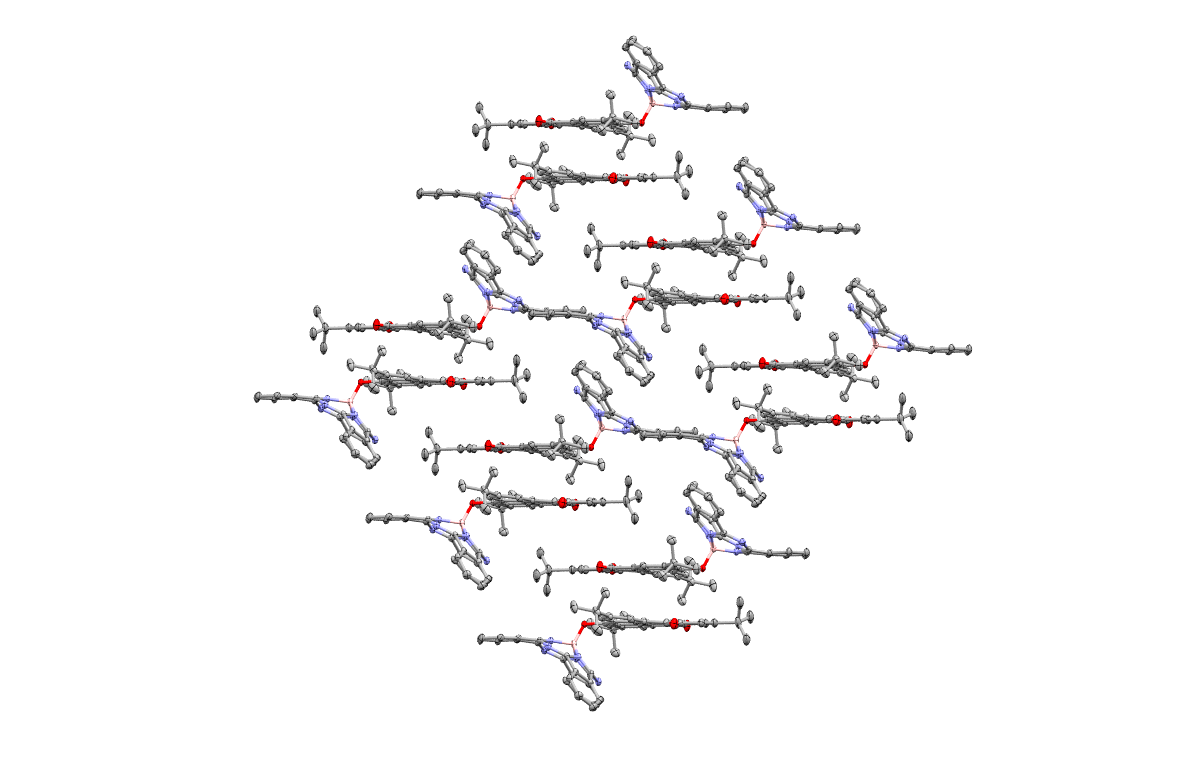


Figure S71: Sideview of **2** crystal packing. Thermal ellipsoids are displayed at 50% probability level. Hydrogen atoms are omitted for clarity.

***
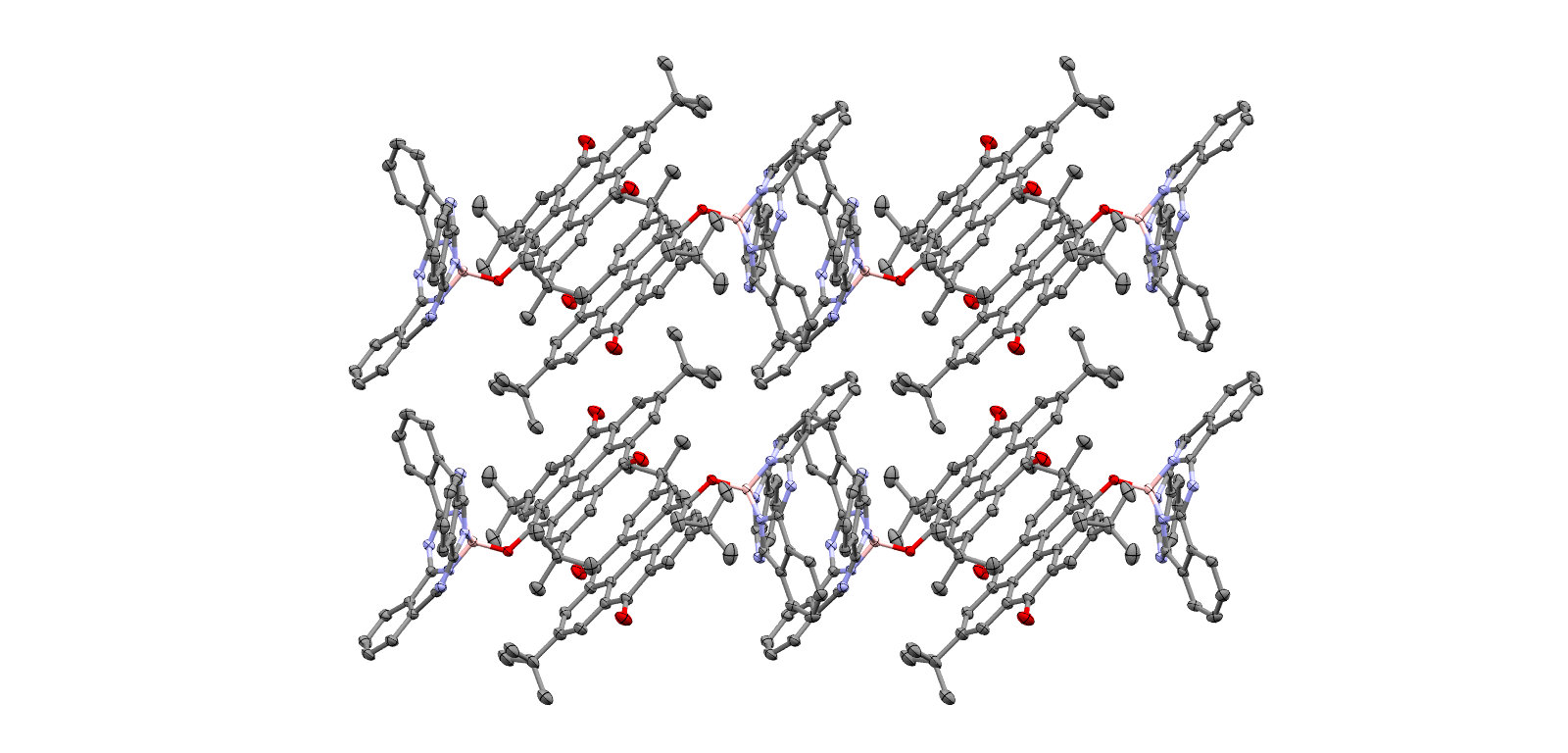
***

Figure S72: View of crystal packing along the *b*-axis of **2** crystal packing. Thermal ellipsoids are displayed at 50% probability level. Hydrogen atoms are omitted for clarity.


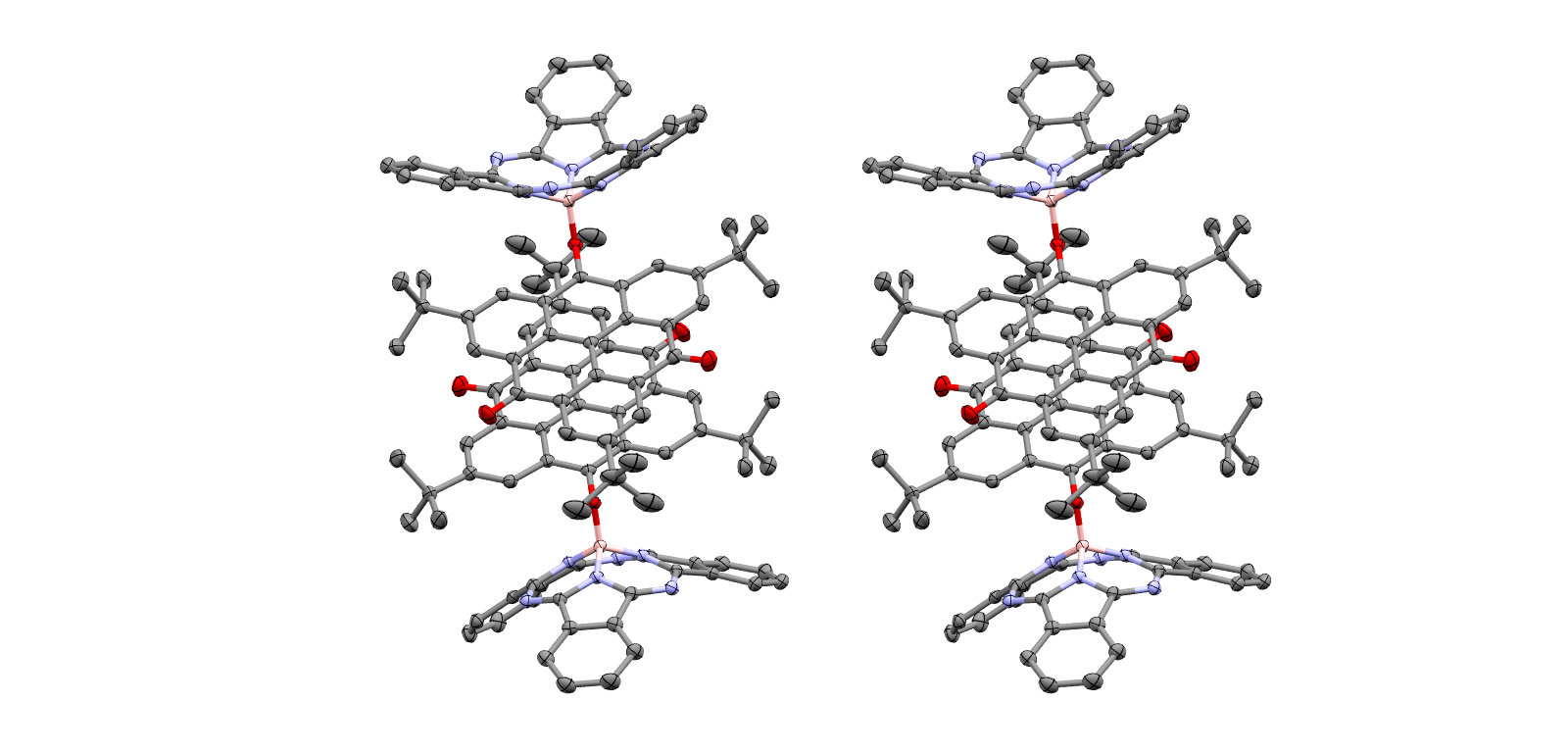


Figure S73: View of crystal packing along the *a*-axis of **2** crystal packing. Thermal ellipsoids are displayed at 50% probability level. Hydrogen atoms are omitted for clarity.


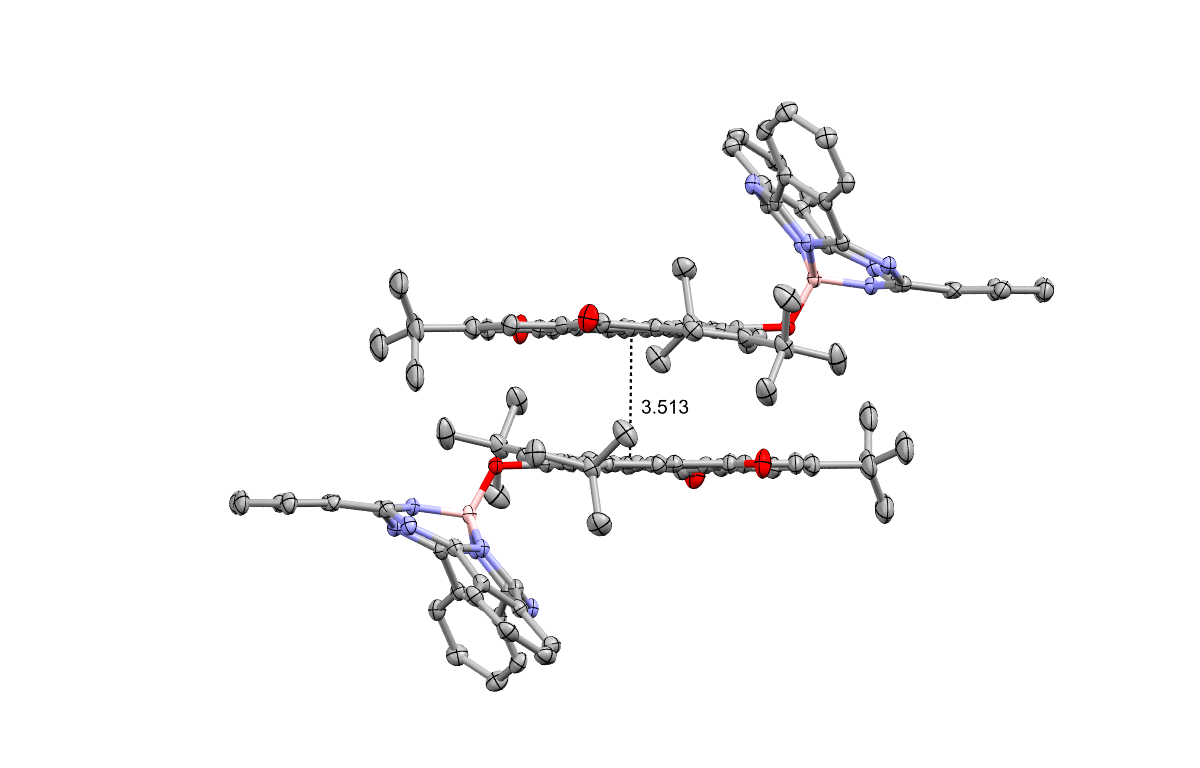


Figure S74: Sideview of **2** crystal packing. Distance in Å measured between central C-atom and triangulene plane. Thermal ellipsoids are displayed at 50% probability level. Hydrogen atoms are omitted for clarity.


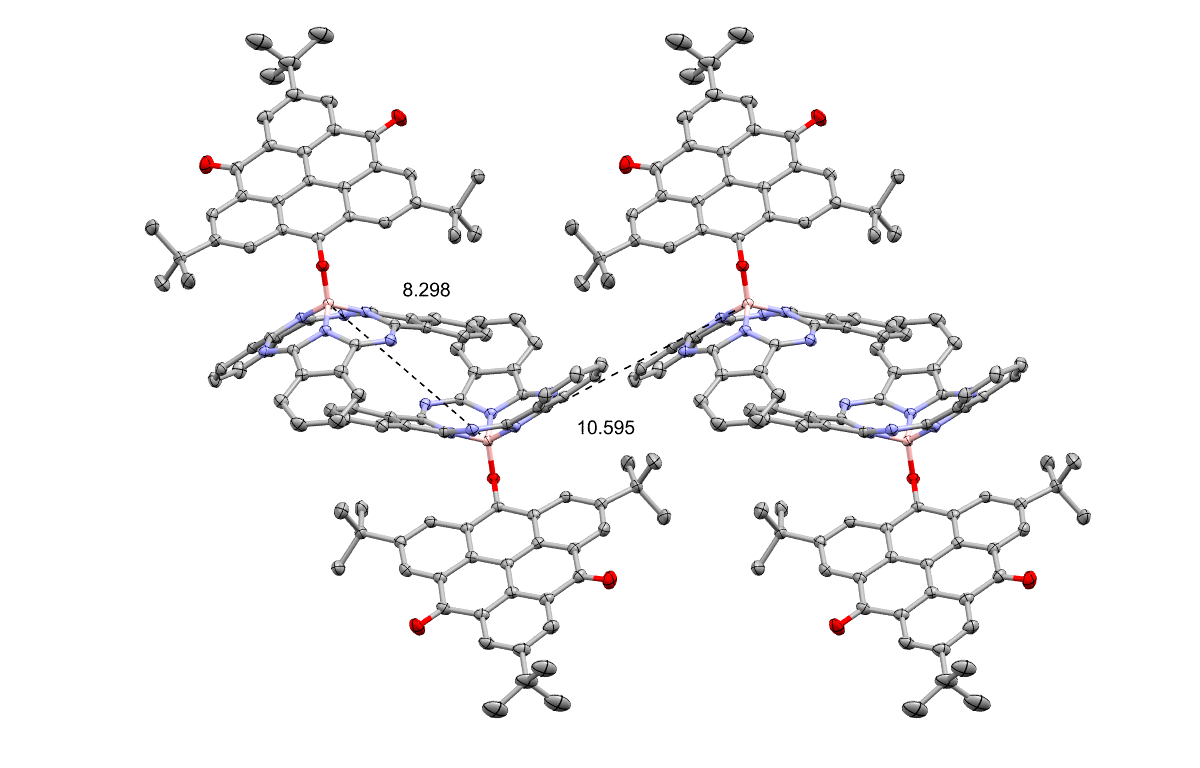


Figure S75: View of crystal packing along the *a*-axis of **2** crystal packing. Distance in Å measured between central boron atoms of the SubPc core. Thermal ellipsoids are displayed at 50% probability level. Hydrogen atoms are omitted for clarity.


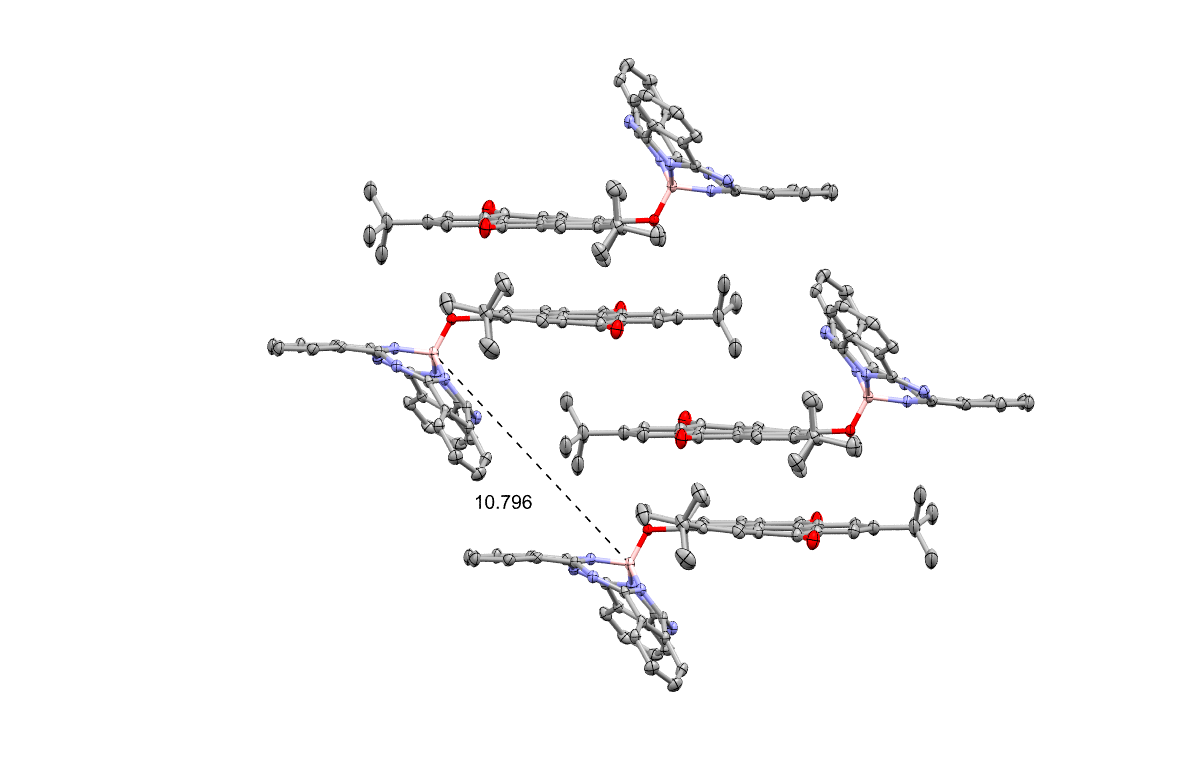


Figure S76: Sideview of **2** crystal packing. Distance in Å measured between central boron atoms of the SubPc core. Thermal ellipsoids are displayed at 50% probability level. Hydrogen atoms are omitted for clarity.


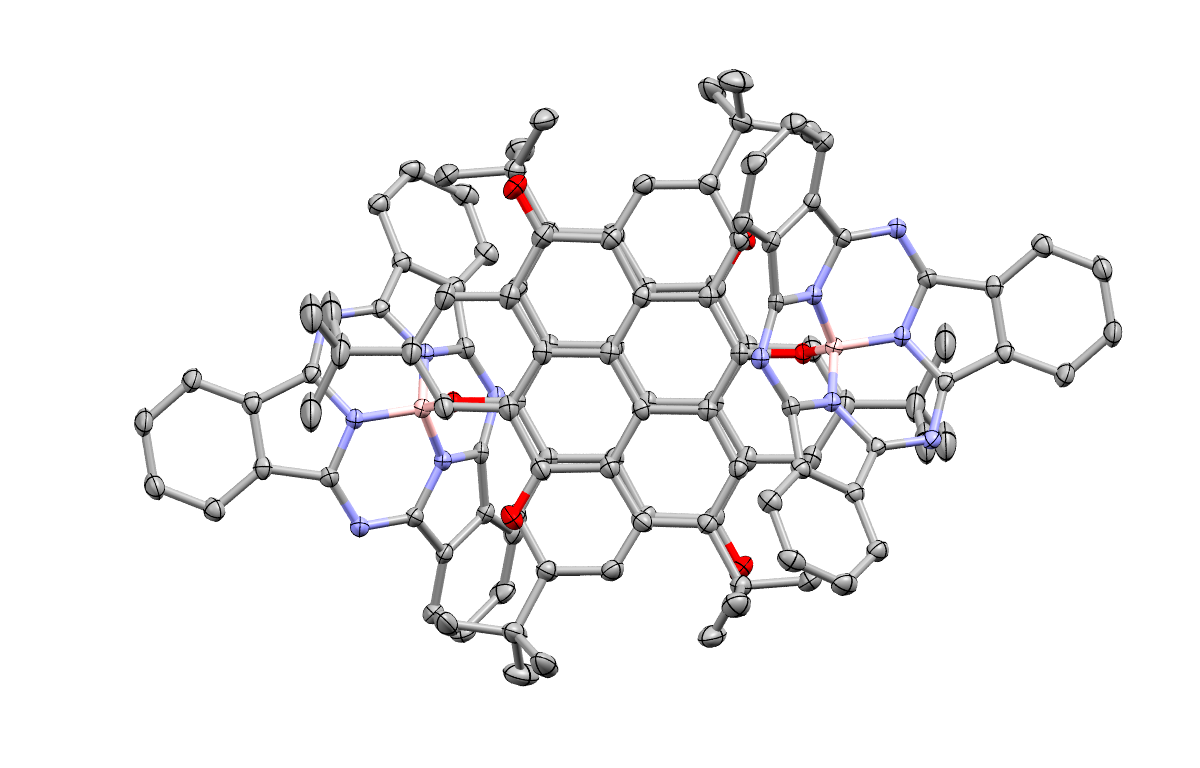


Figure S77: Top view of **2** head-to-head packing. Thermal ellipsoids are displayed at 50% probability level. Hydrogen atoms are omitted for clarity.


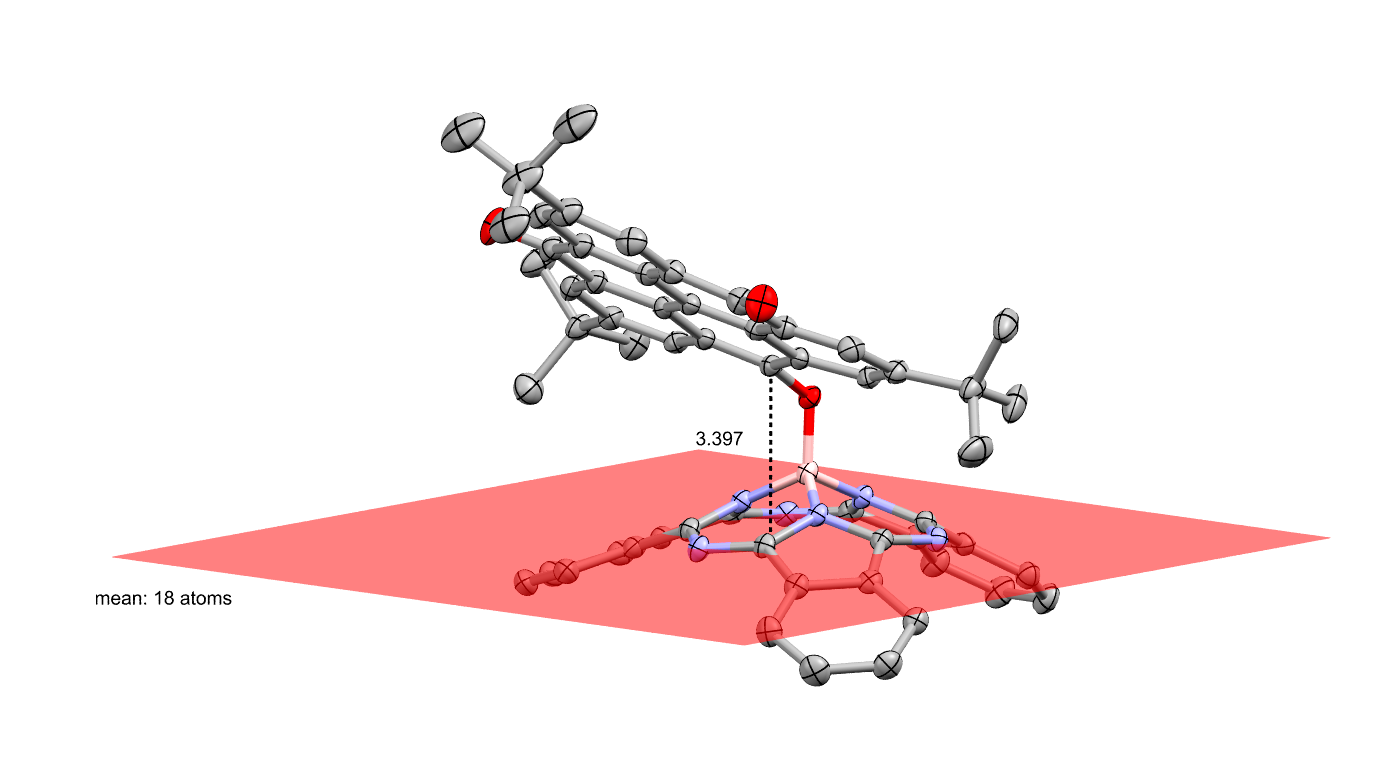


Figure S78: Sideview of asymmetric unit of **2**. Distance in Å measured between the plane of the SubPc central π-system (mean: 18 atoms) and triangulene frame edge-carbon (3.4 Å). Thermal ellipsoids are displayed at 50% probability level. Hydrogen atoms are omitted for clarity.

**2**: (CCDC 2113701):


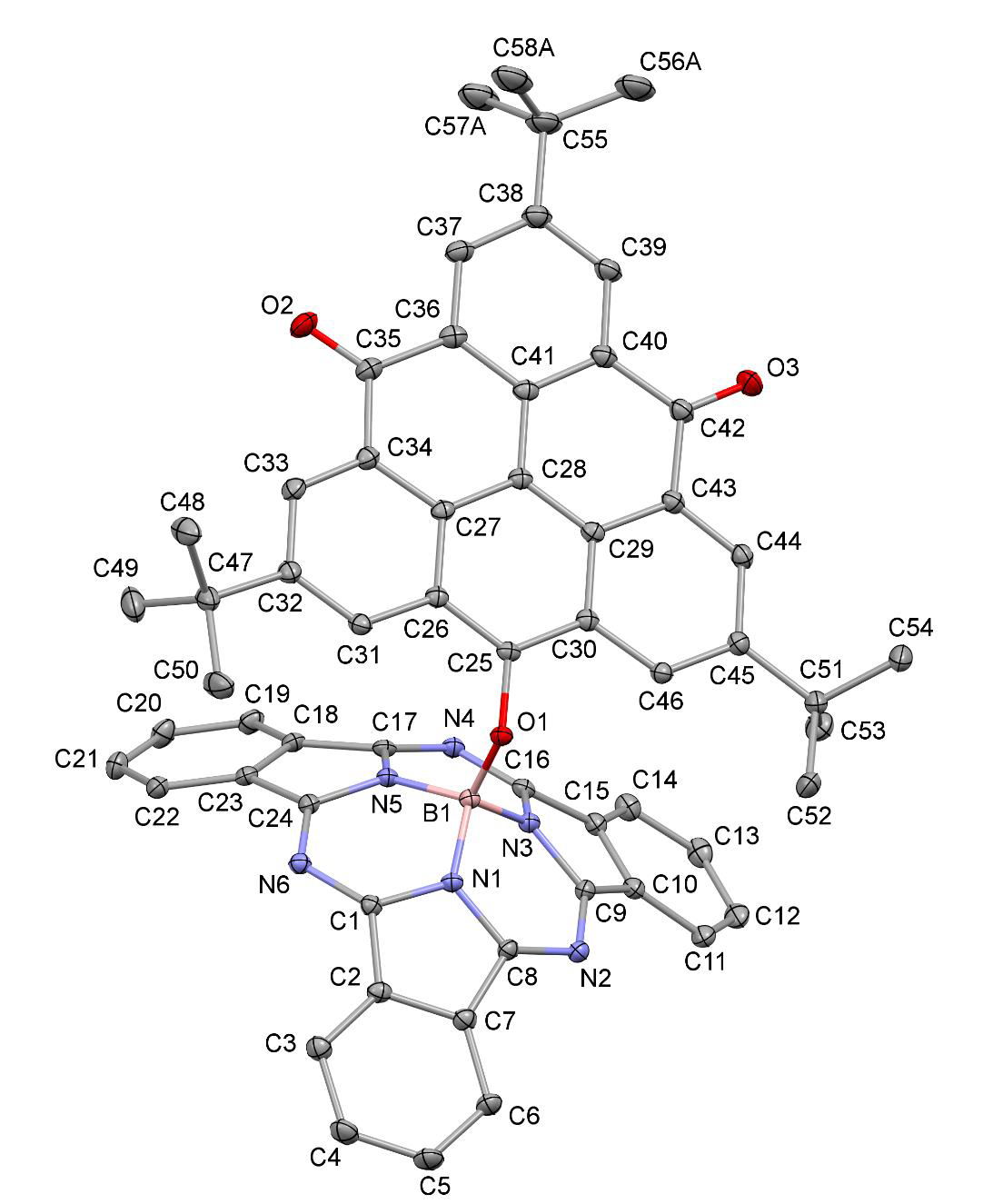


**Experimental**

Single-crystal X-ray diffraction data were collected at 160(1) K on a Rigaku OD XtaLAB Synergy, Dualflex, Pilatus 200K diffractometer using a single wavelength X-ray source (Cu K_α_ radiation: λ= 1.54184 Å) from a micro-focus sealed X-ray tube and an Oxford liquid-nitrogen Cryostream cooler. The selected suitable single crystal was mounted using polybutene oil on a flexible loop fixed on a goniometer head and immediately transferred to the diffractometer. Pre-experiment, data collection, data reduction and analytical absorption correction^[5]^ were performed with the program suite *CrysAlisPro*.^[6]^ Using *Olex2*,^[7]^ the structure was solved with the SHELXT^[8]^ small molecule structure solution program and refined with the *SHELXL2018/3* program package^[9]^ by full-matrix least-squares minimization on F^2^. *PLATON*^[10]^ was used to check the result of the X-ray analysis. For more details about the data collection and refinement parameters, see the CIF file.

**Special features**

A solvent mask^[11]^ was calculated as the residual electron density showed disordered solvent molecules of dichloromethane (166 electrons corresponding to four solvent molecules per unit cell). The solvent molecules were taken into account in the formula moiety and the formula sum (leading to many alerts in the checkCIF report).

| **Crystal data and structure refinement for 2** | |
| --- | --- |
| Empirical formula | C_60_H_49_BN_6_O_3_Cl_4_ |
| Formula weight | 1054.66 |
| Temperature/K | 160(1) |
| Crystal system | triclinic |
| Space group | P-1 |
| a/Å | 10.7964(3) |
| b/Å | 15.4012(5) |
| c/Å | 17.2200(6) |
| α/° | 66.145(3) |
| β/° | 83.692(3) |
| γ/° | 78.859(3) |
| Volume/Å^3^ | 2567.74(15) |
| Z | 2 |
| ρ_calc_g/cm^3^ | 1.364 |
| μ/mm^-1^ | 2.522 |
| F(000) | 1096.0 |
| Crystal size/mm^3^ | 0.24 × 0.11 × 0.05 |
| Radiation | Cu Kα (λ = 1.54184) |
| 2Θ range for data collection/° | 5.616 to 136.494 |
| Index ranges | -13 ≤ h ≤ 12, -18 ≤ k ≤ 18, -20 ≤ l ≤ 20 |
| Reflections collected | 50640 |
| Independent reflections | 9406 [R_int_ = 0.0350, R_sigma_ = 0.0268] |
| Data/restraints/parameters | 9406/145/654 |
| Goodness-of-fit on F^2^ | 1.063 |
| Final R indexes [I>=2σ (I)] | R_1_ = 0.0491, wR_2_ = 0.1255 |
| Final R indexes [all data] | R_1_ = 0.0539, wR_2_ = 0.1289 |
| Largest diff. peak/hole / e Å^-3^ | 1.06/-0.89 |

**4**: (CCDC 2113702):


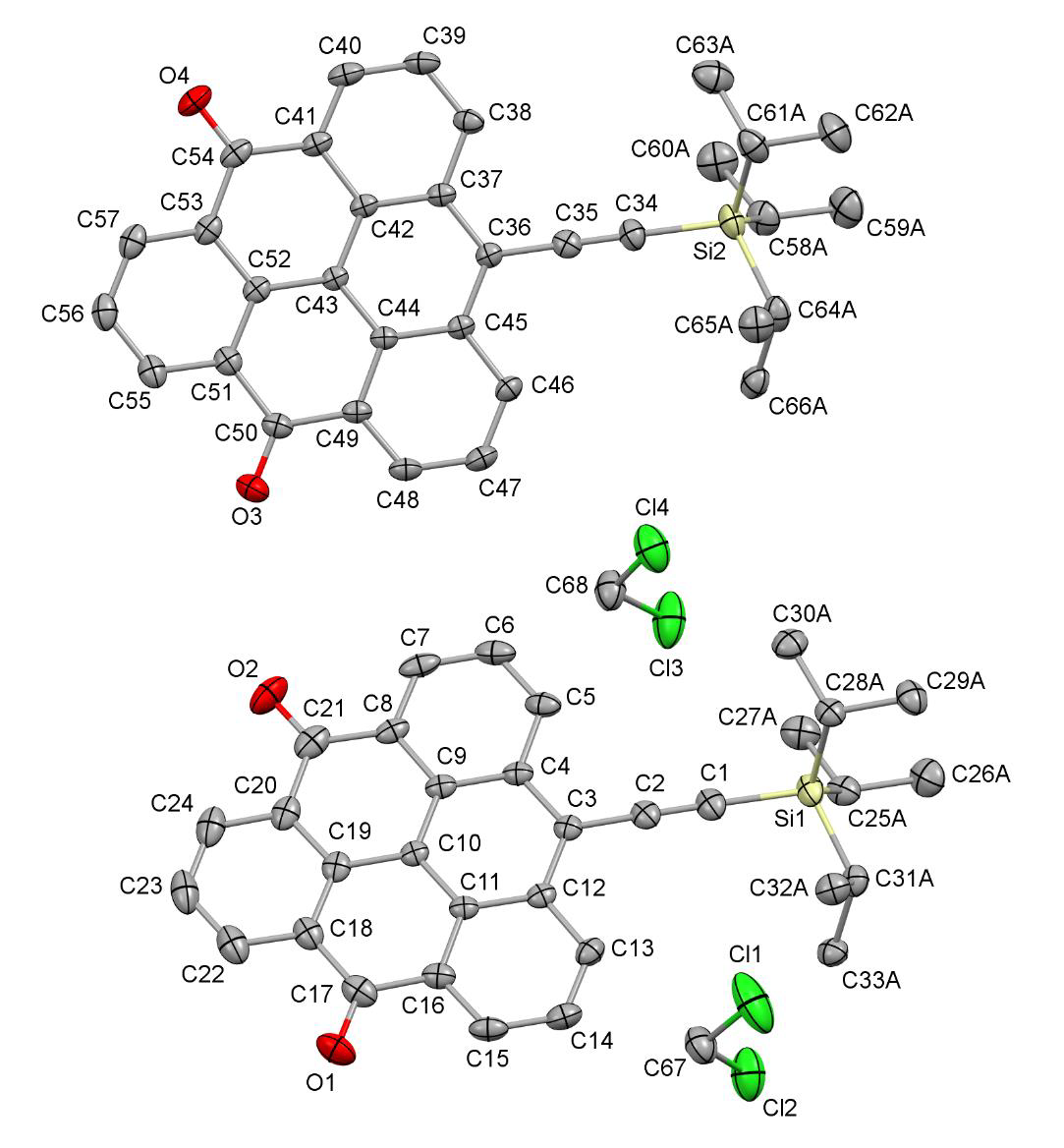


**Experimental**

Single-crystal X-ray diffraction data were collected at 160(1) K on a Rigaku OD SuperNova/Atlas area-detector diffractometer using Cu K_α_ radiation (λ = 1.54184 Å) from a micro-focus X-ray source and an Oxford Instruments Cryojet XL cooler. The selected suitable single crystal was mounted using polybutene oil on a flexible loop fixed on a goniometer head and immediately transferred to the diffractometer. Pre-experiment, data collection, data reduction and analytical absorption correction^[5]^ were performed with the program suite *CrysAlisPro*.^[6]^ Using *Olex2*,^[7]^ the structure was solved with the SHELXT^[8]^ small molecule structure solution program and refined with the *SHELXL2018/3* program package^[9]^ by full-matrix least-squares minimization on F^2^. *PLATON*^[10]^ was used to check the result of the X-ray analysis. For more details about the data collection and refinement parameters, see the CIF file.

**Special features**

The isopropyl groups are disordered over two sets of positions with site-occupancy factors 0.500(4), 0.498(4) or 0.502(4).

| **Crystal data and structure refinement for 4** | |
| --- | --- |
| Empirical formula | C_34_H_32_Cl_2_O_2_Si |
| Formula weight | 571.58 |
| Temperature/K | 160(1) |
| Crystal system | triclinic |
| Space group | P-1 |
|  |  |
| a/Å | 7.0855(3) |
| b/Å | 20.3341(8) |
| c/Å | 20.7002(6) |
| α/° | 104.572(3) |
| β/° | 95.179(3) |
| γ/° | 93.589(3) |
| Volume/Å^3^ | 2863.42(19) |
| Z | 4 |
| ρ_calc_g/cm3 | 1.326 |
| μ/mm^-1^ | 2.673 |
| F(000) | 1200.0 |
| Crystal size/mm^3^ | 0.32 × 0.08 × 0.03 |
| Radiation | Cu Kα (λ = 1.54184) |
| 2Θ range for data collection/° | 4.438 to 149.016 |
| Index ranges | -8 ≤ h ≤ 8, -25 ≤ k ≤ 25, -25 ≤ l ≤ 25 |
| Reflections collected | 55067 |
| Independent reflections | 11673 [R_int_ = 0.0727, R_sigma_ = 0.0521] |
| Data/restraints/parameters | 11673/852/891 |
| Goodness-of-fit on F^2^ | 1.013 |
| Final R indexes [I>=2σ (I)] | R_1_ = 0.0805, wR_2_ = 0.2013 |
| Final R indexes [all data] | R_1_ = 0.1221, wR_2_ = 0.2304 |
| Largest diff. peak/hole / e Å^-3^ | 0.61/-0.85 |

**5**: (CCDC 2113700):


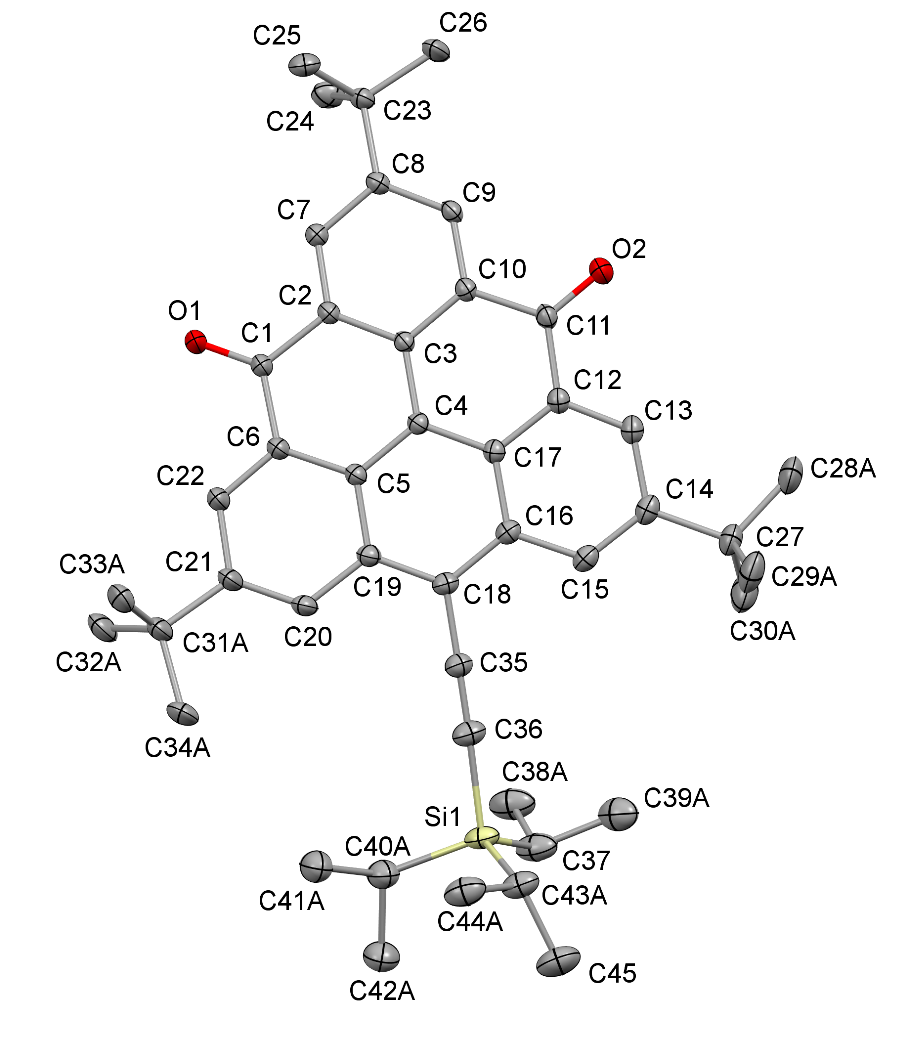


**Experimental**

Single-crystal X-ray diffraction data were collected at 160(1) K on a Rigaku OD SuperNova/Atlas area-detector diffractometer using Cu K_α_ radiation (λ = 1.54184 Å) from a micro-focus X-ray source and an Oxford Instruments Cryojet XL cooler. The selected suitable single crystal was mounted using polybutene oil on a flexible loop fixed on a goniometer head and immediately transferred to the diffractometer. Pre-experiment, data collection, data reduction and analytical absorption correction^[5]^ were performed with the program suite *CrysAlisPro*.^[6]^ Using *Olex2*,^[7]^ the structure was solved with the SHELXT^[8]^ small molecule structure solution program and refined with the *SHELXL2018/3* program package^[9]^ by full-matrix least-squares minimization on F^2^. *PLATON*^[10]^ was used to check the result of the X-ray analysis. For more details about the data collection and refinement parameters, see the CIF file.

**Special features**

Two tertiobutyl groups and all isopropyl groups are disordered over two sets of positions (site-occupancy factors in the CIF).

| **Crystal data and structure refinement for 5** | |
| --- | --- |
| Empirical formula | C_45_H_54_O_2_Si |
| Formula weight | 654.97 |
| Temperature/K | 160(1) |
| Crystal system | monoclinic |
| Space group | P2_1_/n |
| a/Å | 12.11580(10) |
| b/Å | 23.8084(3) |
| c/Å | 13.6183(2) |
| α/° | 90 |
| β/° | 101.3120(10) |
| γ/° | 90 |
| Volume/Å^3^ | 3851.99(8) |
| Z | 4 |
| ρ_calc_g/cm^3^ | 1.129 |
| μ/mm_­­_^-1^ | 0.795 |
| F(000) | 1416.0 |
| Crystal size/mm^3^ | 0.38 × 0.11 × 0.09 |
| Radiation | Cu Kα (λ = 1.54184) |
| 2Θ range for data collection/° | 7.426 to 149.002 |
| Index ranges | -14 ≤ h ≤ 15, -29 ≤ k ≤ 29, -16 ≤ l ≤ 17 |
| Reflections collected | 39210 |
| Independent reflections | 7871 [R_int_ = 0.0171, R_sigma_ = 0.0109] |
| Data/restraints/parameters | 7871/679/589 |
| Goodness-of-fit on F^2^ | 1.027 |
| Final R indexes [I>=2σ (I)] | R_1_ = 0.0745, wR_2_ = 0.1986 |
| Final R indexes [all data] | R_1_ = 0.0797, wR_2_ = 0.2039 |
| Largest diff. peak/hole / e Å^-3^ | 0.67/-0.72 |

**6**: (CCDC 2113699):


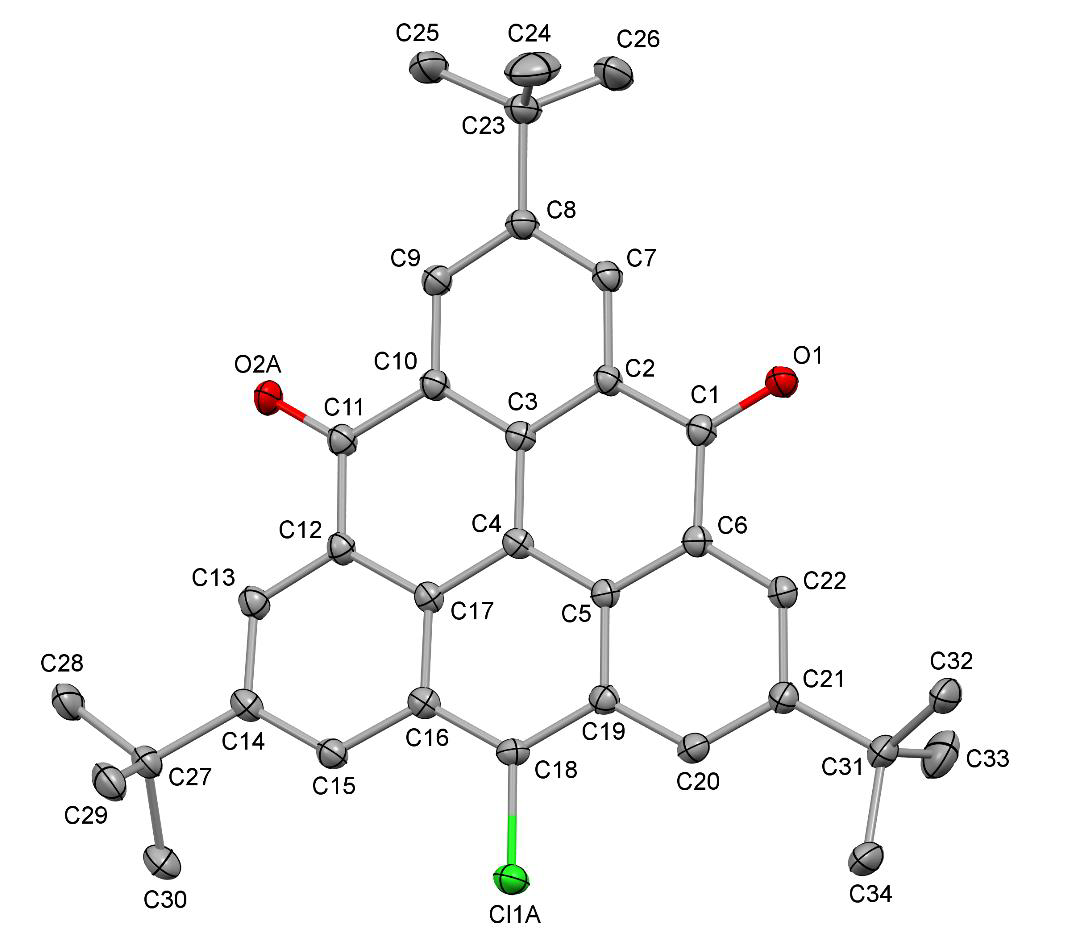


**Experimental**

Single-crystal X-ray diffraction data were collected at 160(1) K on a Rigaku OD SuperNova/Atlas area-detector diffractometer using Cu K_α_ radiation (λ = 1.54184 Å) from a micro-focus X-ray source and an Oxford Instruments Cryojet XL cooler. The selected suitable single crystal was mounted using polybutene oil on a flexible loop fixed on a goniometer head and immediately transferred to the diffractometer. Pre-experiment, data collection, data reduction and analytical absorption correction^[5]^ were performed with the program suite *CrysAlisPro*.^[6]^ Using *Olex2*,^[7]^ the structure was solved with the SHELXT^[8]^ small molecule structure solution program and refined with the *SHELXL2018/3* program package^[9]^ by full-matrix least-squares minimization on F^2^. *PLATON*^[10]^ was used to check the result of the X-ray analysis. For more details about the data collection and refinement parameters, see the CIF file.

**Special features**

The Cl atom and one O atom are disordered over two positions with site-occupancy factors of 0.194(3) and 0.806(3).

| **Crystal data and structure refinement for 6** | |
| --- | --- |
| Empirical formula | C_34_H_33_ClO_2_ |
| Formula weight | 509.05 |
| Temperature/K | 160(1) |
| Crystal system | triclinic |
| Space group | P-1 |
| a/Å | 7.5498(3) |
| b/Å | 14.0452(7) |
| c/Å | 14.6098(5) |
| α/° | 114.182(4) |
| β/° | 102.642(3) |
| γ/° | 96.380(4) |
| Volume/Å^3^ | 1343.46(11) |
| Z | 2 |
| ρ_calc_g/cm^3^ | 1.258 |
| μ/mm^-1^ | 1.478 |
| F(000) | 540.0 |
| Crystal size/mm^3^ | 0.27 × 0.05 × 0.02 |
| Radiation | Cu Kα (λ = 1.54184) |
| 2Θ range for data collection/° | 6.934 to 149 |
| Index ranges | -9 ≤ h ≤ 9, -17 ≤ k ≤ 17, -12 ≤ l ≤ 18 |
| Reflections collected | 24020 |
| Independent reflections | 5468 [R_int_ = 0.0465, R_sigma_ = 0.0360] |
| Data/restraints/parameters | 5468/14/362 |
| Goodness-of-fit on F^2^ | 1.035 |
| Final R indexes [I>=2σ (I)] | R_1_ = 0.0671, wR_2_ = 0.1910 |
| Final R indexes [all data] | R_1_ = 0.0899, wR_2_ = 0.2117 |
| Largest diff. peak/hole / e Å^-3^ | 0.66/-0.28 |

# Computational studies

The computational results are listed in the following section. First the excitation wavelength and oscillator strength obtained by TD-DFT are given for **1**, **2**, **SubPc-Ar**, **3a** (where the trimethylsilyl group is replaced by a hydrogen, *i.e.* terminal alkyne), **4**, **5**, **6** and **8a** (Table S1 - Table S16), followed by Natural transition orbitals calculated with CAM-B3LYP/6-31g(d,p) for **1** and **2** (Figure S77 - Figure S82). The third section contains tables with calculated electrochemical properties for **1**, **2**, **SubPc-Ar**, **3a**, **4**, **5**, **6** and **8a** (Table S17 and Table S18). The fourth section contains optimized geometries with the CAM-B3LYP/6-31+g(d,p) method. Any missing data in the computational section is due to convergence problems which was especially encountered for optimization of the charged species.

All geometries are optimized with DFT methods using the CAM-B3LYP^[12]^ and M06-2X^[13]^ functionals in connection with the 6-31g(d,p) and 6-31+g(d,p) basis sets^[14]^. All calculations are done with the IEF-PCM solvation model in Gaussian 16 revision A.03.^[15]^ Convergence issue was observed with diffuse functions which was circumvented by lowering the accuracy of the two electron integrals via the Gaussian keyword (int=acc2e=11); this lowering of accuracy leads to a change in the absolute Gibbs energy of less than 0.005 eV except for **5**  where the change in Gibbs energy is 0.019 eV and 0.024 eV for CAM-B3LYP/6-31g(d,p) and M06-2X/6-31g(d,p), respectively; such small change in Gibbs-free energies are expected to be insignificant for the overall accuracy of the methods. TD-DFT calculations were done to calculate electronic transitions, and natural transition orbitals (NTO) are calculated to visualize which orbitals are involved in the electronic transition. Generally, it is observed that calculated excitation wavelengths are significantly blue-shifted compared to experimental results; however, qualitative agreement was found in the relative position and intensity of the transition, which makes TD-DFT a powerful tool for analyzing electronic transitions. The HOMO energy level is determined directly from the orbital energy ε_SCF-HOMO_, while the LUMO energy level is calculated as ε_SCF-HOMO_ + TD-DFT(E_HOMO🡪LUMO_). Absolute redox potentials are calculated from equation (1):

$E_{abs(298.15K)}^{○}=\frac{\Delta G_{\mathrm{ox}}^{○}-\Delta G_{\mathrm{red}}^{○}}{F}+\frac{\Delta G_{\mathrm{elec}}^{○}}{F}$ (1)

Where, $F$ is Faradays constant, $\Delta G_{\mathrm{ox}}^{○}$ and $\Delta G_{\mathrm{red}}^{○}$ is the free energy of the redox couple, while $\Delta G_{\mathrm{elec}}^{○}$ is the Gibbs energy of a free resting electron in vacuum calculated from enthalpy and entropy contributions by Bartmess (-3.63kJ/mol)^[16]^ obtained from Fermi-Dirac statistical mechanics. The absolute redox potentials of ferrocene (4.8 eV) determined by Mikkelsen and co-workers^[17]^ is subtracted from the calculated absolute redox potentials to obtain E_ox,calc_/E_red,calc_ which can be directly compared to the experimental determined redox potentials.

**TD-DFT**

TD-DFT calculations of excitation wavelength (***λ*_calc_**) and oscillator strength (***f*_calc_**) for 40 lowest electronic transition are given in tables below. Excitation wavelengths below 245 nm are omitted from the tables.

Table S1: TD-DFT calculations of **1** CAM-B3LYP/6-31+g(d,p) and CAM-B3LYP/6-31g(d,p).

| Excited state | ***λ*_calc (nm)_ ^a^** | ***f*_calc_ ^a^** | ***λ*_calc (nm)_ ^b^** | ***f*_calc_ ^b^** |
| --- | --- | --- | --- | --- |
| 1 | 557.98 | 1.2949 | 546.26 | 1.2869 |
| 2 | 500.25 | 0.3980 | 490.41 | 0.3927 |
| 3 | 493.10 | 0.2769 | 481.54 | 0.2800 |
| 4 | 401.80 | 0.0132 | 392.46 | 0.0117 |
| 5 | 368.70 | 0.0588 | 354.46 | 0.0000 |
| 6 | 352.02 | 0.0000 | 351.96 | 0.0456 |
| 7 | 348.98 | 0.0000 | 350.56 | 0.0000 |
| 8 | 343.96 | 0.0961 | 337.74 | 0.0880 |
| 9 | 340.00 | 0.2053 | 335.74 | 0.1853 |
| 10 | 321.04 | 0.0121 | 318.45 | 0.0138 |
| 11 | 315.21 | 0.1628 | 311.41 | 0.2060 |
| 12 | 310.59 | 0.0127 | 307.98 | 0.0034 |
| 13 | 309.25 | 0.0491 | 304.75 | 0.0090 |
| 14 | 302.70 | 0.0183 | 298.40 | 0.0107 |
| 15 | 300.67 | 0.0541 | 296.50 | 0.0995 |
| 16 | 300.35 | 0.0946 | 295.22 | 0.0486 |
| 17 | 297.77 | 0.0044 | 292.26 | 0.0543 |
| 18 | 294.88 | 0.0820 | 289.28 | 0.0188 |
| 19 | 289.01 | 0.0022 | 279.70 | 0.1337 |
| 20 | 283.75 | 0.1053 | 279.27 | 0.0224 |
| 21 | 283.48 | 0.0099 | 276.67 | 0.0053 |
| 22 | 279.09 | 0.0072 | 276.38 | 0.0013 |
| 23 | 278.03 | 0.0002 | 272.94 | 0.0409 |
| 24 | 276.01 | 0.0803 | 269.76 | 0.0029 |
| 25 | 273.06 | 0.0073 | 268.28 | 0.0999 |
| 26 | 271.27 | 0.0101 | 267.38 | 0.0045 |
| 27 | 269.60 | 0.1407 | 264.34 | 0.3424 |
| 28 | 267.95 | 0.3642 | 263.58 | 0.1756 |
| 29 | 267.07 | 0.4641 | 263.14 | 0.4866 |
| 30 | 265.36 | 0.0654 | 262.48 | 0.0545 |
| 31 | 263.83 | 0.1548 | 261.58 | 0.0842 |
| 32 | 262.19 | 0.0794 | 260.62 | 0.0191 |
| 33 | 259.59 | 0.0181 | 256.95 | 0.1436 |
| 34 | 258.32 | 0.4134 | 255.90 | 0.2831 |
| 35 | 257.26 | 0.0655 | 254.50 | 0.1358 |
| 36 | 254.27 | 0.0150 | 252.93 | 0.0089 |
| 37 | 253.86 | 0.0141 | 251.99 | 0.0182 |
| 38 | 252.98 | 0.2344 | 251.60 | 0.0293 |
| 39 | 252.29 | 0.0344 | 249.88 | 0.0000 |
| 40 | 250.97 | 0.0919 | 248.98 | 0.1989 |

**^a^Calculations performed with CAM-B3LYP/6-31+g(d,p). ^b^Calculations performed with CAM-B3LYP/6-31g(d,p).**

Table S2: TD-DFT calculations of **1** M06-2X/6-31+g(d,p) and M06-2X/6-31g(d,p).

| Excited state | ***λ*_calc (nm)_ ^a^** | ***f*_calc_ ^a^** | ***λ*_calc (nm)_ ^b^** | ***f*_calc_ ^b^** |
| --- | --- | --- | --- | --- |
| 1 | 553.95 | 1.3087 | 542.64 | 1.3027 |
| 2 | 489.01 | 0.2041 | 479.60 | 0.3267 |
| 3 | 486.86 | 0.4479 | 476.24 | 0.3266 |
| 4 | 420.69 | 0.0409 | 411.33 | 0.0363 |
| 5 | 369.53 | 0.0555 | 365.89 | 0.0000 |
| 6 | 363.90 | 0.0000 | 362.98 | 0.0000 |
| 7 | 361.47 | 0.0000 | 353.81 | 0.0490 |
| 8 | 345.86 | 0.1604 | 341.70 | 0.1419 |
| 9 | 340.92 | 0.0868 | 335.38 | 0.0779 |
| 10 | 325.04 | 0.0044 | 322.58 | 0.0051 |
| 11 | 321.59 | 0.2021 | 318.62 | 0.1883 |
| 12 | 313.07 | 0.0139 | 309.88 | 0.0040 |
| 13 | 312.09 | 0.0014 | 308.40 | 0.0344 |
| 14 | 304.45 | 0.0084 | 298.89 | 0.0412 |
| 15 | 303.46 | 0.0554 | 295.42 | 0.0266 |
| 16 | 301.76 | 0.0240 | 294.48 | 0.0945 |
| 17 | 298.14 | 0.0411 | 293.07 | 0.0030 |
| 18 | 296.80 | 0.0578 | 290.07 | 0.0346 |
| 19 | 293.75 | 0.0429 | 289.77 | 0.0038 |
| 20 | 285.03 | 0.0687 | 283.33 | 0.0001 |
| 21 | 284.31 | 0.0403 | 280.56 | 0.1426 |
| 22 | 283.04 | 0.0063 | 279.63 | 0.0033 |
| 23 | 282.98 | 0.0020 | 278.70 | 0.0077 |
| 24 | 280.93 | 0.0046 | 274.30 | 0.0246 |
| 25 | 277.74 | 0.0569 | 270.99 | 0.0232 |
| 26 | 274.56 | 0.0323 | 269.50 | 0.0157 |
| 27 | 272.04 | 0.0141 | 268.77 | 0.0583 |
| 28 | 270.17 | 0.2040 | 265.53 | 0.2106 |
| 29 | 268.82 | 0.3122 | 264.31 | 0.2272 |
| 30 | 267.47 | 0.3891 | 264.00 | 0.1846 |
| 31 | 265.45 | 0.3114 | 263.28 | 0.5468 |
| 32 | 264.27 | 0.0063 | 262.23 | 0.0548 |
| 33 | 262.64 | 0.0019 | 259.26 | 0.0311 |
| 34 | 261.95 | 0.0613 | 258.23 | 0.0891 |
| 35 | 261.26 | 0.0761 | 256.47 | 0.4085 |
| 36 | 259.13 | 0.3986 | 252.76 | 0.0216 |
| 37 | 255.03 | 0.1121 | 252.39 | 0.1264 |
| 38 | 253.75 | 0.1455 | 250.07 | 0.1317 |
| 39 | 253.53 | 0.0329 | 249.92 | 0.0144 |
| 40 | 251.62 | 0.0188 | 248.25 | 0.0042 |

**^a^Calculations performed with M06-2X/6-31+g(d,p). ^b^Calculations performed with M06-2X/6-31g(d,p).**

Table S3: TD-DFT calculations of **2** CAM-B3LYP/6-31+g(d,p) and CAM-B3LYP/6-31g(d,p).

| Excited state | ***λ*_calc (nm)_ ^a^** | ***f*_calc_ ^a^** | ***λ*_calc (nm)_ ^b^** | ***f*_calc_ ^b^** |
| --- | --- | --- | --- | --- |
| 1 | 512.45 | 0.4028 | 502.16 | 0.3902 |
| 2 | 506.50 | 0.4425 | 497.04 | 0.4390 |
| 3 | 477.37 | 0.3991 | 463.67 | 0.3930 |
| 4 | 415.24 | 0.0001 | 401.45 | 0.0022 |
| 5 | 395.23 | 0.0053 | 394.62 | 0.0077 |
| 6 | 390.50 | 0.0424 | 386.06 | 0.0095 |
| 7 | 381.50 | 0.0178 | 366.48 | 0.0442 |
| 8 | 342.07 | 0.0291 | 343.32 | 0.0001 |
| 9 | 341.08 | 0.0369 | 341.60 | 0.0000 |
| 10 | 340.44 | 0.0099 | 334.43 | 0.0681 |
| 11 | 311.96 | 0.0062 | 300.01 | 0.0000 |
| 12 | 302.99 | 0.0001 | 298.11 | 0.0102 |
| 13 | 302.06 | 0.0246 | 294.77 | 0.0096 |
| 14 | 295.50 | 0.0195 | 292.34 | 0.0177 |
| 15 | 295.41 | 0.0387 | 291.86 | 0.0261 |
| 16 | 290.12 | 0.0025 | 283.66 | 0.0008 |
| 17 | 279.57 | 0.0008 | 272.94 | 0.0211 |
| 18 | 279.34 | 0.0037 | 272.86 | 0.0176 |
| 19 | 278.84 | 0.0251 | 272.61 | 0.0132 |
| 20 | 278.55 | 0.0095 | 271.29 | 0.0010 |
| 21 | 274.13 | 0.0004 | 268.76 | 0.0007 |
| 22 | 270.66 | 0.0652 | 266.85 | 0.0585 |
| 23 | 268.58 | 0.0128 | 266.29 | 0.0576 |
| 24 | 267.45 | 0.6154 | 263.90 | 0.4735 |
| 25 | 264.94 | 0.5758 | 262.79 | 0.1226 |
| 26 | 264.83 | 0.4942 | 261.38 | 0.7488 |
| 27 | 262.52 | 0.0678 | 260.52 | 0.0375 |
| 28 | 261.57 | 0.2172 | 259.38 | 0.2357 |
| 29 | 261.44 | 0.3295 | 258.44 | 0.6851 |
| 30 | 259.41 | 0.0011 | 254.00 | 0.0213 |
| 31 | 258.39 | 0.0050 | 253.93 | 0.0072 |
| 32 | 255.23 | 0.0002 | 252.97 | 0.1143 |
| 33 | 254.51 | 0.1128 | 251.43 | 0.0010 |
| 34 | 251.73 | 0.0105 | 251.04 | 0.0005 |
| 35 | 251.46 | 0.0018 | 250.55 | 0.0065 |
| 36 | 250.36 | 0.8014 |  |  |
| 37 | 249.63 | 1.1247 |  |  |
| 38 | 246.28 | 0.0001 |  |  |
| 39 |  |  |  |  |
| 40 |  |  |  |  |

**^a^Calculations performed with CAM-B3LYP/6-31+g(d,p). ^b^Calculations performed with CAM-B3LYP/6-31g(d,p).**

Table S4: TD-DFT calculations of **2** M06-2X/6-31+g(d,p) and M06-2X/6-31g(d,p).

| Excited state | ***λ*_calc (nm)_ ^a^** | ***f*_calc_ ^a^** | ***λ*_calc (nm)_ ^b^** | ***f*_calc_ ^b^** |
| --- | --- | --- | --- | --- |
| 1 | 502.40 | 0.4100 | 492.55 | 0.4002 |
| 2 | 493.14 | 0.4829 | 484.20 | 0.4711 |
| 3 | 474.70 | 0.3591 | 460.51 | 0.3687 |
| 4 | 438.80 | 0.0007 | 424.07 | 0.0007 |
| 5 | 411.41 | 0.0062 | 407.80 | 0.0079 |
| 6 | 401.11 | 0.0220 | 397.00 | 0.0126 |
| 7 | 382.40 | 0.0261 | 366.37 | 0.0333 |
| 8 | 355.58 | 0.0000 | 356.75 | 0.0002 |
| 9 | 354.66 | 0.0001 | 355.58 | 0.0001 |
| 10 | 338.22 | 0.0700 | 331.93 | 0.0637 |
| 11 | 328.29 | 0.0043 | 313.00 | 0.0040 |
| 12 | 301.68 | 0.0000 | 298.41 | 0.0000 |
| 13 | 301.18 | 0.0144 | 294.73 | 0.0086 |
| 14 | 293.59 | 0.0324 | 289.97 | 0.0223 |
| 15 | 293.28 | 0.0155 | 289.86 | 0.0126 |
| 16 | 288.33 | 0.0009 | 282.46 | 0.0049 |
| 17 | 282.40 | 0.0027 | 274.83 | 0.0118 |
| 18 | 280.41 | 0.0285 | 273.71 | 0.0421 |
| 19 | 280.09 | 0.0057 | 272.40 | 0.0045 |
| 20 | 278.76 | 0.0003 | 271.92 | 0.0008 |
| 21 | 275.85 | 0.0009 | 271.82 | 0.0006 |
| 22 | 274.44 | 0.0027 | 270.22 | 0.0001 |
| 23 | 272.12 | 0.2456 | 268.28 | 0.0314 |
| 24 | 271.04 | 0.0494 | 267.03 | 0.1028 |
| 25 | 267.27 | 0.6208 | 264.72 | 0.4503 |
| 26 | 264.83 | 0.1105 | 263.01 | 0.1722 |
| 27 | 264.74 | 0.5121 | 261.89 | 0.4781 |
| 28 | 262.59 | 0.1958 | 260.25 | 0.2408 |
| 29 | 261.93 | 0.3212 | 258.96 | 0.6617 |
| 30 | 260.07 | 0.0074 | 254.59 | 0.1074 |
| 31 | 259.58 | 0.0000 | 254.15 | 0.0569 |
| 32 | 258.43 | 0.0874 | 252.53 | 0.0001 |
| 33 | 256.53 | 0.0610 | 251.30 | 0.0049 |
| 34 | 254.53 | 0.0149 | 250.94 | 0.0003 |
| 35 | 252.44 | 0.0007 | 250.38 | 0.0053 |
| 36 | 252.23 | 1.0071 | 246.82 | 0.0671 |
| 37 | 251.51 | 0.3224 | 246.61 | 0.8022 |
| 38 | 251.01 | 0.0841 | 246.27 | 0.4015 |
| 39 | 250.32 | 0.0586 | 245.24 | 0.0218 |
| 40 | 249.64 | 0.0534 |  |  |

**^a^Calculations performed with M06-2X/6-31+g(d,p). ^b^Calculations performed with M06-2X/6-31g(d,p).**

Table S5: TD-DFT calculations of **SubPC-Ar** CAM-B3LYP/6-31+g(d,p) and CAM-B3LYP/6-31g(d,p).

| Excited state | ***λ*_calc (nm)_ ^a^** | ***f*_calc_ ^a^** | ***λ*_calc (nm)_ ^b^** | ***f*_calc_ ^b^** |
| --- | --- | --- | --- | --- |
| 1 | 505.55 | 0.4565 | 495.25 | 0.4393 |
| 2 | 504.64 | 0.4852 | 494.27 | 0.4668 |
| 3 | 310.57 | 0.0074 | 307.63 | 0.0087 |
| 4 | 307.54 | 0.0114 | 304.88 | 0.0120 |
| 5 | 301.60 | 0.0005 | 298.29 | 0.0004 |
| 6 | 294.59 | 0.0308 | 290.73 | 0.0231 |
| 7 | 294.37 | 0.0400 | 290.55 | 0.0310 |
| 8 | 280.39 | 0.0007 | 272.95 | 0.0340 |
| 9 | 278.96 | 0.0252 | 272.88 | 0.0269 |
| 10 | 278.89 | 0.0187 | 269.48 | 0.0001 |
| 11 | 271.73 | 0.0007 | 269.00 | 0.0027 |
| 12 | 264.48 | 0.7751 | 261.59 | 0.6899 |
| 13 | 264.18 | 0.6792 | 261.35 | 0.5673 |
| 14 | 262.10 | 0.0469 | 260.96 | 0.0369 |
| 15 | 260.16 | 0.2600 | 258.04 | 0.2822 |
| 16 | 260.10 | 0.2502 | 257.96 | 0.3628 |
| 17 | 257.21 | 0.0027 | 253.70 | 0.0013 |
| 18 | 255.04 | 0.0419 | 252.85 | 0.0694 |
| 19 | 253.05 | 0.0010 | 252.20 | 0.0048 |
| 20 | 251.89 | 0.0539 | 250.30 | 0.0057 |
| 21 | 251.16 | 0.0011 | 250.27 | 0.0105 |
| 22 | 250.71 | 0.0036 | 248.41 | 0.0383 |
| 23 | 250.66 | 0.0022 |  |  |

**^a^Calculations performed with CAM-B3LYP/6-31+g(d,p). ^b^Calculations performed with CAM-B3LYP/6-31g(d,p).**

Table S6: TD-DFT calculations of **SubPC-Ar** M06-2X/6-31+g(d,p) and M06-2X/6-31g(d,p).

| Excited state | ***λ*_calc (nm)_ ^a^** | ***f*_calc_ ^a^** | ***λ*_calc (nm)_ ^b^** | ***f*_calc_ ^b^** |
| --- | --- | --- | --- | --- |
| 1 | 494.04 | 0.4649 | 484.30 | 0.4505 |
| 2 | 491.64 | 0.5038 | 482.28 | 0.4861 |
| 3 | 312.13 | 0.0054 | 309.24 | 0.0058 |
| 4 | 308.60 | 0.0105 | 306.25 | 0.0110 |
| 5 | 298.14 | 0.0000 | 294.95 | 0.0000 |
| 6 | 291.93 | 0.0297 | 288.01 | 0.0211 |
| 7 | 291.79 | 0.0352 | 287.68 | 0.0265 |
| 8 | 282.78 | 0.0007 | 273.55 | 0.0227 |
| 9 | 279.70 | 0.0107 | 273.33 | 0.0357 |
| 10 | 279.56 | 0.0225 | 270.95 | 0.0001 |
| 11 | 270.96 | 0.0009 | 269.00 | 0.0015 |
| 12 | 264.98 | 0.0024 | 262.84 | 0.0091 |
| 13 | 264.38 | 0.1397 | 261.43 | 0.5966 |
| 14 | 264.14 | 0.5687 | 261.02 | 0.3187 |
| 15 | 263.62 | 0.2877 | 259.09 | 0.1695 |
| 16 | 262.30 | 0.3221 | 258.19 | 0.3007 |
| 17 | 260.26 | 0.2493 | 257.37 | 0.4081 |
| 18 | 259.57 | 0.3085 | 255.85 | 0.0667 |
| 19 | 258.24 | 0.0276 | 250.53 | 0.0893 |
| 20 | 253.53 | 0.0959 | 249.71 | 0.0010 |
| 21 | 251.31 | 0.0003 | 249.39 | 0.0006 |
| 22 | 250.60 | 0.0023 | 249.20 | 0.0063 |
| 23 | 250.49 | 0.0025 |  |  |

**^a^Calculations performed with M06-2X/6-31+g(d,p). ^b^Calculations performed with M06-2X/6-31g(d,p).**

Table S7: TD-DFT calculations of **3a** CAM-B3LYP/6-31+g(d,p) and CAM-B3LYP/6-31g(d,p).

| Excited state | ***λ*_calc (nm)_ ^a^** | ***f*_calc_ ^a^** | ***λ*_calc (nm)_ ^b^** | ***f*_calc_ ^b^** |
| --- | --- | --- | --- | --- |
| 1 | 478.59 | 0.4474 | 465.22 | 0.4361 |
| 2 | 358.33 | 0.0479 | 354.15 | 0.0000 |
| 3 | 352.20 | 0.0000 | 350.40 | 0.0000 |
| 4 | 349.17 | 0.0000 | 343.40 | 0.0328 |
| 5 | 343.06 | 0.1117 | 336.25 | 0.1042 |
| 6 | 306.83 | 0.0074 | 300.47 | 0.0042 |
| 7 | 298.40 | 0.0910 | 292.49 | 0.0721 |
| 8 | 282.85 | 0.0051 | 277.51 | 0.0065 |
| 9 | 266.76 | 0.1979 | 263.50 | 0.0000 |
| 10 | 265.39 | 0.0000 | 260.59 | 0.2564 |
| 11 | 265.10 | 0.0236 | 259.55 | 0.0141 |
| 12 | 256.44 | 0.0010 | 249.65 | 0.0016 |
| 13 | 249.95 | 0.2411 | 248.51 | 0.0000 |
| 14 | 248.77 | 0.0000 | 245.77 | 0.0000 |
| 15 | 246.18 | 0.0000 |  |  |
| 16 | 246.08 | 1.3570 |  |  |

**^a^Calculations performed with CAM-B3LYP/6-31+g(d,p). ^b^Calculations performed with CAM-B3LYP/6-31g(d,p).**

Table S8: TD-DFT calculations of **3a** M06-2X/6-31+g(d,p) and M06-2X/6-31g(d,p).

| Excited state | ***λ*_calc (nm)_ ^a^** | ***f*_calc_ ^a^** | ***λ*_calc (nm)_ ^b^** | ***f*_calc_ ^b^** |
| --- | --- | --- | --- | --- |
| 1 | 475.51 | 0.4356 | 462.89 | 0.4279 |
| 2 | 363.88 | 0.0000 | 365.51 | 0.0000 |
| 3 | 361.51 | 0.0000 | 362.75 | 0.0000 |
| 4 | 358.73 | 0.0503 | 344.63 | 0.0450 |
| 5 | 339.50 | 0.0973 | 333.51 | 0.0860 |
| 6 | 304.64 | 0.0027 | 298.93 | 0.0013 |
| 7 | 296.81 | 0.0679 | 291.56 | 0.0581 |
| 8 | 281.78 | 0.0023 | 276.89 | 0.0035 |
| 9 | 272.37 | 0.0000 | 270.74 | 0.0000 |
| 10 | 266.87 | 0.3026 | 260.91 | 0.3490 |
| 11 | 263.69 | 0.0162 | 258.79 | 0.0111 |
| 12 | 256.58 | 0.0330 | 249.56 | 0.0234 |
| 13 | 247.90 | 0.2052 | 245.88 | 0.0000 |
| 14 | 246.94 | 0.0001 |  |  |
| 15 | 246.69 | 1.2617 |  |  |

**^a^Calculations performed with M06-2X/6-31+g(d,p). ^b^Calculations performed with M06-2X/6-31g(d,p).**

Table S9: TD-DFT calculations of **4** CAM-B3LYP/6-31+g(d,p) and CAM-B3LYP/6-31g(d,p).

| Excited state | ***λ*_calc (nm)_ ^a^** | ***f*_calc_ ^a^** | ***λ*_calc (nm)_ ^b^** | ***f*_calc_ ^b^** |
| --- | --- | --- | --- | --- |
| 1 | 490.02 | 0.5841 | 477.42 | 0.5884 |
| 2 | 363.72 | 0.0560 | 353.98 | 0.0000 |
| 3 | 351.39 | 0.0000 | 350.23 | 0.0000 |
| 4 | 348.51 | 0.0000 | 347.13 | 0.0400 |
| 5 | 342.78 | 0.1007 | 336.72 | 0.0955 |
| 6 | 306.74 | 0.0040 | 301.19 | 0.0016 |
| 7 | 298.14 | 0.0718 | 293.16 | 0.0497 |
| 8 | 296.98 | 0.0011 | 292.23 | 0.0107 |
| 9 | 284.98 | 0.0671 | 279.00 | 0.0651 |
| 10 | 282.29 | 0.0065 | 277.60 | 0.0083 |
| 11 | 268.02 | 0.2107 | 261.64 | 0.2512 |
| 12 | 260.61 | 0.0390 | 253.94 | 0.0425 |
| 13 | 251.59 | 0.2351 | 248.81 | 0.0000 |
| 14 | 249.14 | 0.0065 | 246.14 | 0.0000 |
| 15 | 248.74 | 0.0000 |  |  |
| 16 | 246.98 | 1.2035 |  |  |
| 17 | 246.31 | 0.0001 |  |  |

**^a^Calculations performed with CAM-B3LYP/6-31+g(d,p). ^b^Calculations performed with CAM-B3LYP/6-31g(d,p).**

Table S10: TD-DFT calculations of **4** M06-2X/6-31+g(d,p) and M06-2X/6-31g(d,p).

| Excited state | ***λ*_calc (nm)_ ^a^** | ***f*_calc_ ^a^** | ***λ*_calc (nm)_ ^b^** | ***f*_calc_ ^b^** |
| --- | --- | --- | --- | --- |
| 1 | 487.60 | 0.5754 | 475.53 | 0.5826 |
| 2 | 364.02 | 0.0545 | 365.48 | 0.0000 |
| 3 | 363.34 | 0.0003 | 362.70 | 0.0000 |
| 4 | 361.07 | 0.0000 | 348.49 | 0.0475 |
| 5 | 339.56 | 0.0891 | 334.14 | 0.0814 |
| 6 | 306.93 | 0.0006 | 302.88 | 0.0004 |
| 7 | 305.27 | 0.0002 | 300.16 | 0.0000 |
| 8 | 296.83 | 0.0529 | 292.24 | 0.0482 |
| 9 | 289.18 | 0.0595 | 283.50 | 0.0594 |
| 10 | 281.50 | 0.0033 | 277.19 | 0.0051 |
| 11 | 268.29 | 0.3087 | 262.04 | 0.3387 |
| 12 | 262.10 | 0.0806 | 254.86 | 0.0748 |
| 13 | 255.31 | 0.0015 | 250.69 | 0.0016 |
| 14 | 253.52 | 0.0439 | 247.29 | 0.0201 |
| 15 | 249.16 | 0.1256 | 246.32 | 0.0019 |
| 16 | 247.59 | 1.1050 |  |  |
| 17 | 247.14 | 0.0001 |  |  |

**^a^Calculations performed with M06-2X/6-31+g(d,p). ^b^Calculations performed with M06-2X/6-31g(d,p).**

Table S11: TD-DFT calculations of **5** CAM-B3LYP/6-31+g(d,p) and CAM-B3LYP/6-31g(d,p).

| Excited state | ***λ*_calc (nm)_ ^a^** | ***f*_calc_ ^a^** | ***λ*_calc (nm)_ ^b^** | ***f*_calc_ ^b^** |
| --- | --- | --- | --- | --- |
| 1 | 508.59 | 0.5764 | 495.57 | 0.5872 |
| 2 | 372.47 | 0.0509 | 355.09 | 0.0428 |
| 3 | 357.20 | 0.1337 | 353.83 | 0.0002 |
| 4 | 351.03 | 0.0000 | 350.42 | 0.1275 |
| 5 | 347.53 | 0.0000 | 349.49 | 0.0000 |
| 6 | 323.34 | 0.0015 | 317.37 | 0.0012 |
| 7 | 303.23 | 0.0848 | 298.39 | 0.0621 |
| 8 | 293.66 | 0.0005 | 289.81 | 0.0006 |
| 9 | 287.32 | 0.0111 | 282.00 | 0.0128 |
| 10 | 285.07 | 0.0382 | 279.66 | 0.0356 |
| 11 | 272.71 | 0.0954 | 266.75 | 0.1742 |
| 12 | 264.97 | 0.0265 | 258.58 | 0.0405 |
| 13 | 255.57 | 0.1620 | 249.36 | 0.0002 |
| 14 | 253.64 | 0.7641 | 248.25 | 1.2870 |
| 15 | 253.00 | 0.9627 | 247.63 | 0.2029 |
| 16 | 249.19 | 0.0001 | 246.48 | 0.2018 |
| 17 | 245.78 | 0.0000 | 245.80 | 0.0000 |

**^a^Calculations performed with CAM-B3LYP/6-31+g(d,p). ^b^Calculations performed with CAM-B3LYP/6-31g(d,p).**

Table S12: TD-DFT calculations of **5** M06-2X/6-31+g(d,p) and M06-2X/6-31g(d,p).

| Excited state | ***λ*_calc (nm)_ ^a^** | ***f*_calc_ ^a^** | ***λ*_calc (nm)_ ^b^** | ***f*_calc_ ^b^** |
| --- | --- | --- | --- | --- |
| 1 | 508.57 | 0.5621 | 495.05 | 0.5769 |
| 2 | 374.37 | 0.0481 | 366.05 | 0.0000 |
| 3 | 363.99 | 0.0000 | 362.70 | 0.0000 |
| 4 | 361.11 | 0.0000 | 357.70 | 0.0483 |
| 5 | 354.32 | 0.1281 | 348.07 | 0.1173 |
| 6 | 323.25 | 0.0014 | 317.51 | 0.0028 |
| 7 | 304.23 | 0.0007 | 300.77 | 0.0007 |
| 8 | 301.72 | 0.0576 | 297.19 | 0.0441 |
| 9 | 289.48 | 0.0358 | 284.05 | 0.0354 |
| 10 | 286.50 | 0.0084 | 281.53 | 0.0097 |
| 11 | 273.68 | 0.1671 | 267.63 | 0.2594 |
| 12 | 267.07 | 0.0791 | 259.88 | 0.0877 |
| 13 | 256.87 | 0.1254 | 249.93 | 1.3663 |
| 14 | 255.54 | 1.4581 | 249.43 | 0.0828 |
| 15 | 252.92 | 0.0033 | 249.09 | 0.0034 |
| 16 | 251.52 | 0.0914 | 248.41 | 0.0036 |
| 17 | 249.54 | 0.0034 | 246.21 | 0.0313 |
| 18 | 248.94 | 0.1284 |  |  |
| 19 |  |  |  |  |
| 20 |  |  |  |  |

**^a^Calculations performed with M06-2X/6-31+g(d,p). ^b^Calculations performed with M06-2X/6-31g(d,p).**

Table S13: TD-DFT calculations of **6** CAM-B3LYP/6-31+g(d,p) and CAM-B3LYP/6-31g(d,p).

| Excited state | ***λ*_calc (nm)_ ^a^** | ***f*_calc_ ^a^** | ***λ*_calc (nm)_ ^b^** | ***f*_calc_ ^b^** |
| --- | --- | --- | --- | --- |
| 1 | 479.86 | 0.3636 | 466.11 | 0.3615 |
| 2 | 364.48 | 0.0416 | 352.68 | 0.0000 |
| 3 | 350.80 | 0.1246 | 348.66 | 0.0000 |
| 4 | 350.18 | 0.0000 | 347.62 | 0.0278 |
| 5 | 346.95 | 0.0000 | 343.97 | 0.1226 |
| 6 | 317.51 | 0.0066 | 311.29 | 0.0027 |
| 7 | 297.75 | 0.0849 | 292.56 | 0.0587 |
| 8 | 285.41 | 0.0051 | 279.56 | 0.0050 |
| 9 | 270.05 | 0.1905 | 264.28 | 0.2841 |
| 10 | 258.34 | 0.0701 | 250.49 | 0.0333 |
| 11 | 254.21 | 0.0465 | 247.86 | 0.0326 |
| 12 | 251.42 | 1.6903 | 246.63 | 0.0000 |
| 13 | 246.72 | 0.0002 | 246.31 | 1.5860 |
| 14 | 246.38 | 0.4238 |  |  |

**^a^Calculations performed with CAM-B3LYP/6-31+g(d,p). ^b^Calculations performed with CAM-B3LYP/6-31g(d,p).**

Table S14: TD-DFT calculations of **6** M06-2X/6-31+g(d,p) and M06-2X/6-31g(d,p).

| Excited state | ***λ*_calc (nm)_ ^a^** | ***f*_calc_ ^a^** | ***λ*_calc (nm)_ ^b^** | ***f*_calc_ ^b^** |
| --- | --- | --- | --- | --- |
| 1 | 480.88 | 0.3510 | 467.30 | 0.3515 |
| 2 | 367.03 | 0.0414 | 364.75 | 0.0000 |
| 3 | 362.74 | 0.0000 | 361.67 | 0.0000 |
| 4 | 360.14 | 0.0000 | 350.73 | 0.0394 |
| 5 | 347.96 | 0.1152 | 341.84 | 0.1051 |
| 6 | 316.54 | 0.0028 | 310.89 | 0.0011 |
| 7 | 296.27 | 0.0469 | 291.67 | 0.0337 |
| 8 | 284.12 | 0.0042 | 278.73 | 0.0048 |
| 9 | 271.31 | 0.2821 | 265.63 | 0.3792 |
| 10 | 257.98 | 0.0290 | 250.91 | 0.0029 |
| 11 | 253.79 | 1.5711 | 248.30 | 1.5072 |
| 12 | 252.98 | 0.0857 | 247.20 | 0.0795 |
| 13 | 246.99 | 0.5239 | 245.58 | 0.0000 |
| 14 | 246.63 | 0.0003 |  |  |

**^a^Calculations performed with M06-2X/6-31+g(d,p). ^b^Calculations performed with M06-2X/6-31g(d,p).**

Table S15: TD-DFT calculations of **8a** CAM-B3LYP/6-31+g(d,p) and CAM-B3LYP/6-31g(d,p).

| Excited state | ***λ*_calc (nm)_ ^a^** | ***f*_calc_ ^a^** | ***λ*_calc (nm)_ ^b^** | ***f*_calc_ ^b^** |
| --- | --- | --- | --- | --- |
| 1 | 500.72 | 0.3041 | 484.06 | 0.3038 |
| 2 | 397.11 | 0.0810 | 379.98 | 0.0727 |
| 3 | 342.49 | 0.0000 | 343.44 | 0.0000 |
| 4 | 341.57 | 0.0000 | 342.32 | 0.0000 |
| 5 | 339.59 | 0.0569 | 331.64 | 0.0402 |
| 6 | 305.34 | 0.0226 | 297.27 | 0.0116 |
| 7 | 293.46 | 0.0011 | 286.88 | 0.0200 |
| 8 | 281.24 | 0.0132 | 274.61 | 0.0152 |
| 9 | 269.14 | 0.7420 | 263.27 | 0.7929 |
| 10 | 265.17 | 0.0711 | 261.07 | 0.1412 |
| 11 | 255.28 | 0.1324 | 247.50 | 0.3588 |
| 12 | 252.36 | 1.0878 | 246.28 | 0.7523 |
| 13 | 251.64 | 0.5653 |  |  |

**^a^Calculations performed with CAM-B3LYP/6-31+g(d,p). ^b^Calculations performed with CAM-B3LYP/6-31g(d,p).**

Table S16: TD-DFT calculations of **8a** M06-2X/6-31+g(d,p) and M06-2X/6-31g(d,p).

| Excited state | ***λ*_calc (nm)_ ^a^** | ***f*_calc_ ^a^** | ***λ*_calc (nm)_ ^b^** | ***f*_calc_ ^b^** |
| --- | --- | --- | --- | --- |
| 1 | 504.35 | 0.2936 | 487.67 | 0.2961 |
| 2 | 400.83 | 0.0704 | 383.73 | 0.0663 |
| 3 | 355.01 | 0.0000 | 355.82 | 0.0000 |
| 4 | 354.09 | 0.0000 | 355.04 | 0.0000 |
| 5 | 337.19 | 0.0473 | 330.07 | 0.0332 |
| 6 | 303.90 | 0.0130 | 296.57 | 0.0062 |
| 7 | 292.09 | 0.0303 | 286.00 | 0.0622 |
| 8 | 280.37 | 0.0180 | 274.11 | 0.0332 |
| 9 | 269.75 | 0.7757 | 263.94 | 0.5479 |
| 10 | 268.08 | 0.1455 | 263.40 | 0.4064 |
| 11 | 255.05 | 0.4266 | 248.48 | 0.9840 |
| 12 | 253.83 | 0.8294 | 246.64 | 0.1513 |
| 13 | 249.29 | 0.4064 |  |  |
| 14 | 246.65 | 0.0011 |  |  |

**^a^Calculations performed with M06-2X/6-31+g(d,p). ^b^Calculations performed with M06-2X/6-31g(d,p).**

**Natural transition orbital (NTO) calculated with M06-2X/6-31g(d,p)**

NTO with coefficients higher than 0.30 are shown.

0.85


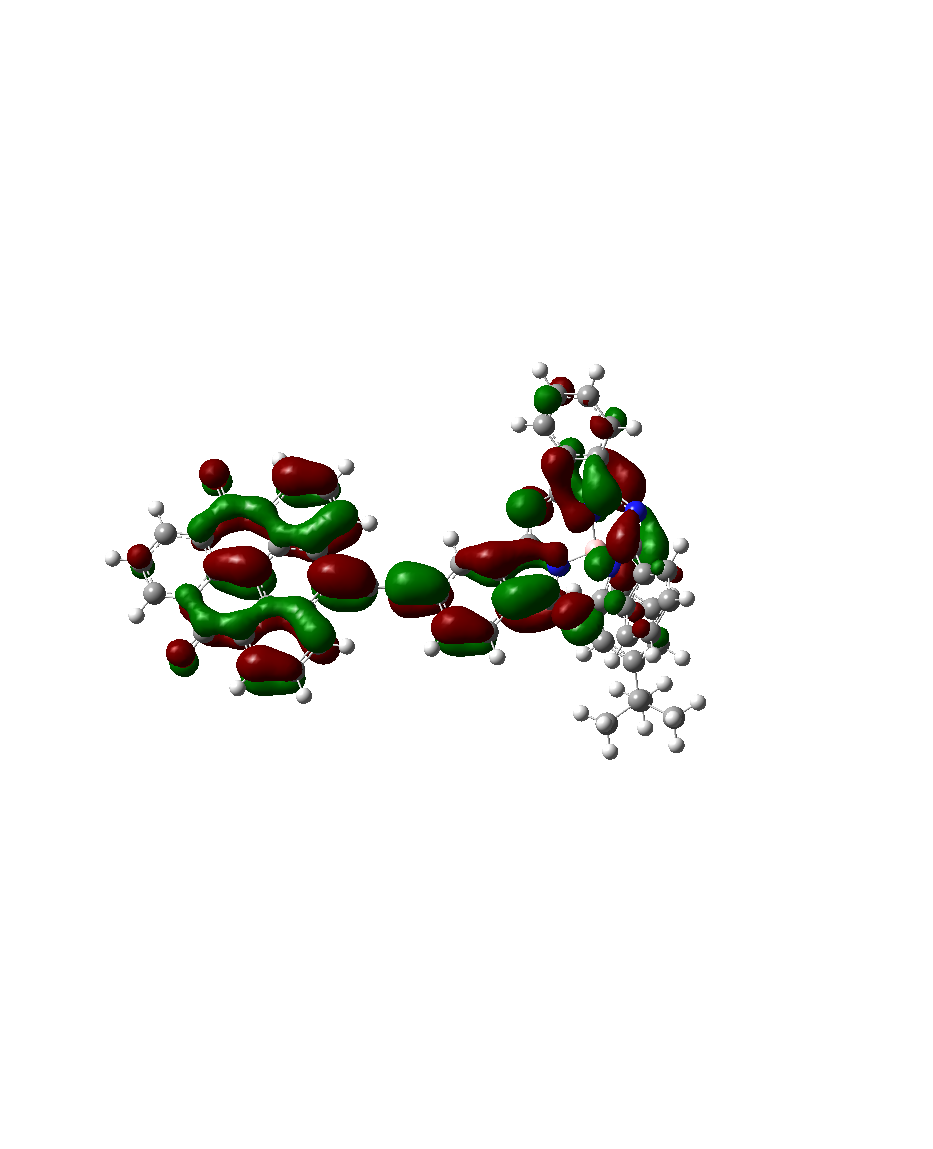

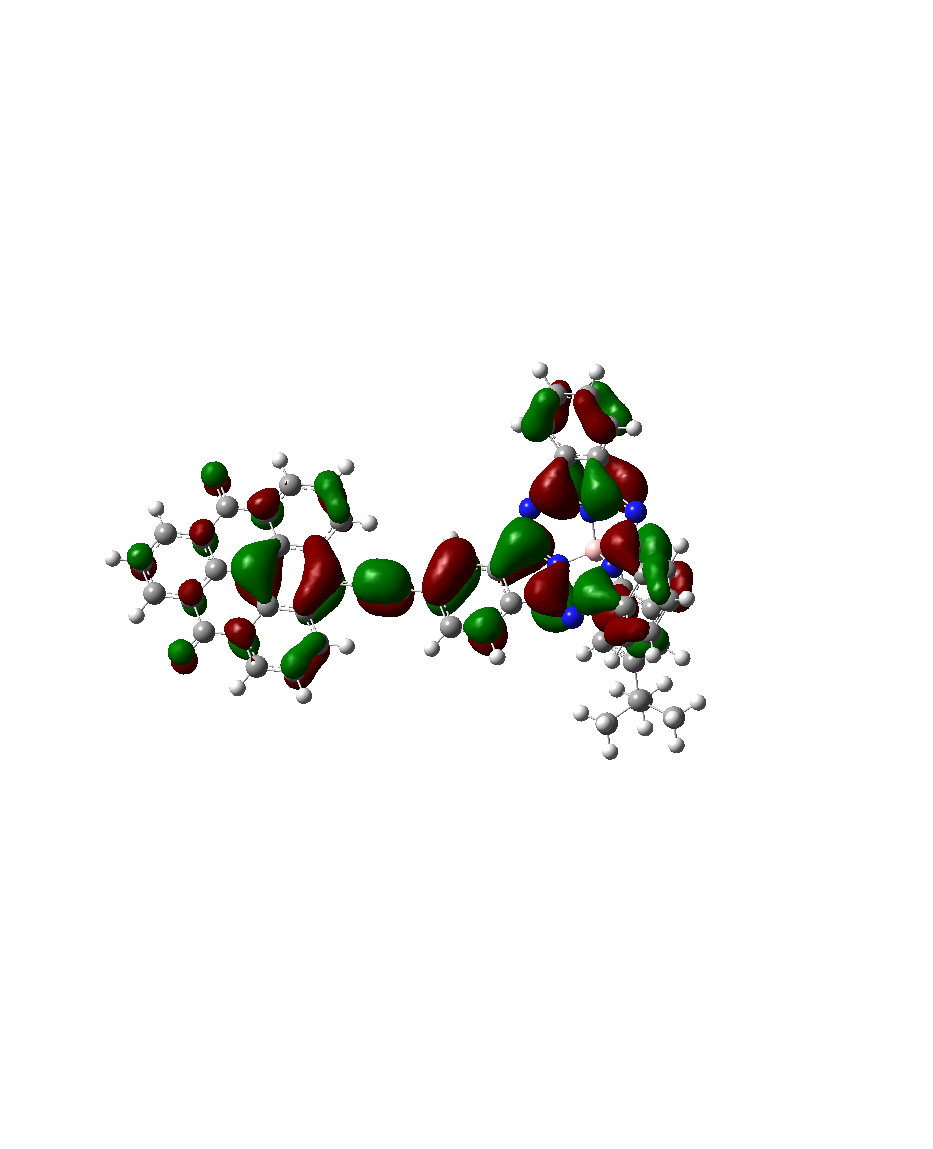


Figure S79: NTO for first excitation (543 nm) of **1**.

0.95


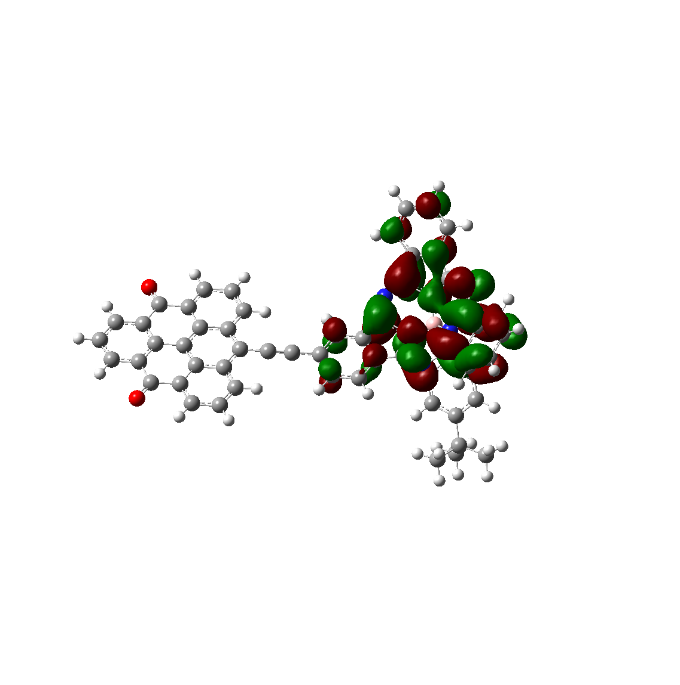

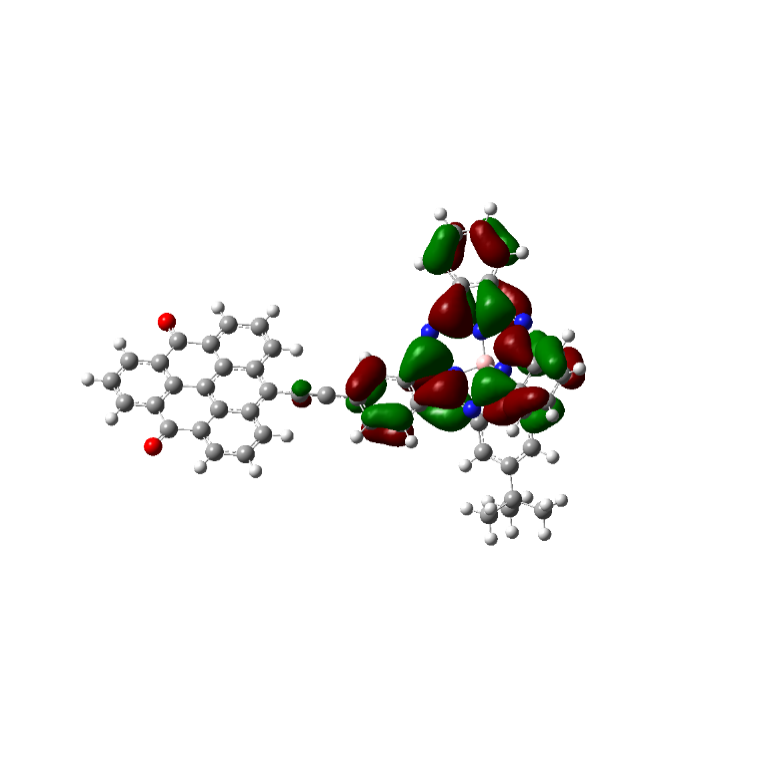


Figure S80: NTO for second excitation (480 nm) of **1**.

0.57

0.41


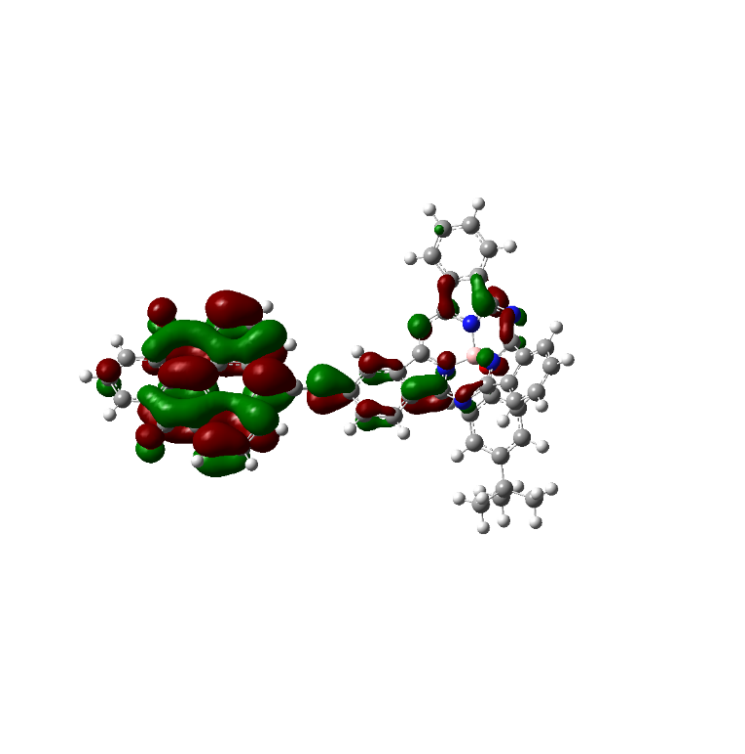

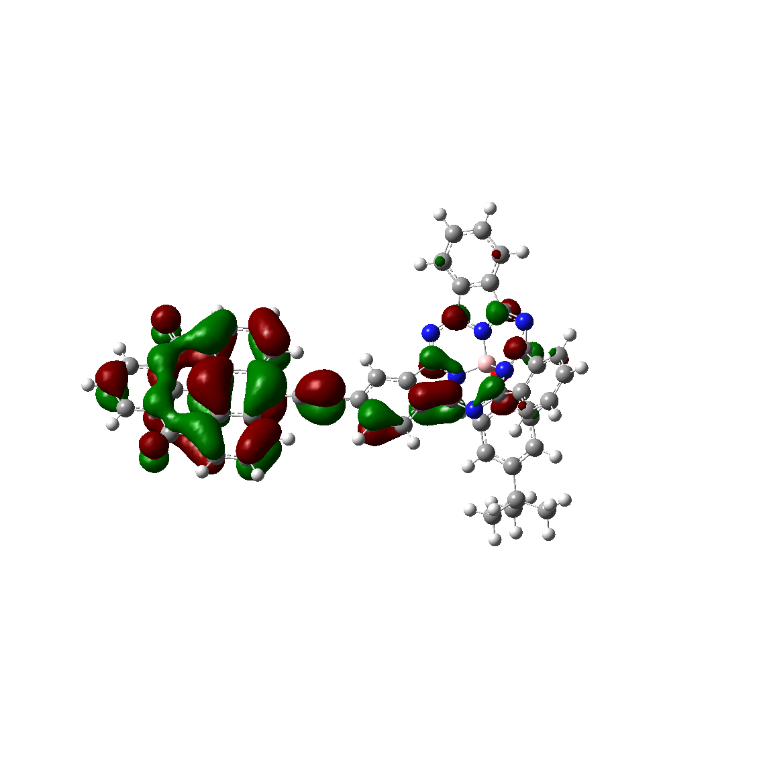

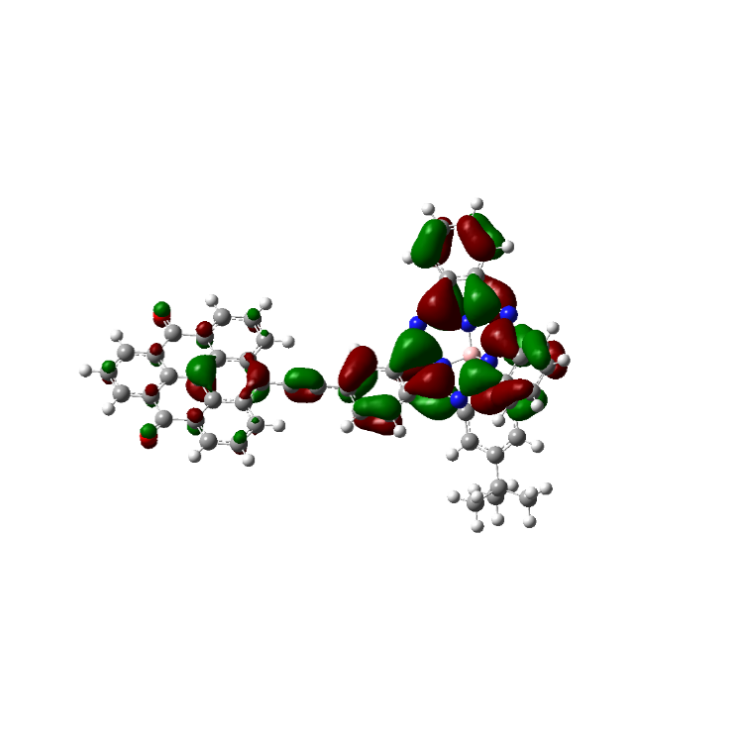

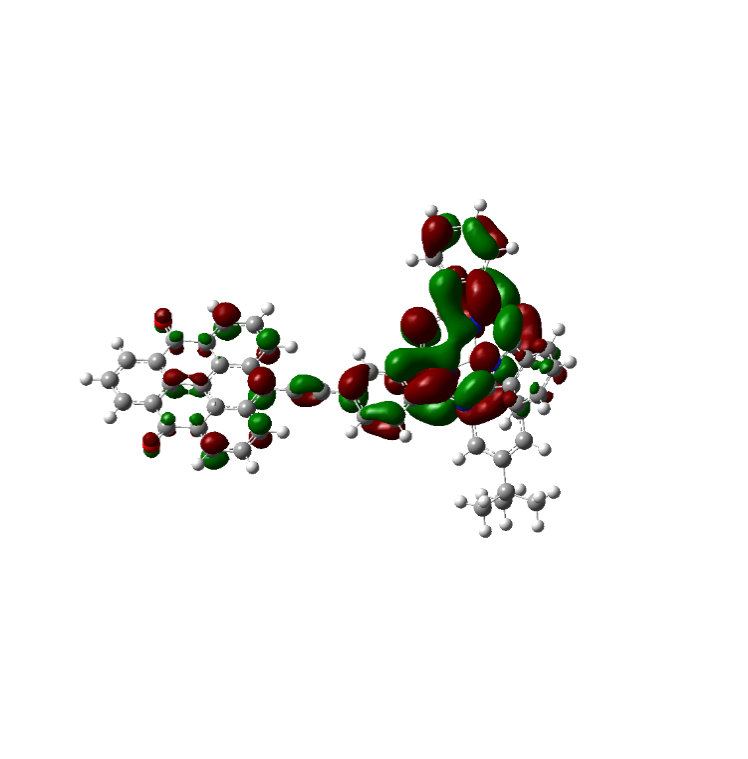


Figure S81: NTO for third excitation (476 nm) of **1**.

0.97


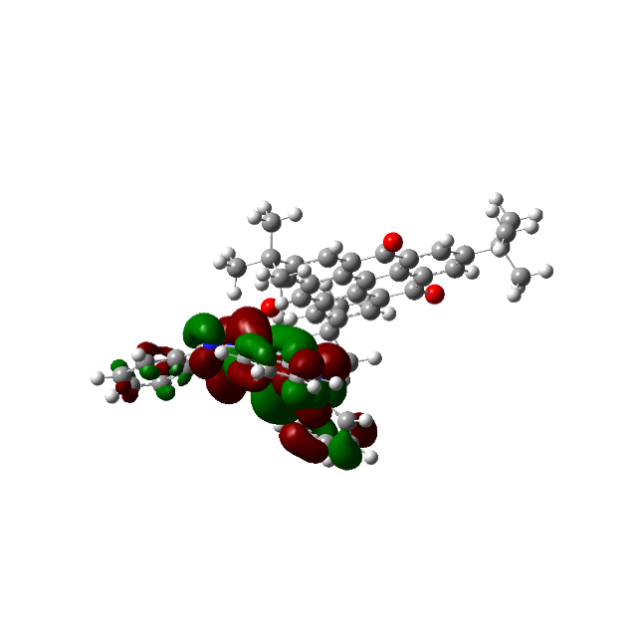

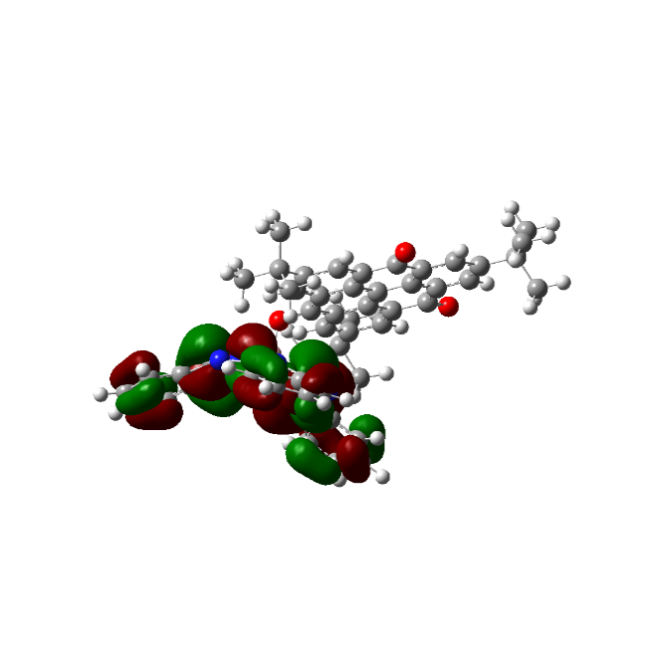


Figure S82: NTO for first excitation (493 nm) of **2**.

0.96


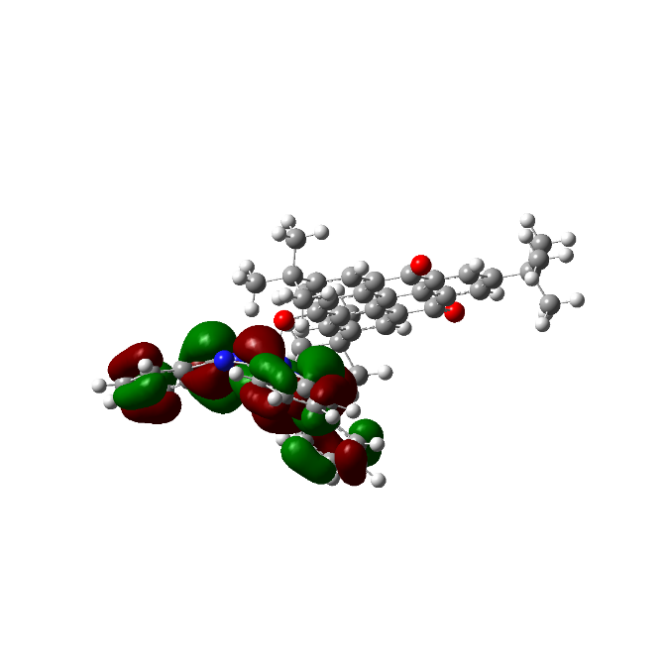

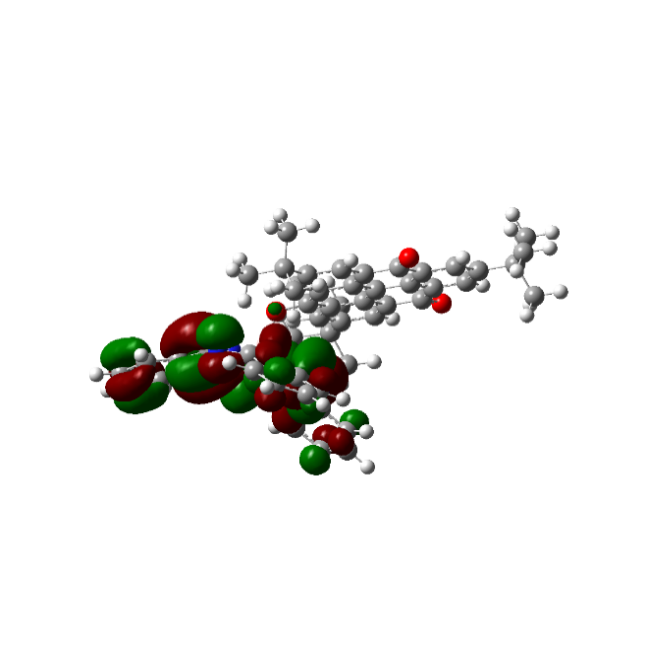


Figure S83: NTO for second excitation (484 nm) of **2**.

0.96


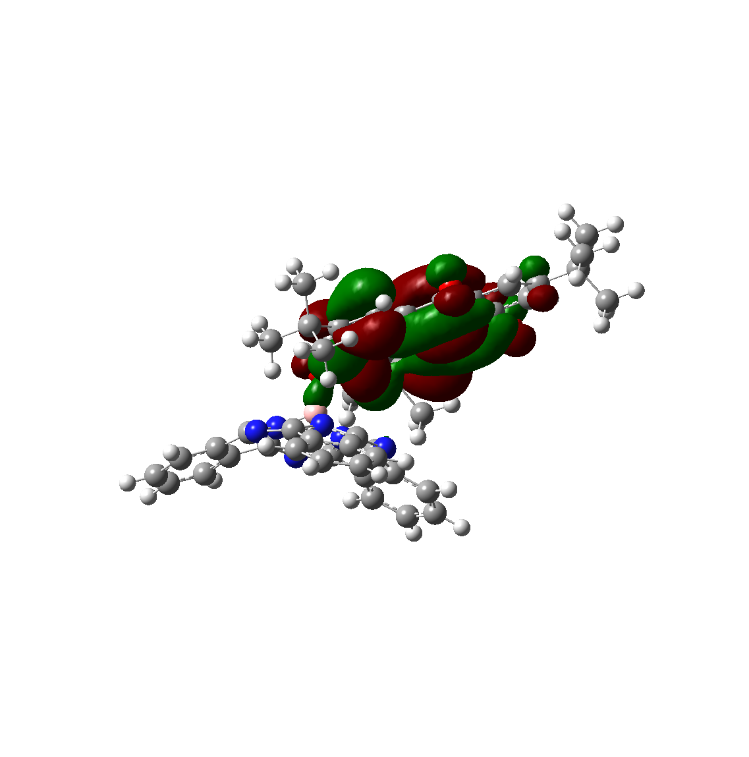

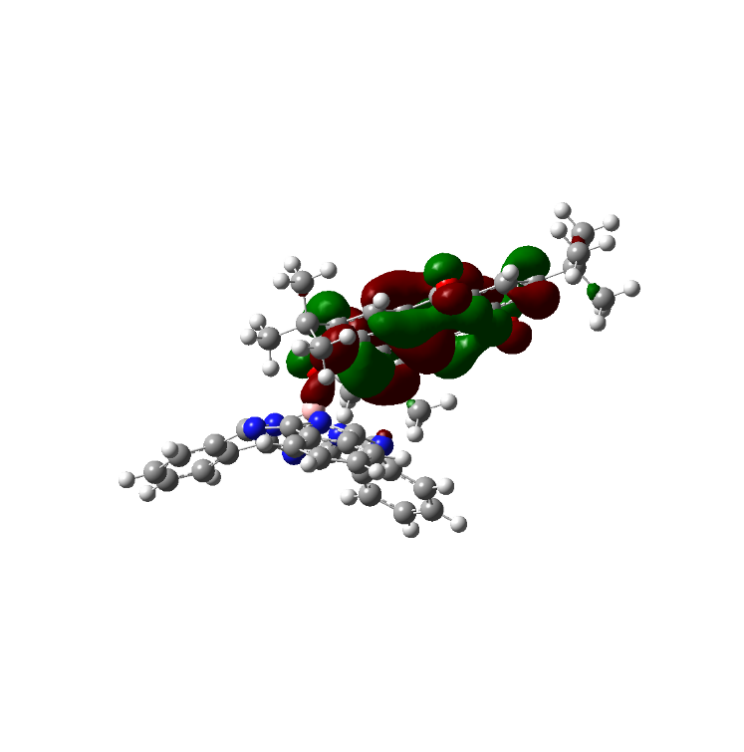


Figure S84: NTO for third excitation (461 nm) of **2**.

**Molecular Orbitals of 1**

Molecular orbitals (HOMO-3 to LUMO+3) of **1**, calculated with M06-2X/6-31(d,p)**.**

**
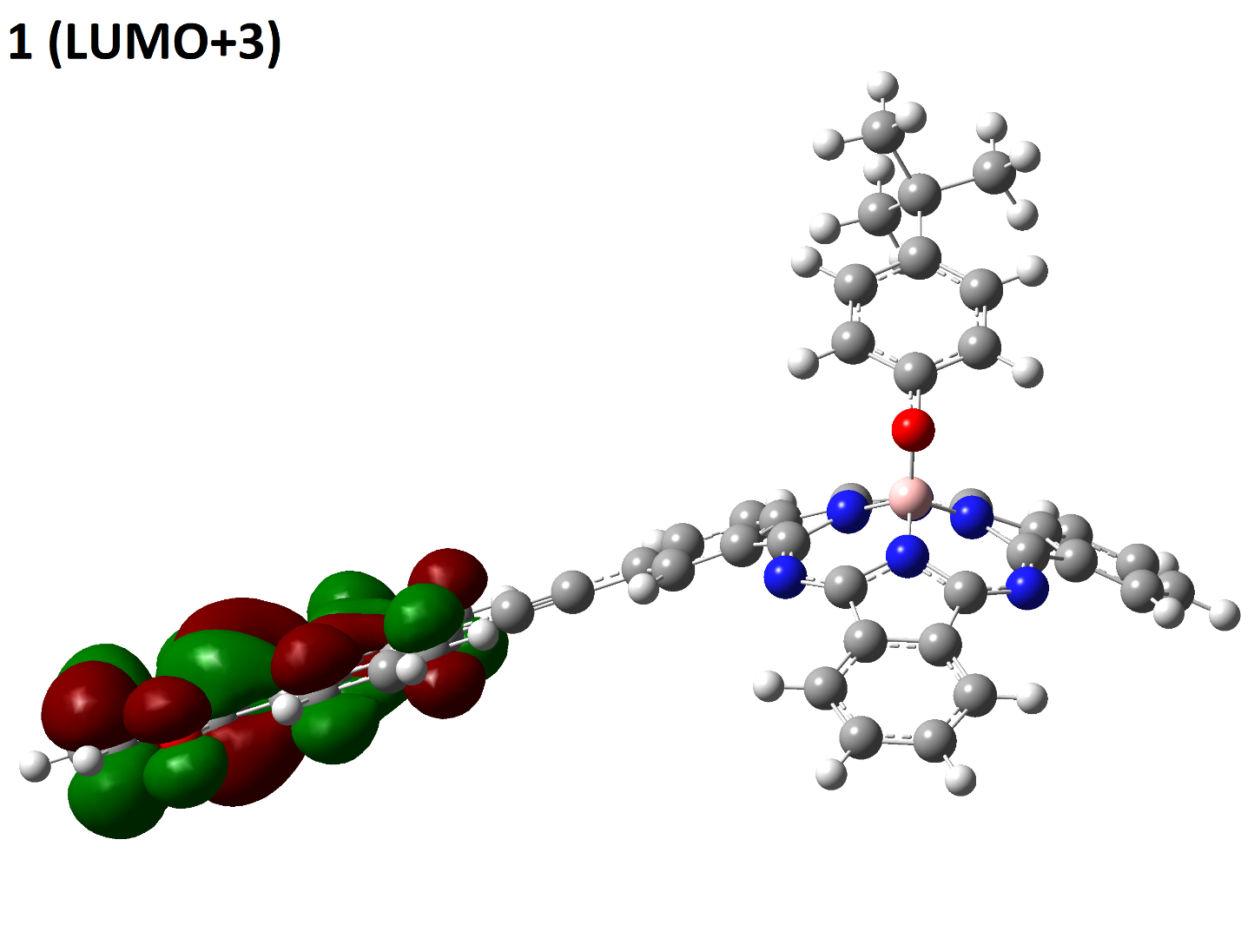
**

Figure S85: LUMO+3 of **1** calculated with M06-2X/6-31g(d,p).

**
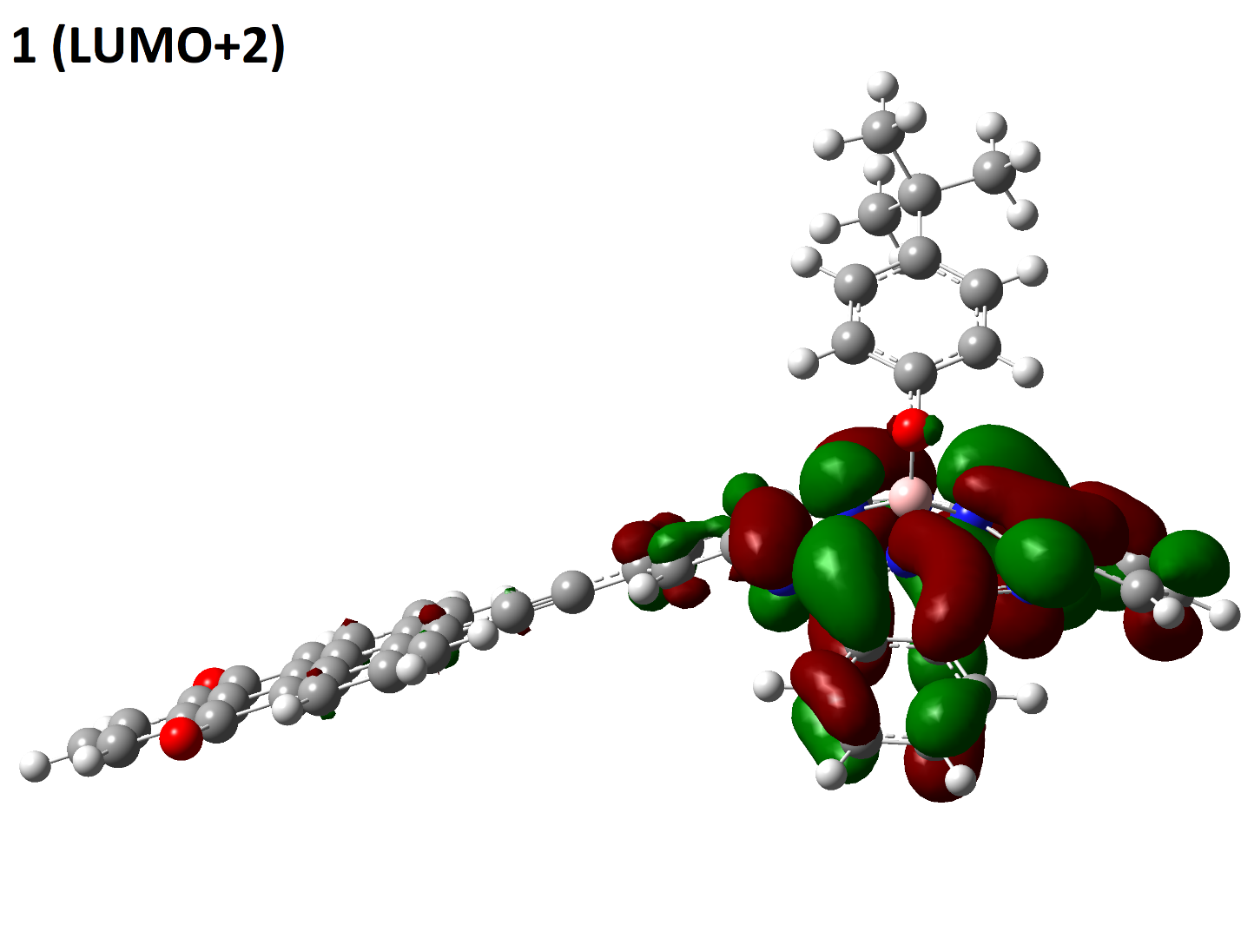
**

Figure S86: LUMO+2 of **1** calculated with M06-2X/6-31g(d,p).

**
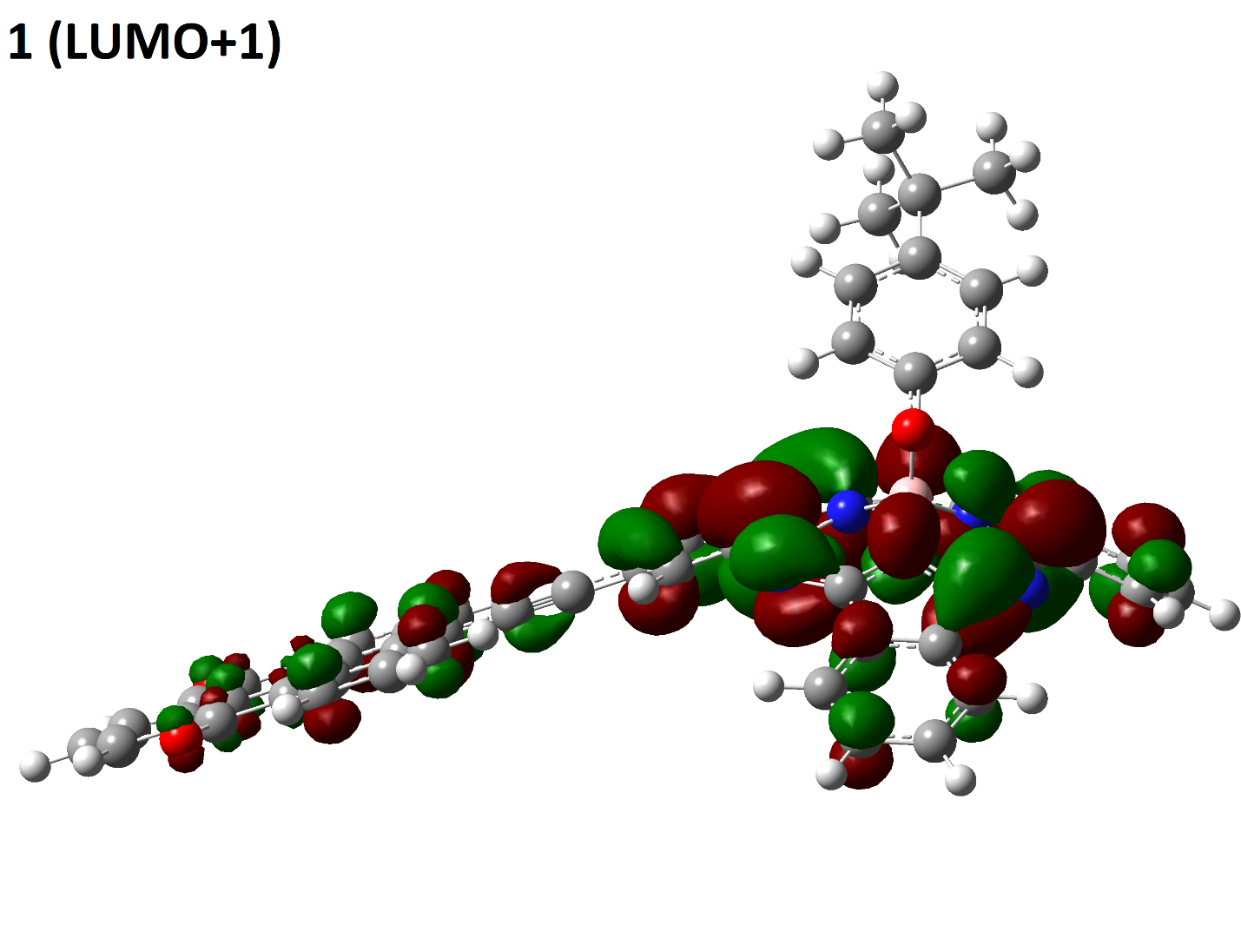
**

Figure S87: LUMO+1 of **1** calculated with M06-2X/6-31g(d,p).

**
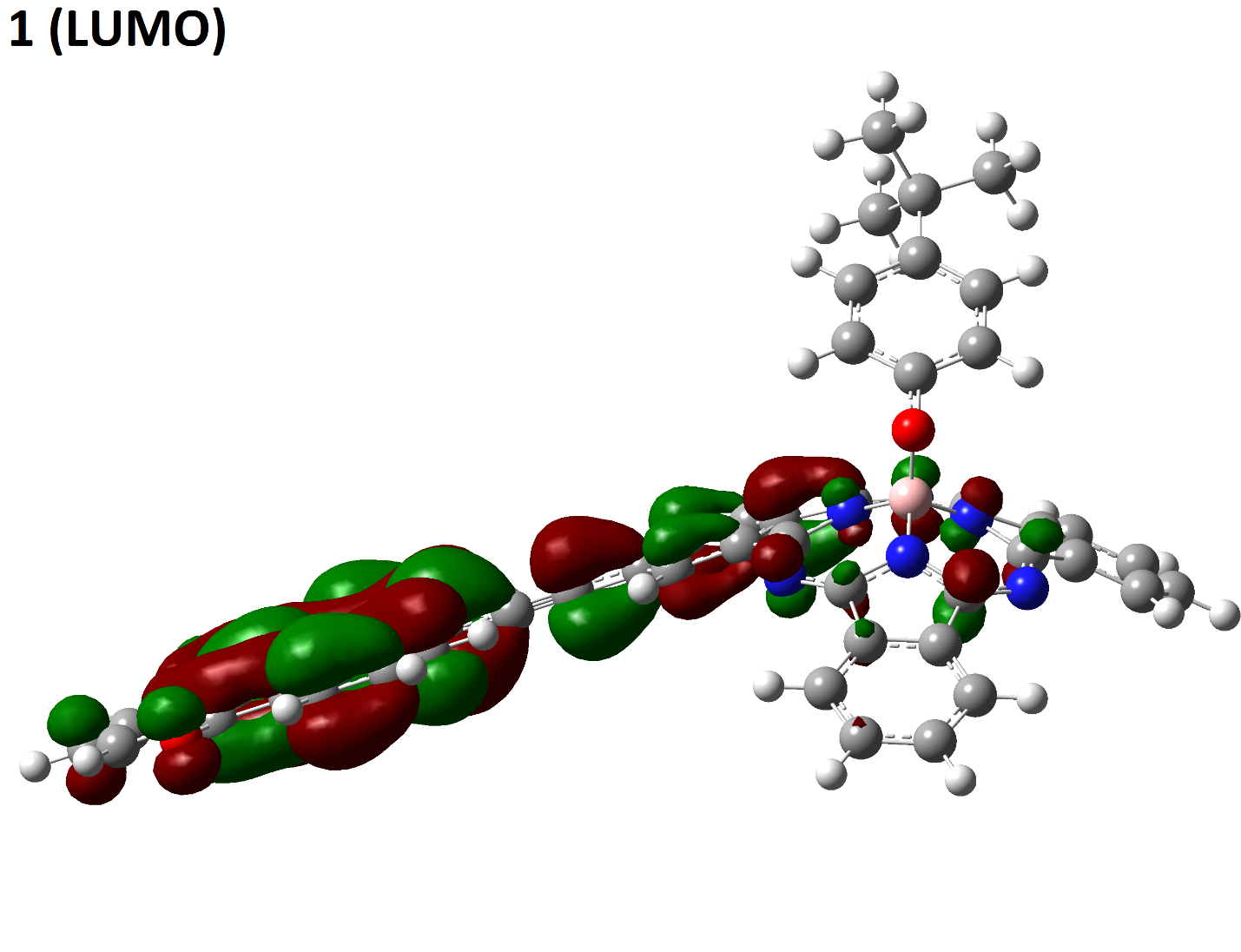
**

Figure S88: LUMO of **1** calculated with M06-2X/6-31g(d,p).

**
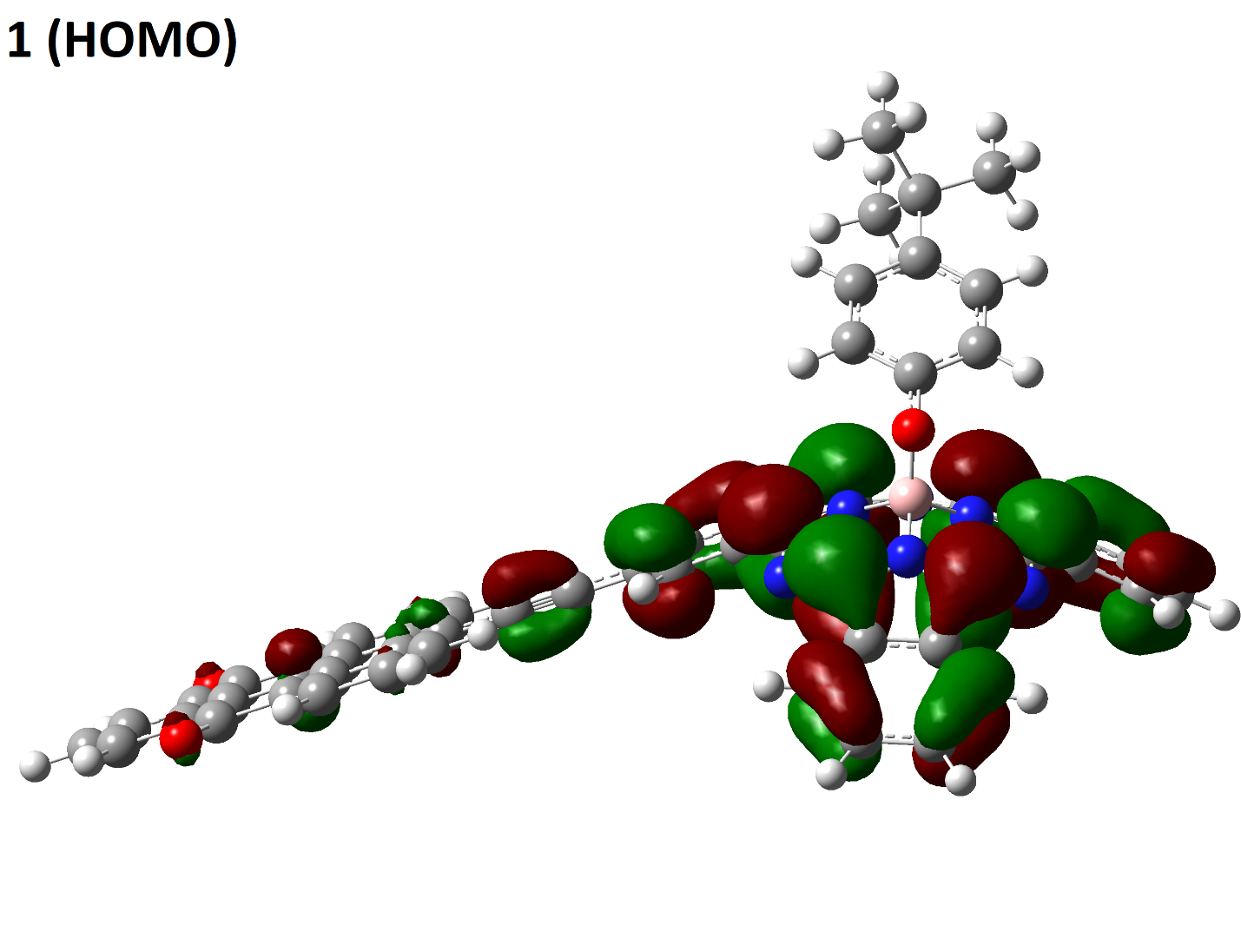
**

Figure S89: HOMO of **1** calculated with M06-2X/6-31g(d,p).

**
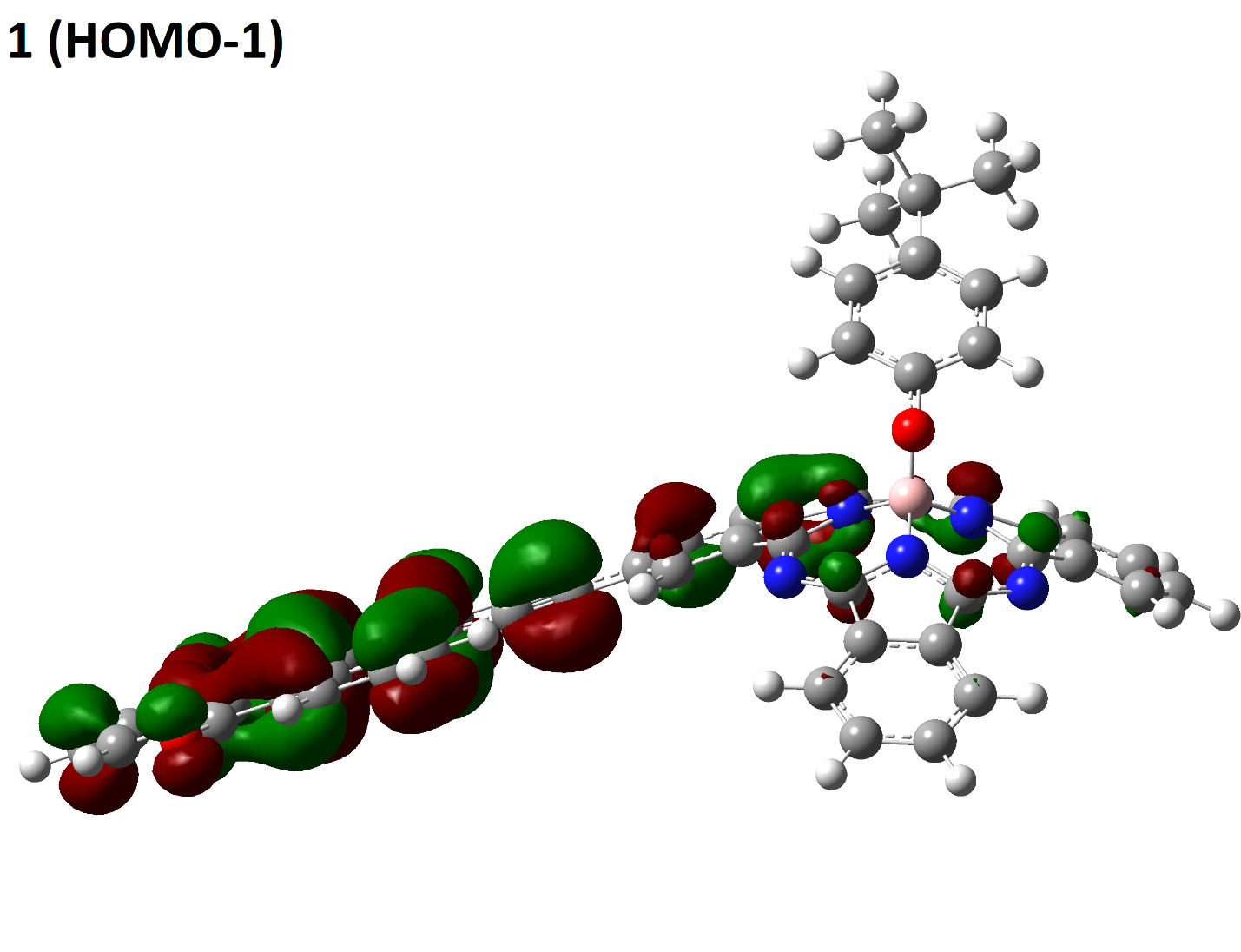
**

Figure S90: HOMO-1 of **1** calculated with M06-2X/6-31g(d,p).

**
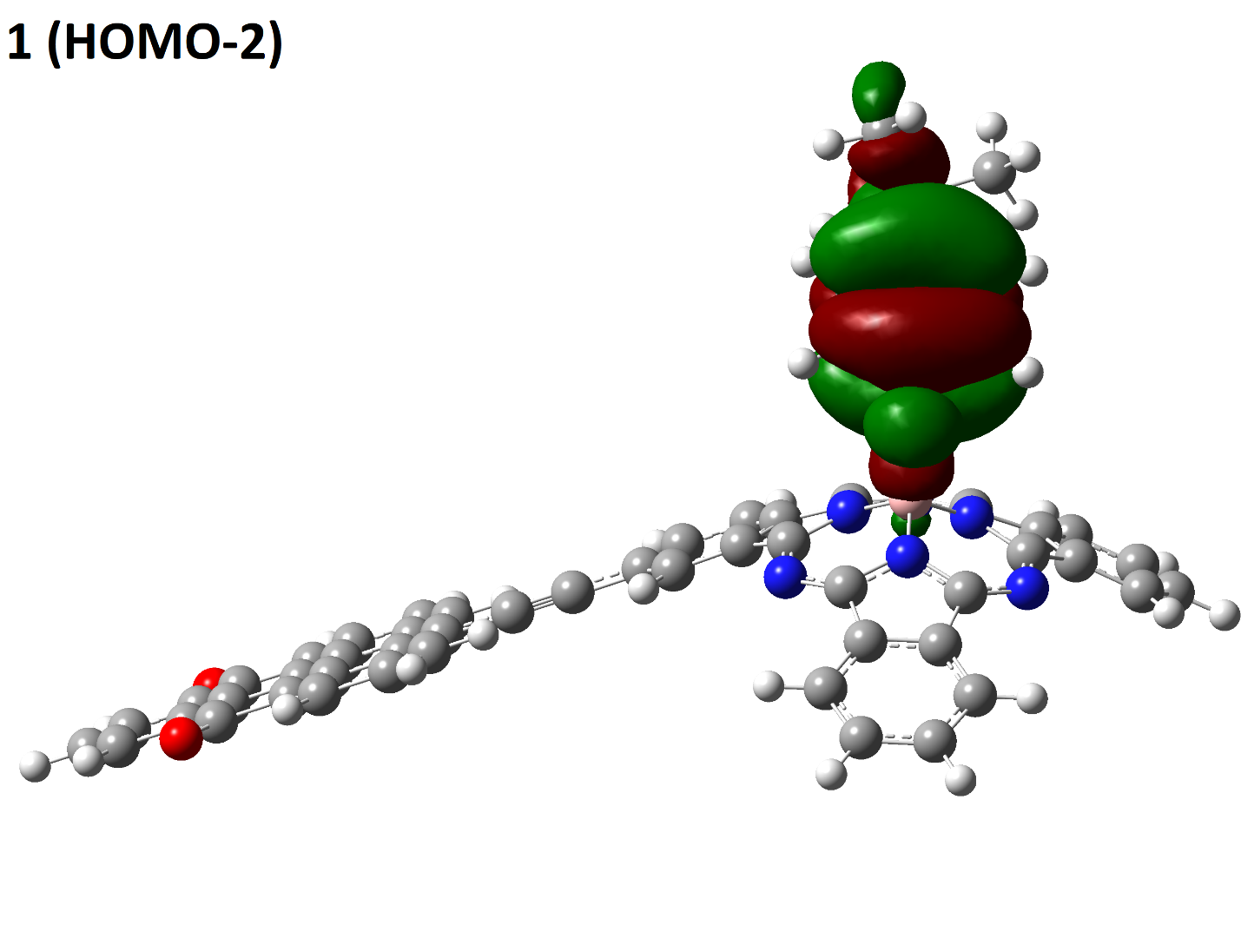
**

Figure S91: HOMO-2 of **1** calculated with M06-2X/6-31g(d,p).

**
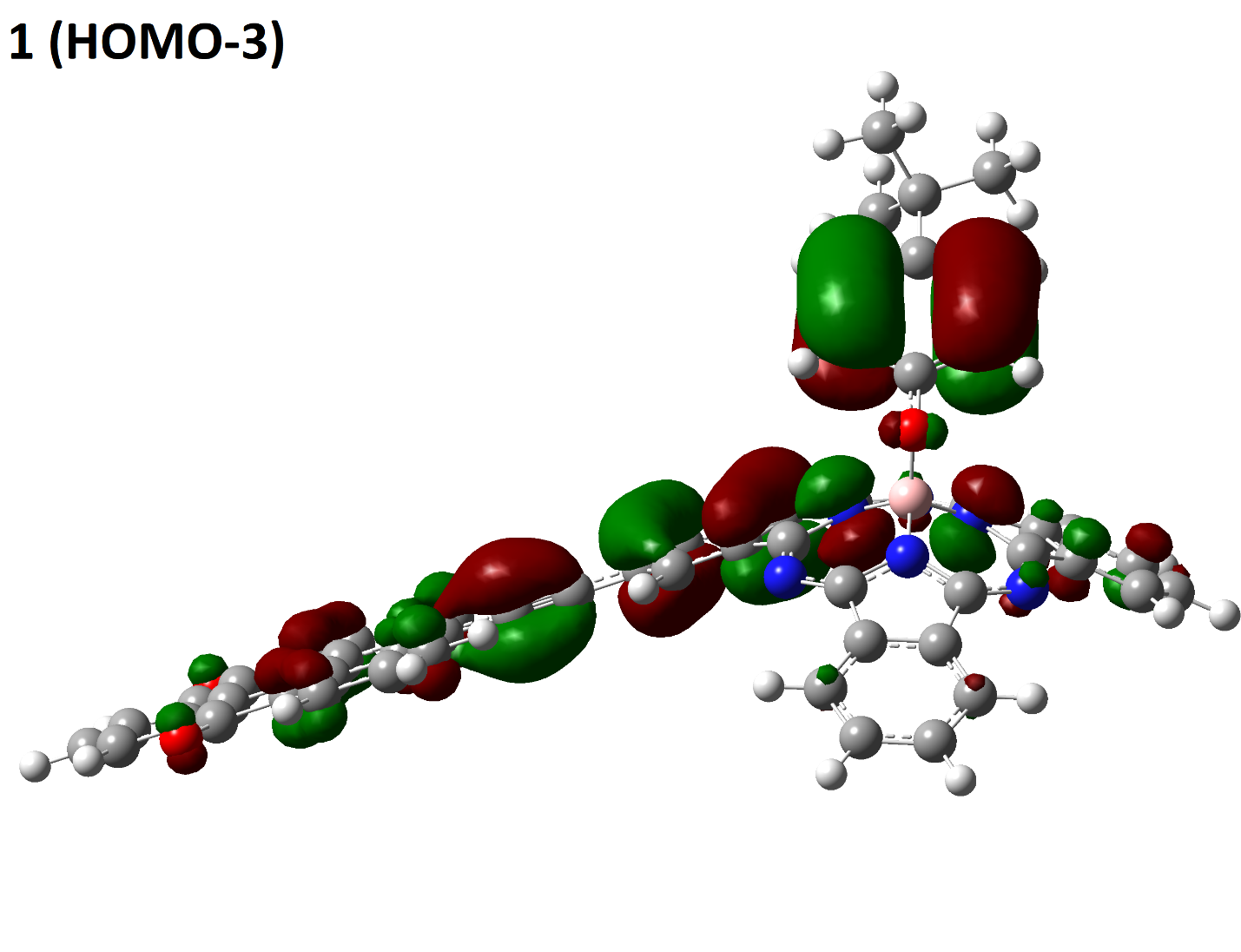
**

Figure S92: HOMO-3 of **1** calculated with M06-2X/6-31g(d,p).

**Molecular Orbitals of 2**

Molecular orbitals (HOMO-3 to LUMO+3) of **2**, calculated with M06-2X/6-31(d,p)**.**

**
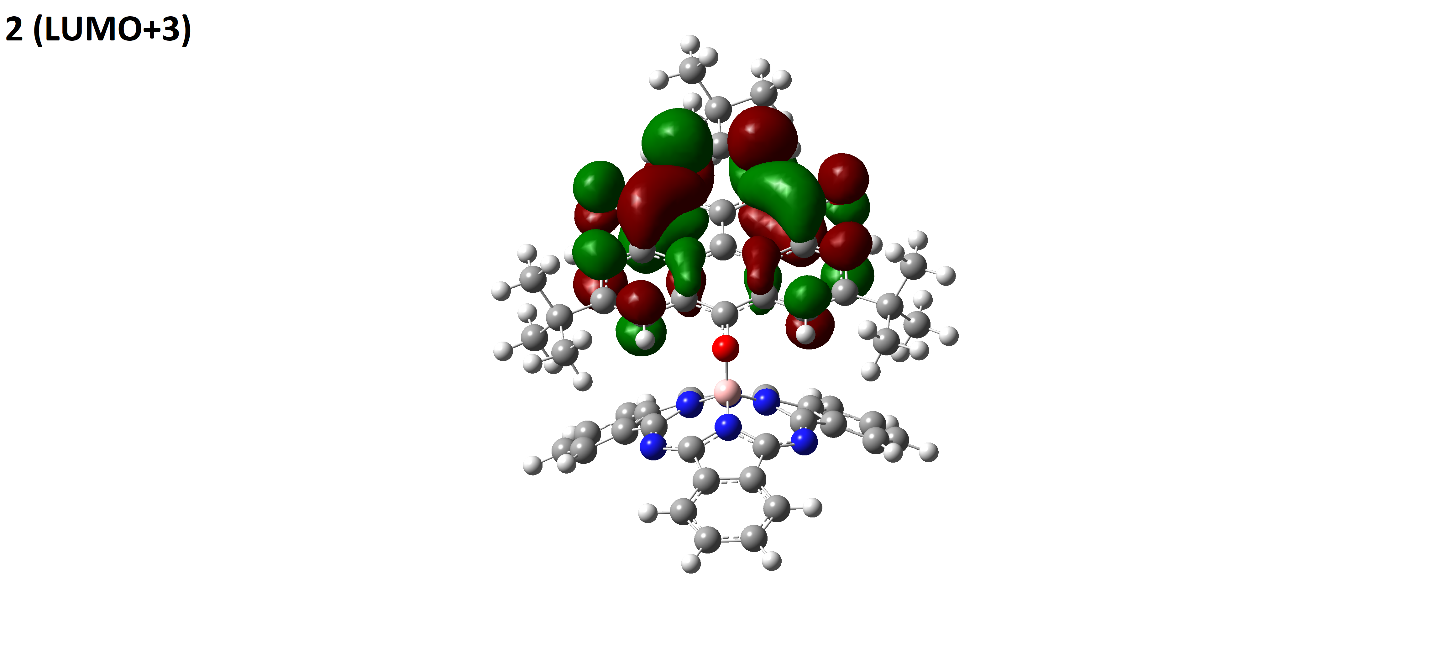
**

Figure S93: LUMO+3 of **2** calculated with M06-2X/6-31g(d,p).

**
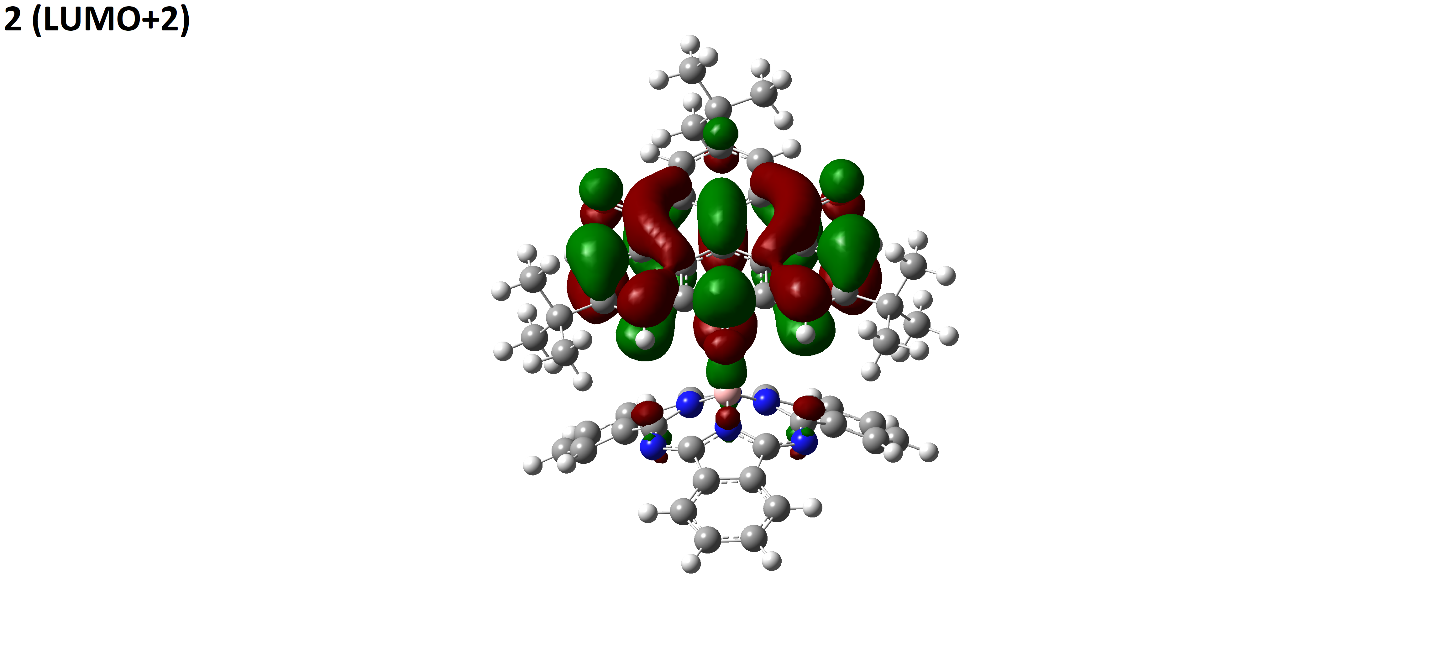
**

Figure S94: LUMO+2 of **2** calculated with M06-2X/6-31g(d,p).

**
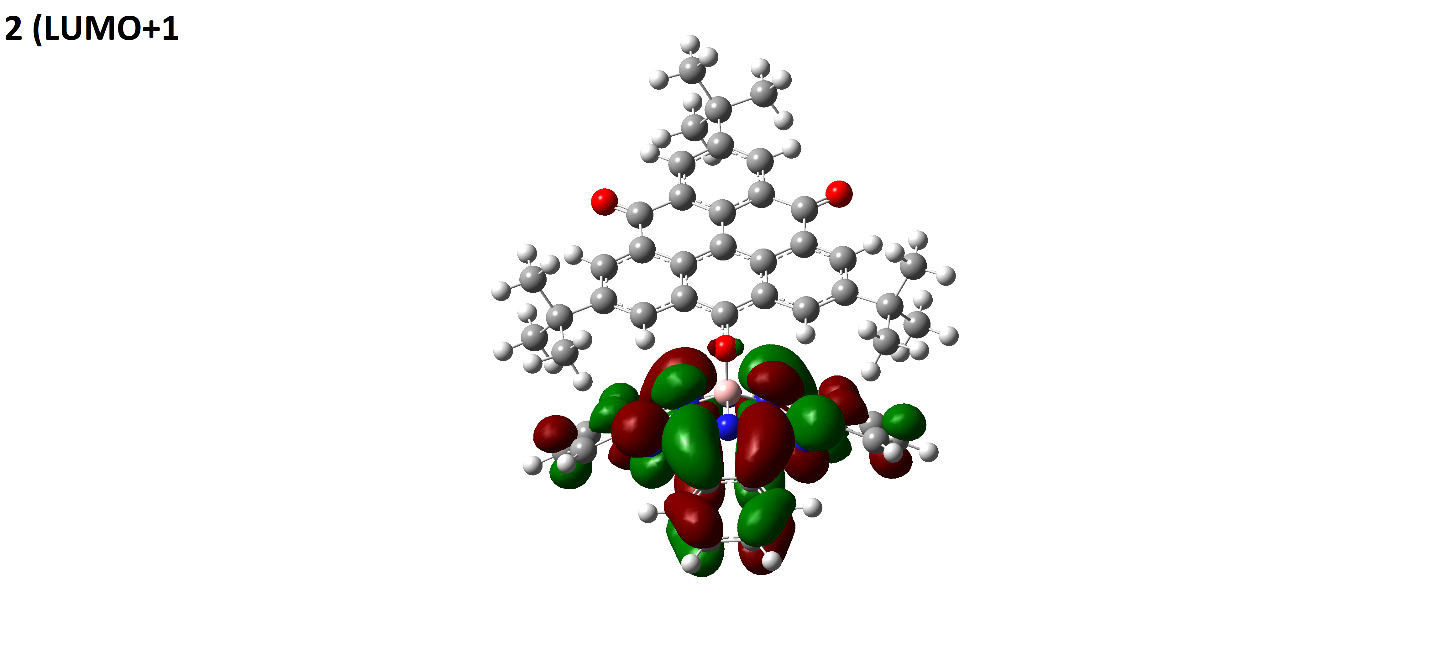
**

Figure S95: LUMO+1 of **2** calculated with M06-2X/6-31g(d,p).

**
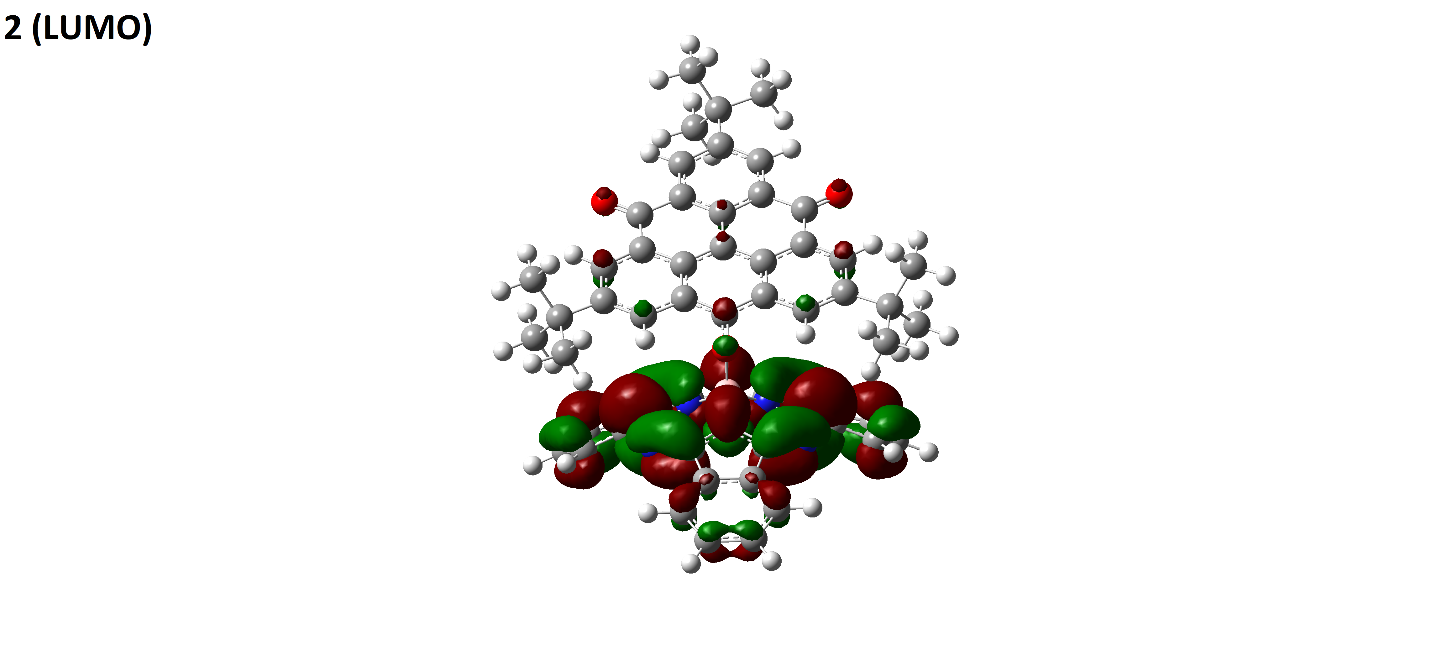
**

Figure S96: LUMO of **2** calculated with M06-2X/6-31g(d,p).

**
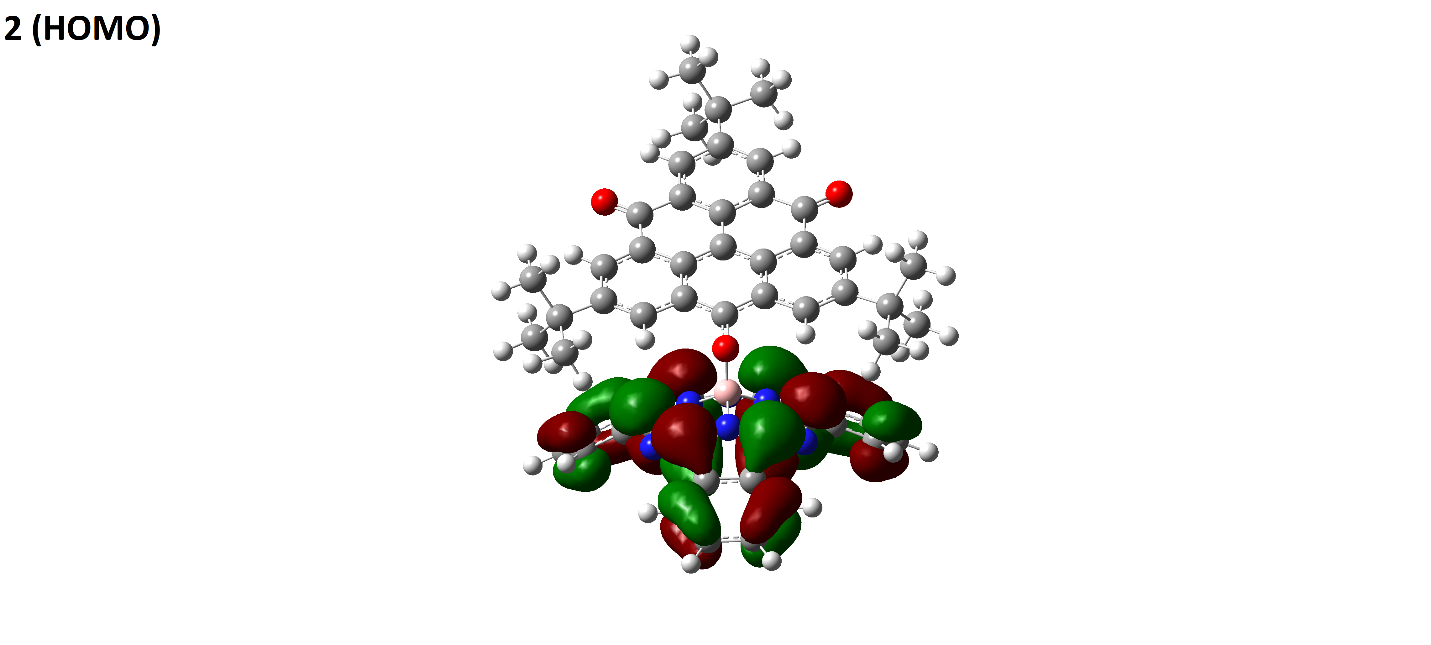
**

Figure S97: HOMO of **2** calculated with M06-2X/6-31g(d,p).

**
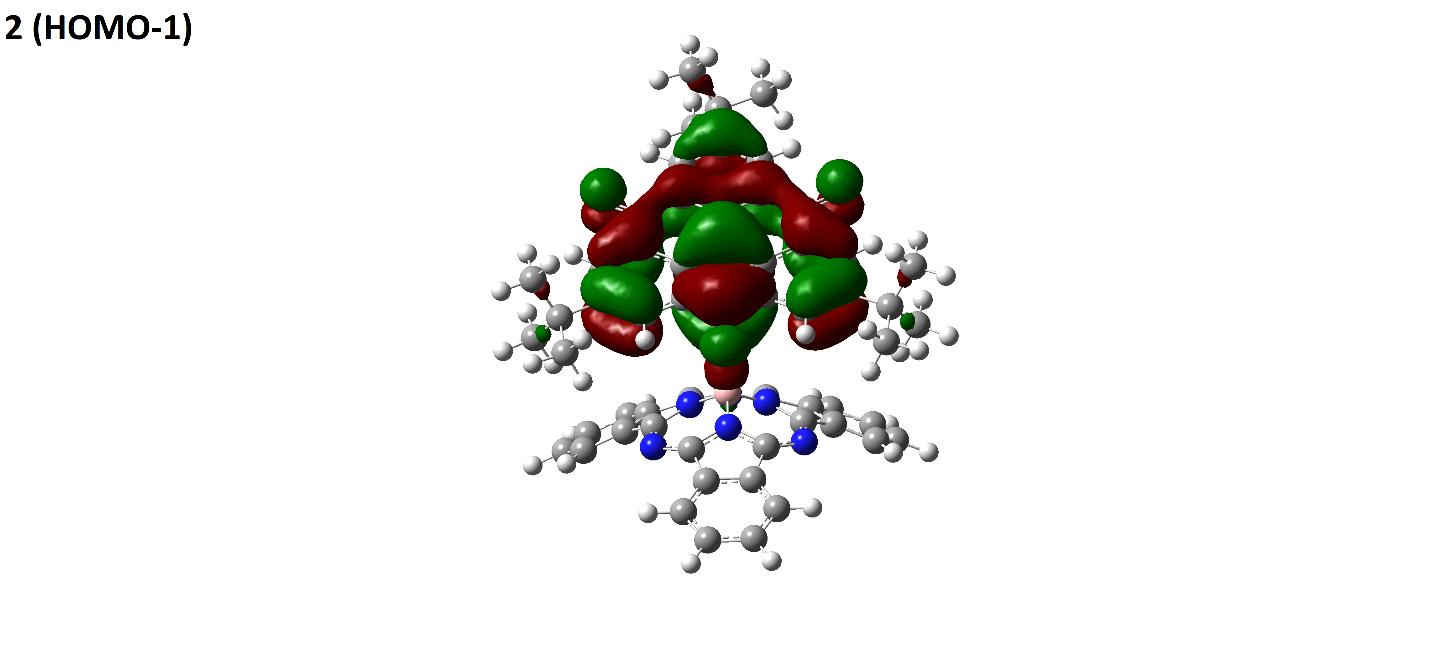
**

Figure S98: HOMO-1 of **2** calculated with M06-2X/6-31g(d,p).

**
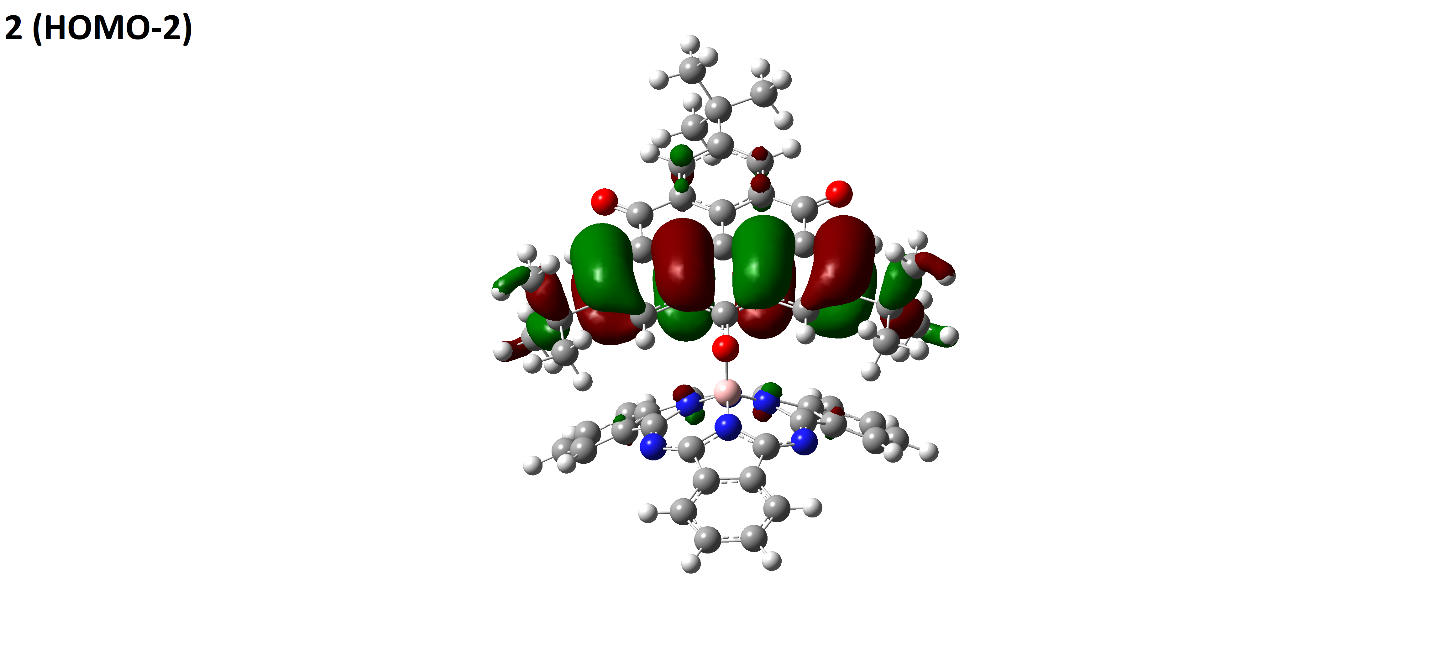
**

Figure S99: HOMO-2 of **2** calculated with M06-2X/6-31g(d,p).

**
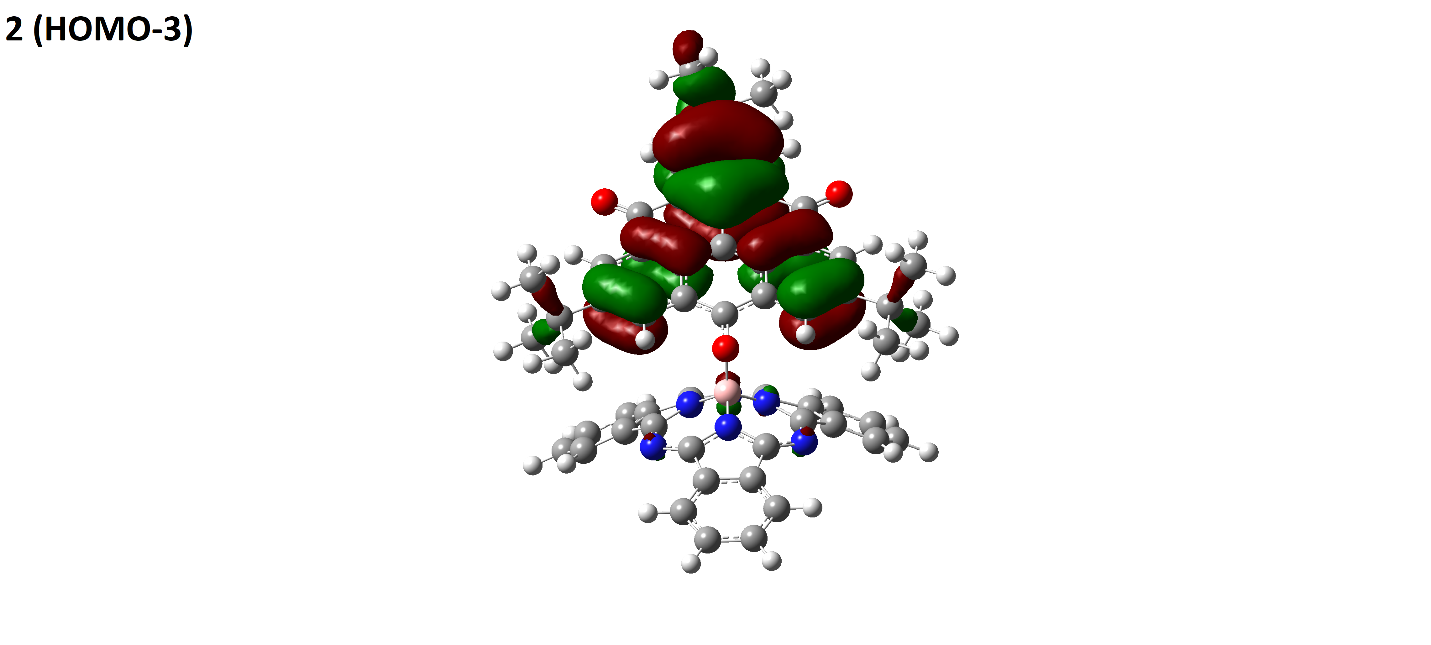
**

Figure S100: HOMO-3 of **2** calculated with M06-2X/6-31g(d,p).

**HOMO orbitals for anionic species calculated with M06-2X/6-31g(d,p)**


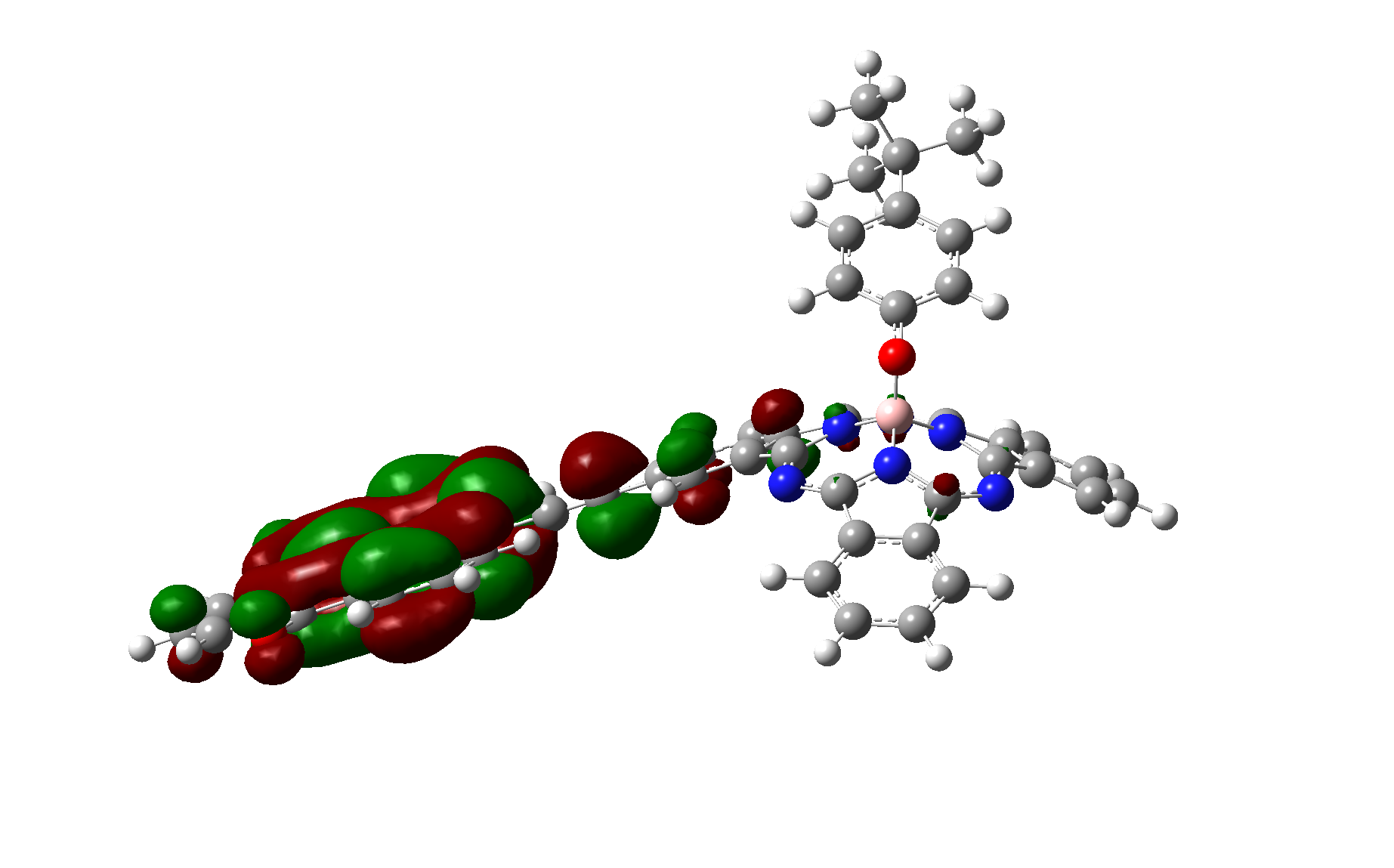


Figure S101: HOMO of **1** anion calculated with M06-2X/6-31g(d,p)..

**
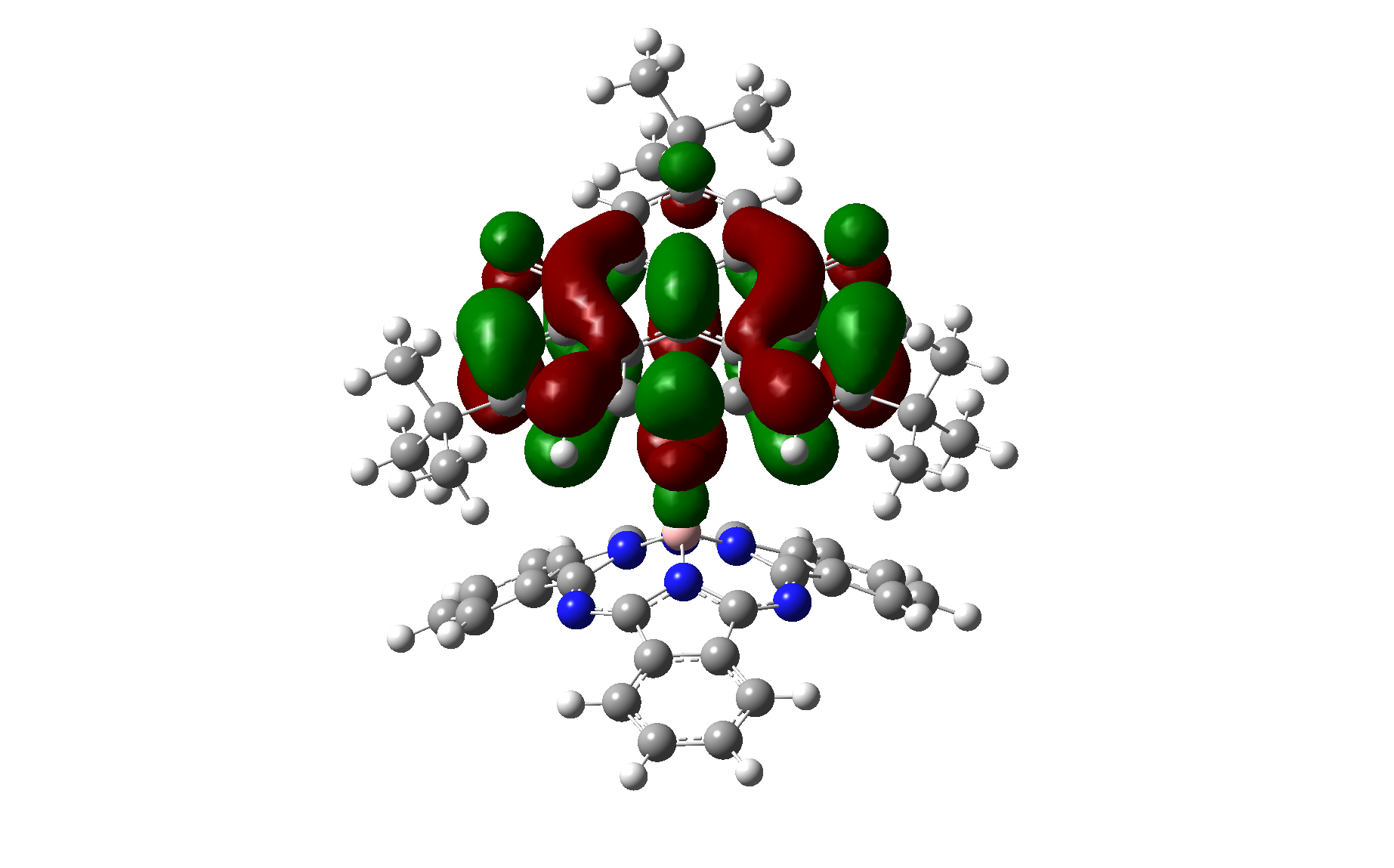
**

Figure S102: HOMO of **2** anion calculated with M06-2X/6-31g(d,p).

**Mulliken Charges**

Mulliken charges shown for optimized neutral, anion and cation geometries for **1** and **2** calculated with CAM-B3LYP/6-31+(d,p).

**
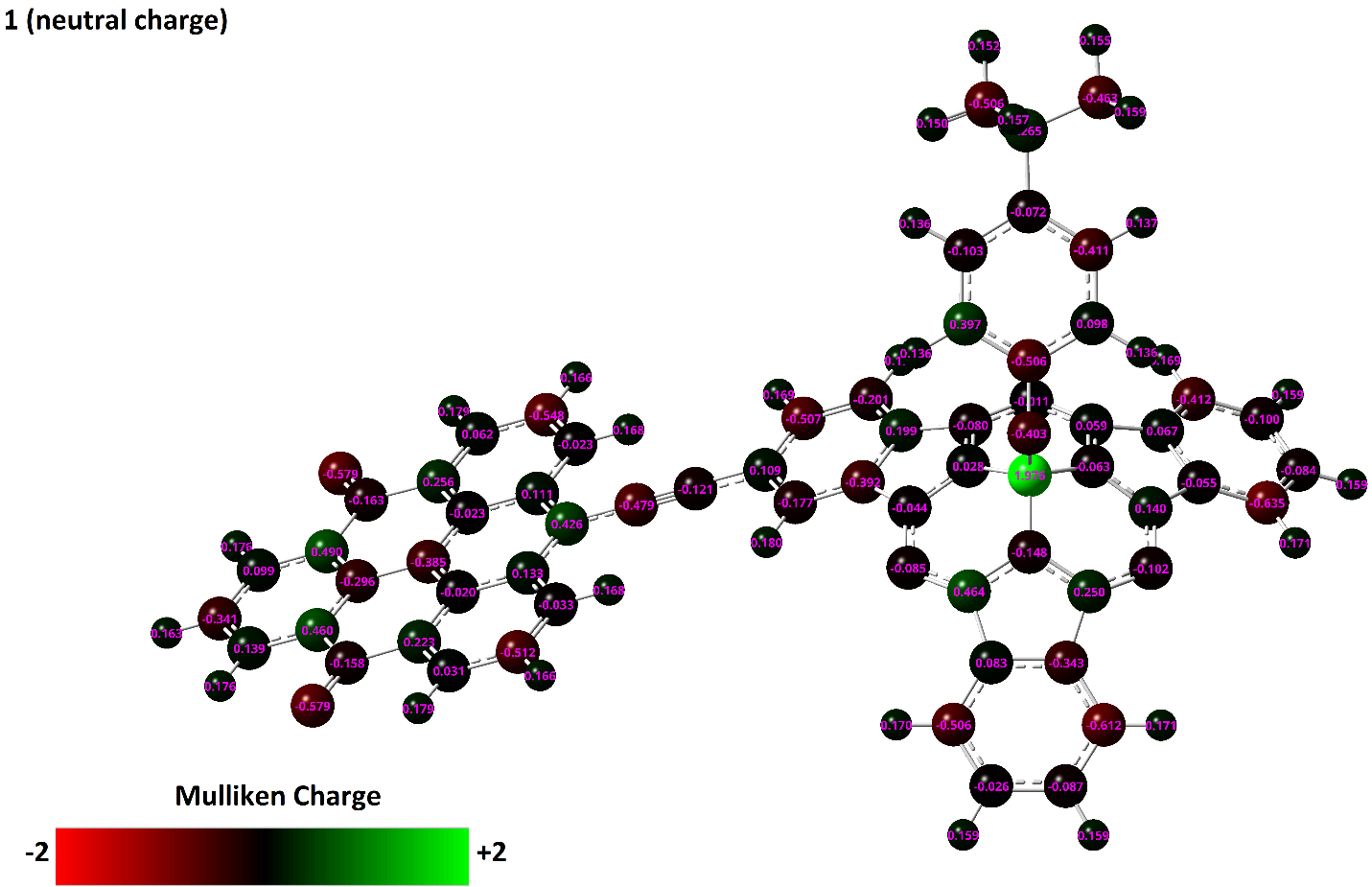
**

Figure S103: Mulliken charges for optimized geometry of **1** calculated with CAM-B3LYP/6-31+g(d,p).

**
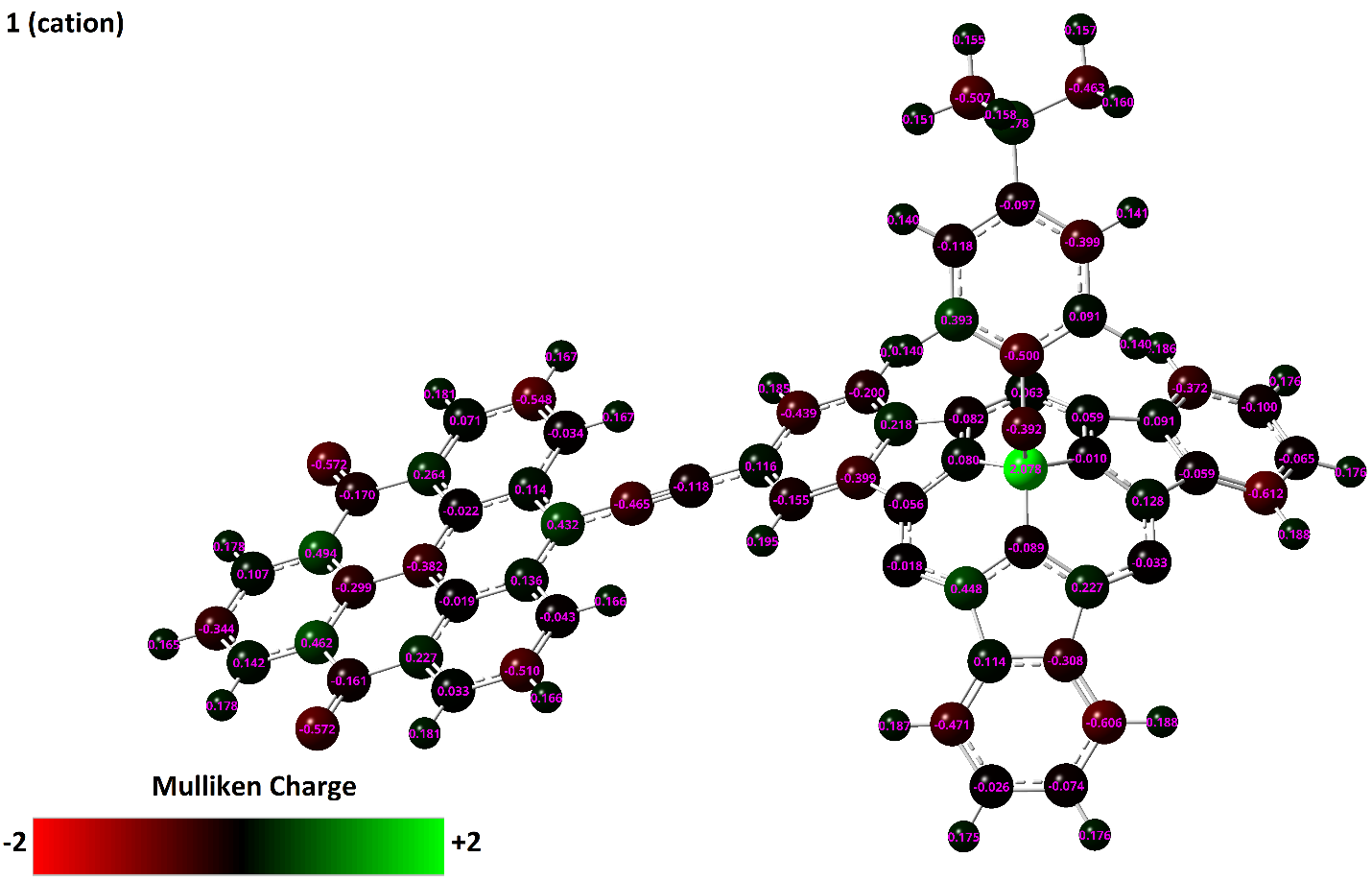
**

Figure S104: Mulliken charges for optimized cationic geometry of **1** calculated with CAM-B3LYP/6-31+g(d,p).

**
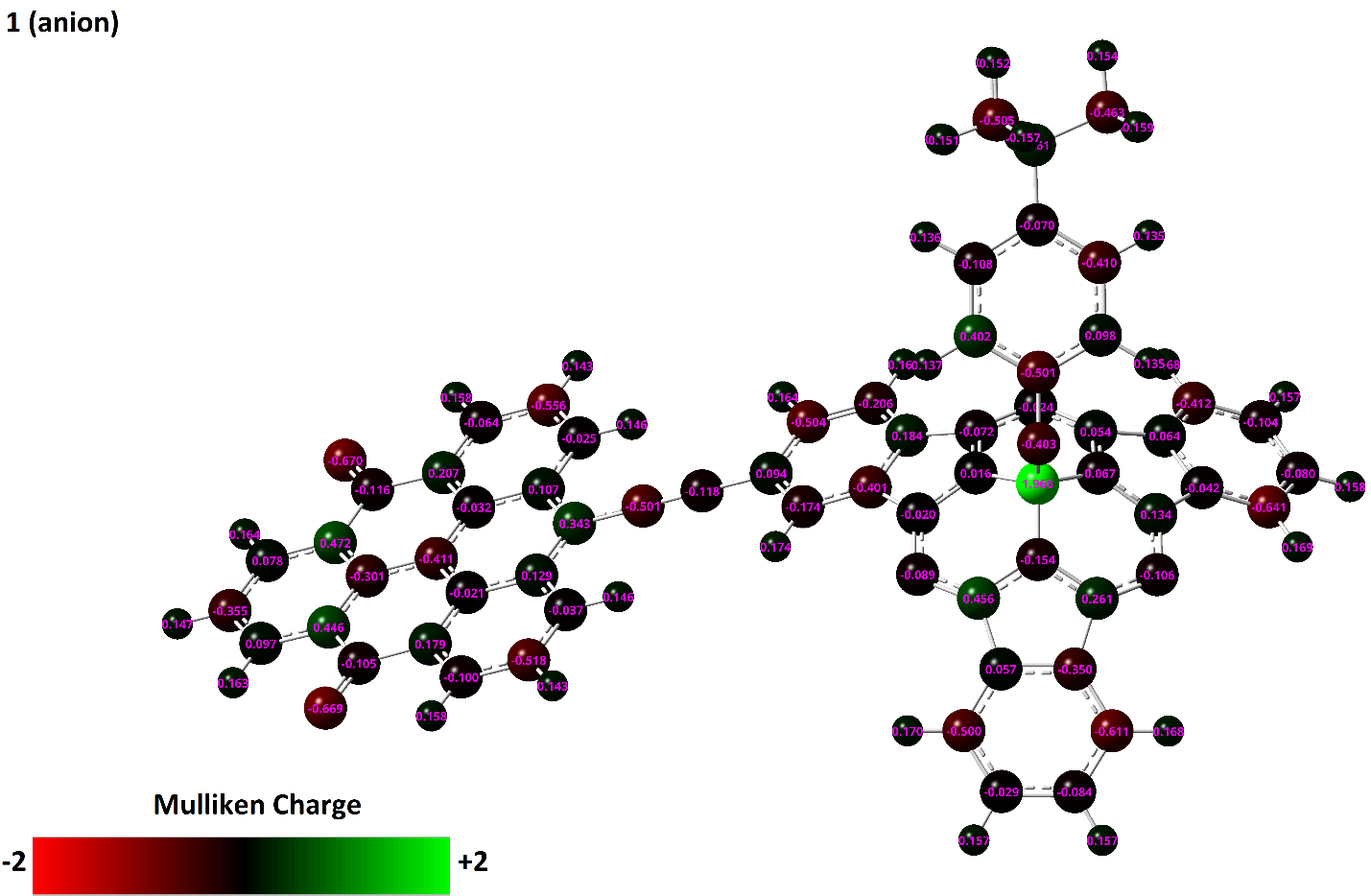
**

Figure S105: Mulliken charges for optimized anionic geometry of **1** calculated with CAM-B3LYP/6-31+g(d,p).

**
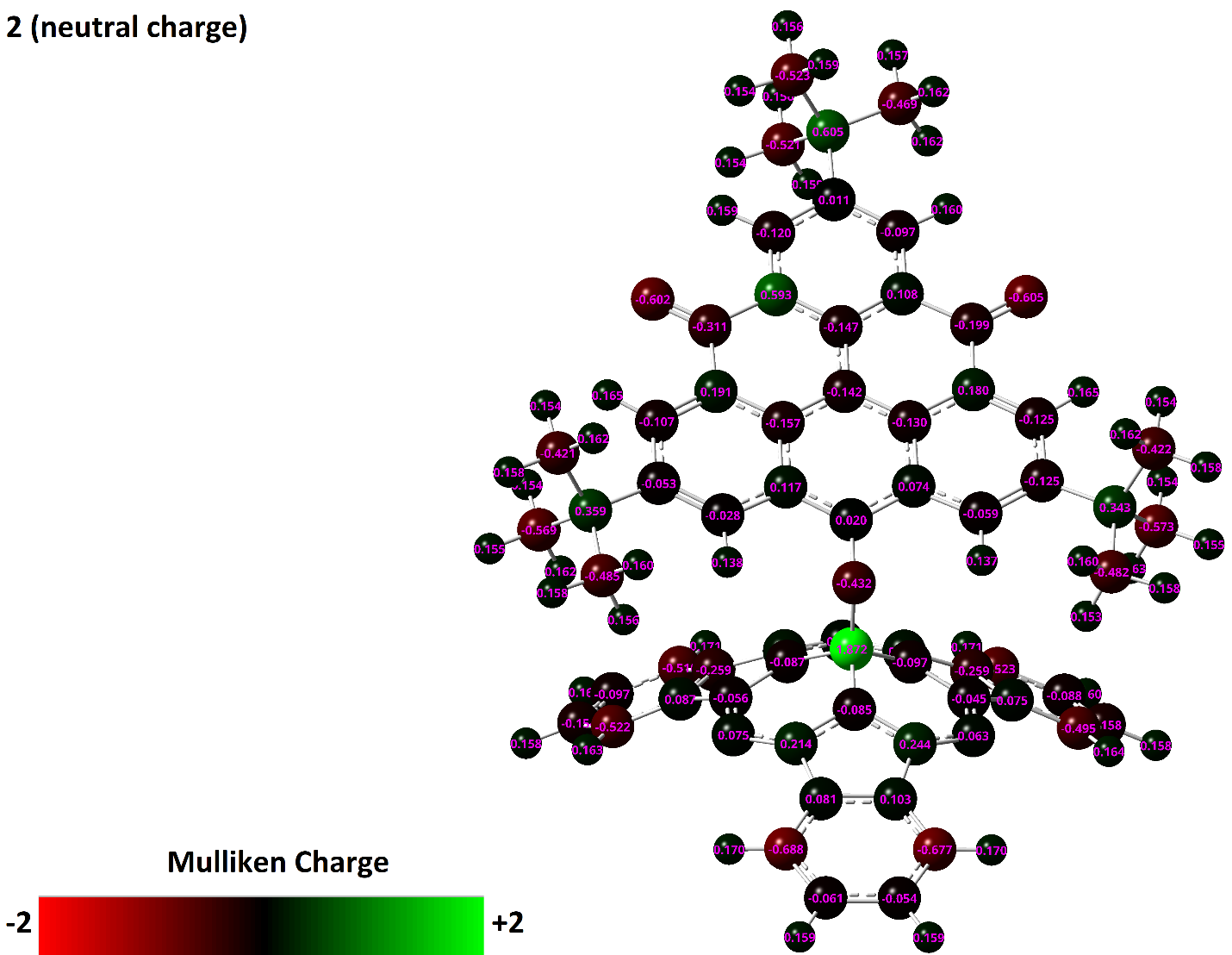
**

Figure S106: Mulliken charges for optimized geometry of **2** calculated with CAM-B3LYP/6-31+g(d,p).

**
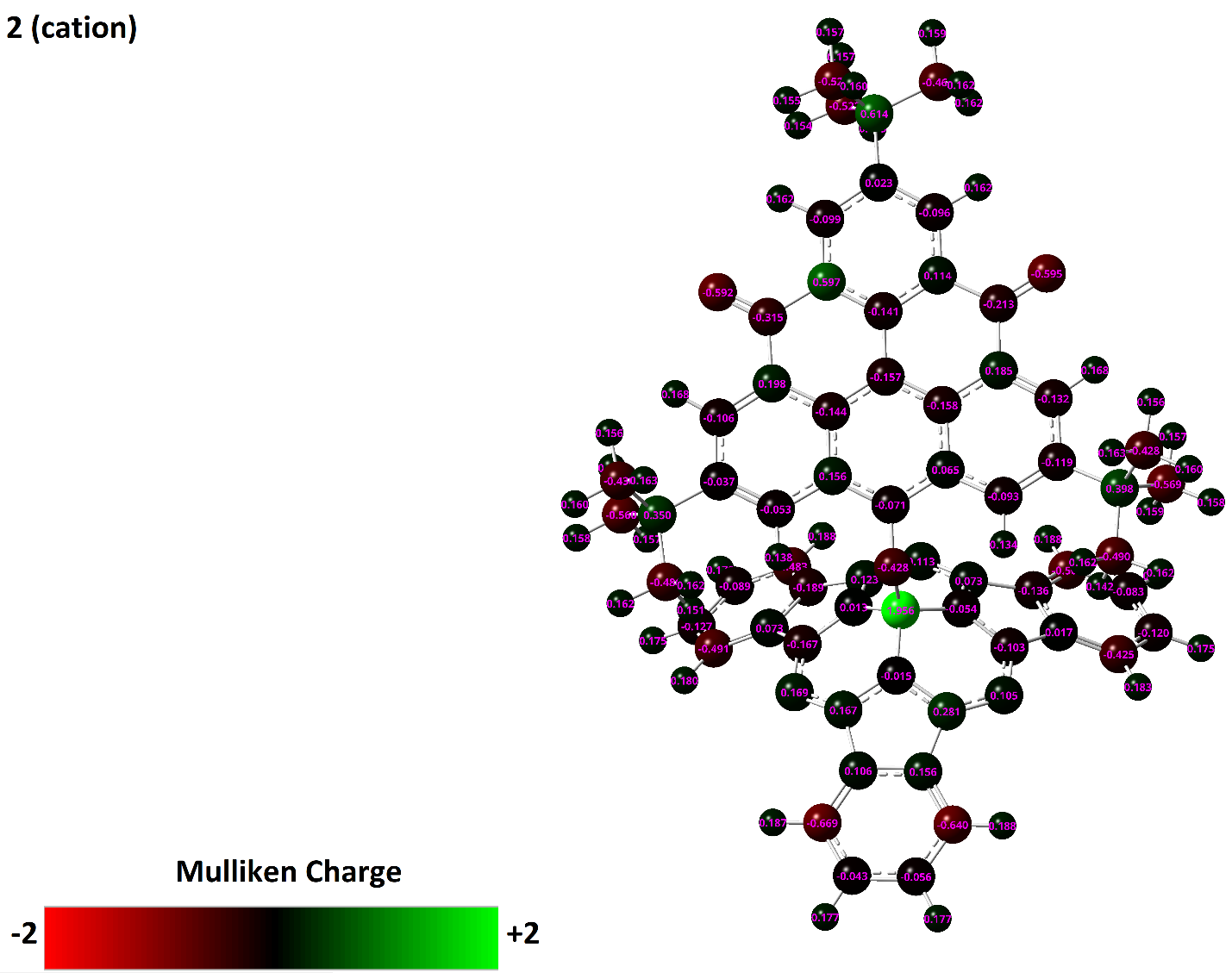
**

Figure S107: Mulliken charges for optimized cationic geometry of **2** calculated with CAM-B3LYP/6-31+g(d,p).

**
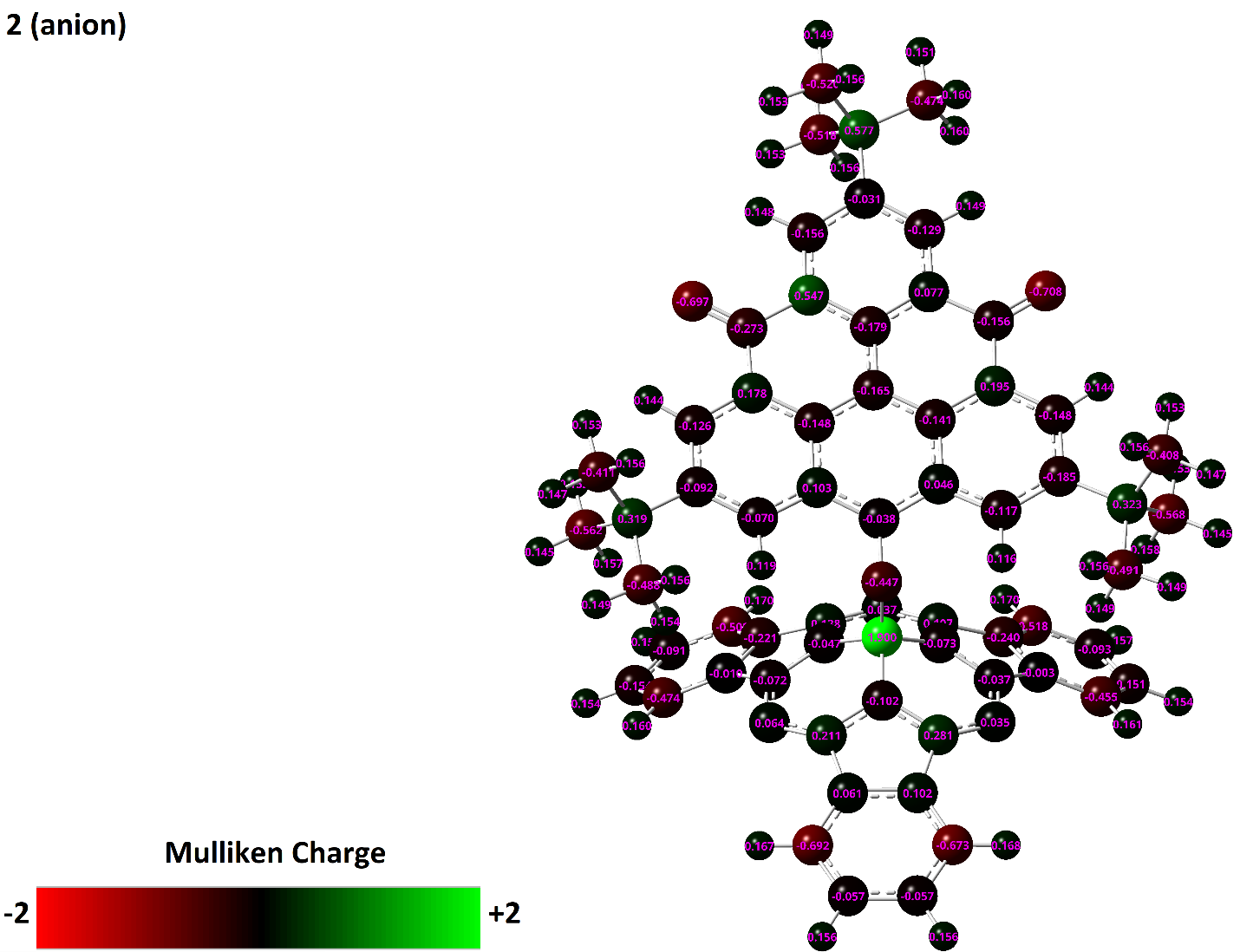
**

Figure S108: Mulliken charges for optimized anionic geometry of **2** calculated with CAM-B3LYP/6-31+g(d,p).

**Calculated electrochemical properties**

Table S17: Calculated redox potentials using CAM-B3LYP/6-31g(d,p) and CAM-B3LYP/6-31+g(d,p), compared with experiments.

|  | ***E*_red, calc_ (V)^b^** | ***E*_red_ (V) ^a^** | ***E*_ox,calc_ (V)^b^** | ***E*_ox_ (V) ^a^** | ***E*_HOMO_ (eV) ^c^** | ***E*_LUMO_ (eV) ^d^** |
| --- | --- | --- | --- | --- | --- | --- |
| **1** | -1.15 (*-1.48)* | -1.11 | 0.61 (*0.38)* | 0.54 | -6.46 (-*6.23*) | -4.24 (-*3.96*) |
| **2** | -1.62 (*-1.96)* | -1.49 | 0.62 (*0.42)* | 0.66 | -6.51 (-*6.29*) | -4.09 (-*3.82*) |
| **SubPC-Ar** | -1.64 (*-1.96)* | -1.56 | 0.49 (*0.27)* | 0.55 | -6.41 (-*6.17*) | -3.96 (-*3.67*) |
| **3a** | -1.13 (*-1.48)* | - | 1.20 (*0.96)* | - | -7.16 (-*6.89*) | -4.57 (-*4.23*) |
| **4** | -1.16 (*-1.50)* | -1.16 | 1.12 (*0.89)* | 1.25 | -7.07 (-*6.82*) | -4.54 (-*4.22*) |
| **5** | -1.21 (*-1.59)* | -1.27 | - (*0.80)* | 1.01 | -6.86 (-*6.63)* | -4.42 (-*4.13*) |
| **6** | -1.31 (*-1.63)* | -1.29 | 1.08 (*0.88)* | 1.09 | -7.03 (-*6.81*) | -4.45 (-*4.15*) |
| **8a** | -1.45 (-1.87) | - | 0.77 (0.52) | - | -6.70 (-6.42) | -4.22 (-*3.86*) |

*^a^* First oxidation and reduction potentials vs. Fc/Fc^+^ couple. Solvent: CH_2_Cl_2_; supporting electrolyte: 0.1 M Bu_4_NPF_6_; scan rate 0.1 V/s. ­^b^Calculated first reduction and oxidation potentials in CH_2_Cl_2_ derived from eqn. 1. *^c^*HOMO energy level calculated as orbital energy of highest occupied molecular orbital from DFT. *^d^*LUMO energy calculated as ***E*_HOMO_** + TD-DFT(HOMO 🡪LUMO), the results are from CAM-B3LYP/6-31+g(d,p) calculations while numbers in parentheses is with CAM-B3LYP/6-31g(d,p).

Table S18: Calculated redox potentials using M06-2X/6-31g(d,p) and M06-2X /6-31+g(d,p), compared with experiments.

|  | ***E*_red, calc_ (V)^b^** | ***E*_red_ (V) ^a^** | ***E*_ox,calc_ (V)^b^** | ***E*_ox_ (V) ^a^** | ***E*_HOMO_ (eV) ^c^** | ***E*_LUMO_ (eV) ^d^** |
| --- | --- | --- | --- | --- | --- | --- |
| **1** | -1.07 (*-1.34)* | -1.11 | 0.83 (*0.67)* | 0.54 | -6.45 (-*6.26*) | -4.21 (-*3.97*) |
| **2** | -1.54 (*-1.84)* | -1.49 | 0.87 (*0.69)* | 0.66 | -6.51 (-*6.33*) | -4.04 (-*3.82*) |
| **SubPC-Ar** | -1.56 (*-)* | -1.56 | 0.73 (*0.56)* | 0.55 | -6.39 (-*6.20*) | -3.88 (-*3.64*) |
| **3a** | -1.08 (*-1.38)* | - | 1.40 (*1.22)* | - | -7.11(-*6.90*) | -4.50 (-*4.22*) |
| **4** | -1.10 (*-1.40)* | -1.16 | 1.33 (1.15*)* | 1.25 | -7.03 (-*6.83*) | -4.48 (-*4.22*) |
| **5** | - (*-1.43)* | -1.27 | - (1.03*)* | 1.01 | - (-*6.65)* | - (-*4.14*) |
| **6** | -1.24 (*-1.50)* | -1.29 | 1.27 (1.11*)* | 1.09 | -6.98 (-*6.81*) | -4.41 (-*4.15*) |
| **8a** | -1.46 (-1.73) | - | 0.97 (0.77) | - | -6.65 (-6.43) | -4.19 (-*3.88*) |

*^a^* First oxidation and reduction potentials vs. Fc/Fc^+^ couple. Solvent: CH_2_Cl_2_; supporting electrolyte: 0.1 M Bu_4_NPF_6_; scan rate 0.1 V/s. ­^b^Calculated first reduction and oxidation potentials in CH_2_Cl_2_ derived from eqn. 1. *^c^*HOMO energy level calculated as orbital energy of highest occupied molecular orbital from DFT. *^d^*LUMO energy calculated as ***E*_HOMO_** + TD-DFT(HOMO 🡪LUMO), the results are from M06-2X /6-31+g(d,p) calculations while numbers in parentheses is with M06-2X/6-31g(d,p).


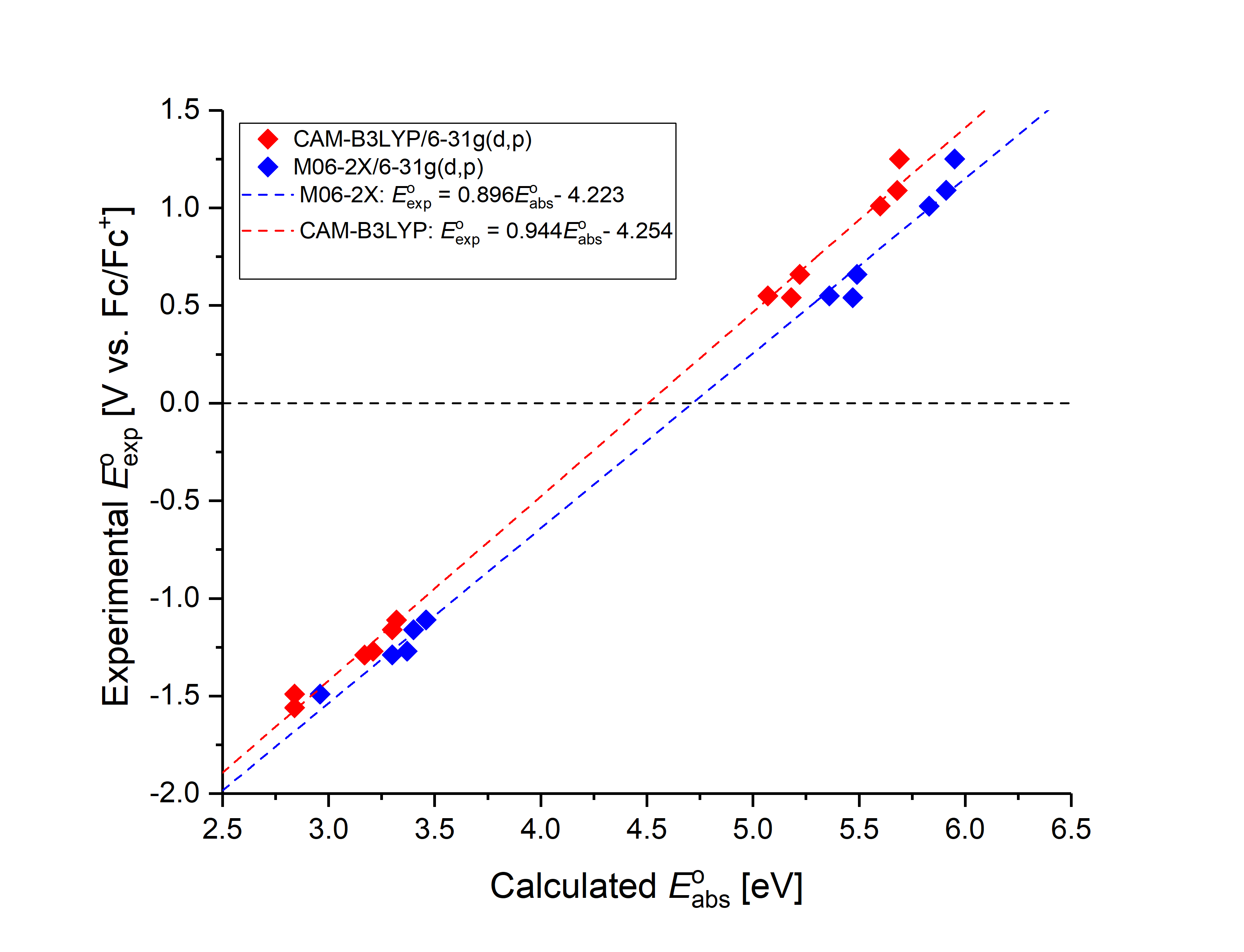


Figure S109: Linear correlation plot of experimental and calculated standard redox potentials using different functionals without diffuse methods.


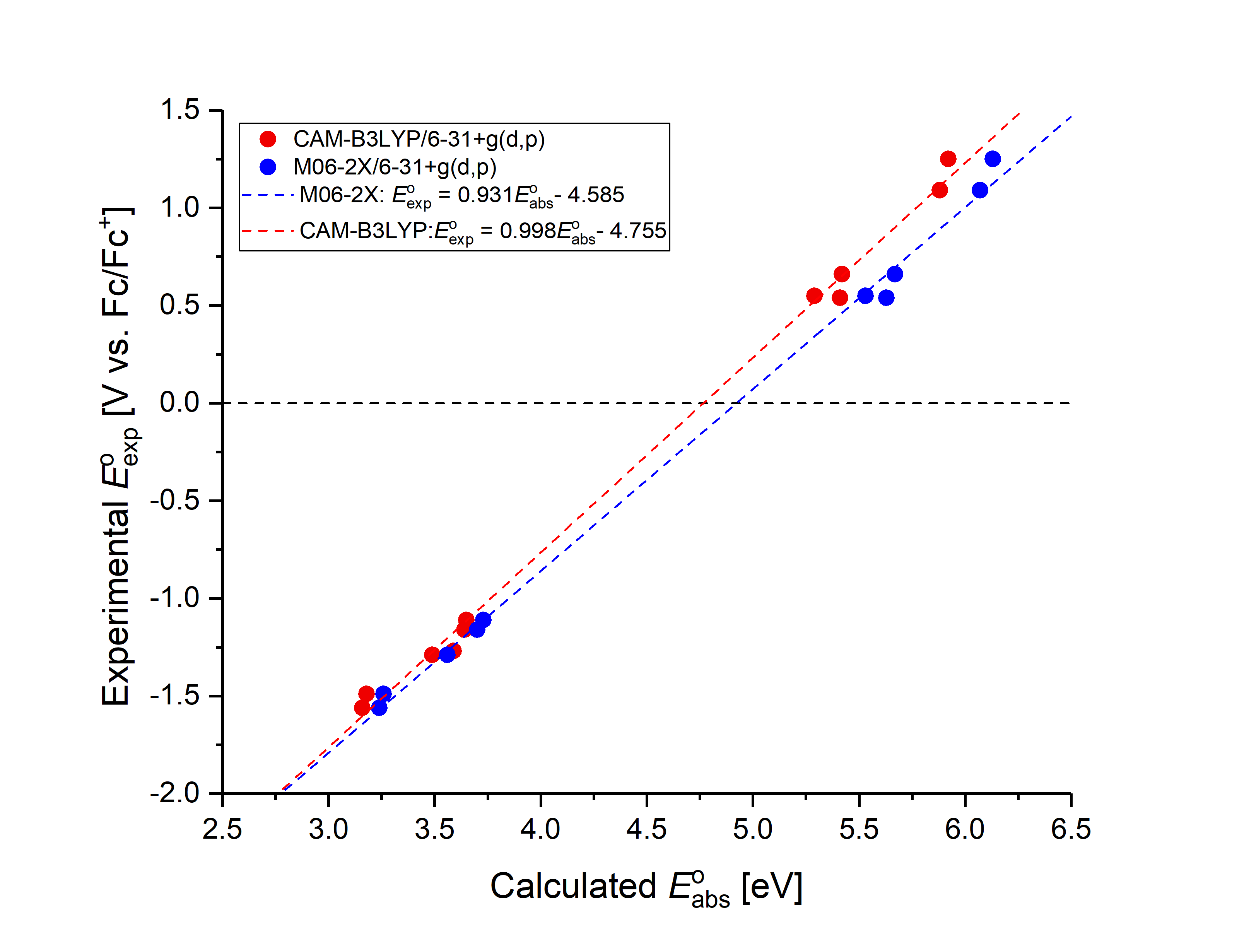


Figure S110: Linear correlation plot of experimental and calculated standard redox potentials using different functionals with diffuse methods.

**Reorganization Energy**

Internal reorganiszation energies of the electron ($\lambda_{e}$) and hole ($\lambda_{h}$) are calculated as:

$\lambda_{e}=\left[ E_{0}^{-}-E_{-} \right]+\left[ E_{-}^{0}-E_{0} \right]$ (2)

$\lambda_{h}=\left[ E_{0}^{+}-E_{+} \right]+\left[ E_{+}^{0}-E_{0} \right]$ (3)

Where, ${E_{0}, E}_{-}$ and $E_{+}$ are the electronic energy of optimized neutral, anion and cation molecules, while $E_{0}^{-}$, is the electronic energy calculated using the geometry of the neutral molecule calculated with a negative charge, $E_{0}^{+}$, is the electronic energy calculated using the geometry of the neutral molecule with a positive charge, $E_{-}^{0}$, is the electronic energy calculated using the geometry of the anionic molecule with neutral charge and $E_{+}^{0}$ is the electronic energy calculated using the geometry of the cationic molecule with neutral charge.

Table S19: Reorganization energies of **1** and **2** calculated using equation 2 and 3.

| **Reorganization Energy** | | | | |
| --- | --- | --- | --- | --- |
| **1** | M06-2X/6-31(d,p) | M06-2X/6-31+(d,p) | CAM-B3LYP/6-31(d,p) | CAM-B3LYP/6-31+(d,p) |
| $\lambda_{e}$(eV) | 0.24 | 0.23 | 0.26 | 0.26 |
| $\lambda_{h}$(eV) | 0.12 | 0.12 | 0.12 | 0.12 |
| **2** | M06-2X/6-31(d,p) | M06-2X/6-31+(d,p) | CAM-B3LYP/6-31(d,p) | CAM-B3LYP/6-31+(d,p) |
| $\lambda_{e}$(eV) | 0.28 | 0.28 | 0.42 | 0.31 |
| $\lambda_{h}$(eV) | 0.13 | 0.13 | 0.14 | 0.14 |

**Geometries**

Geometries optimizes with CAM-B3LYP/6-31+g(d,p), are given in the following section.

**1**:

B 4.72905000 0.86260900 -0.51044100

N 4.54559200 2.34009700 -0.58139400

C 3.36874700 2.91942300 -0.95108100

C 5.44156200 3.21760900 -0.05837200

C 3.61052400 4.35590900 -0.90235700

C 4.90465100 4.54195700 -0.34399600

N 5.50819000 0.65281700 0.75335800

C 6.38010200 1.57920400 1.23356100

C 5.23864200 -0.34671700 1.64135000

C 6.93751600 1.00175600 2.45011900

C 6.22560700 -0.20175500 2.70412300

N 3.36528700 0.34250300 -0.16330000

C 3.15393600 -0.64635800 0.74894300

C 2.22581900 0.97788100 -0.54852400

C 1.71739000 -0.84716600 0.78138400

C 1.13521100 0.16775100 -0.02917900

N 4.10771100 -1.05062100 1.59848200

N 6.41358500 2.83538900 0.77672300

N 2.22760900 2.23189300 -1.01805200

C 2.80472800 5.45078100 -1.20767800

C 5.39311000 5.82214700 -0.09116900

C 4.59109200 6.90512700 -0.42306500

C 3.31232400 6.72143800 -0.97451700

H 6.37444300 5.96479000 0.34780500

H 4.95443700 7.91265600 -0.25064200

H 1.81177500 5.31113600 -1.62067000

C 7.90191700 1.46046700 3.34510800

C 8.16223300 0.69589800 4.47383600

C 7.45895400 -0.49386200 4.72470600

C 6.47834000 -0.94767300 3.85339200

H 8.43543000 2.38622400 3.15966300

H 8.91907500 1.02310200 5.17882800

H 7.68537800 -1.06469200 5.61902800

H 5.92652500 -1.85899100 4.05596100

C 0.90843200 -1.74005000 1.48680400

C -0.24526100 0.29342100 -0.13339500

C -1.04836900 -0.61741400 0.55638400

C -0.46262000 -1.62737900 1.35995800

H 1.34604600 -2.50766100 2.11510500

H -0.69146400 1.07590800 -0.73634800

H -1.11317000 -2.31824200 1.88410700

O 5.31823200 0.35776300 -1.71986900

C 5.56830200 -0.98392200 -1.86691800

C 4.60723500 -1.81970800 -2.43462400

C 6.79733800 -1.51692400 -1.49597100

C 4.88121300 -3.16946600 -2.61531200

H 3.65204200 -1.40288000 -2.73743700

C 7.05742100 -2.87400200 -1.68384500

H 7.55200500 -0.86595300 -1.06644300

C 6.10949200 -3.73326900 -2.24396200

H 4.11252100 -3.79284500 -3.06105100

H 8.02730500 -3.25145100 -1.38307900

C 6.36418800 -5.22971300 -2.46559700

C 6.24539500 -5.55505500 -3.96633300

H 6.42205300 -6.62212800 -4.13707900

H 5.25268100 -5.31466800 -4.35623500

H 6.98150700 -4.99085800 -4.54735100

C 5.32001300 -6.05003100 -1.68522500

H 5.38481800 -5.84439900 -0.61223500

H 4.30096500 -5.82426100 -2.01053800

H 5.48975400 -7.12097800 -1.83774300

C 7.75890800 -5.65813900 -1.99254700

H 8.55207100 -5.13501400 -2.53523300

H 7.89882900 -5.47932500 -0.92212400

H 7.88940700 -6.72991800 -2.16885100

C -2.46985200 -0.53048700 0.45753100

C -3.67614300 -0.45598200 0.37393400

H 2.71057100 7.59055800 -1.21831000

C -5.09205700 -0.36417300 0.27110500

C -5.67643500 0.60905600 -0.57360500

C -5.91384900 -1.24547200 1.01265600

C -4.87818800 1.51066100 -1.33339300

C -7.09906100 0.69493100 -0.67249900

C -7.33470900 -1.14730100 0.90092400

C -5.35807400 -2.23397900 1.87358600

C -5.44928300 2.44978600 -2.14744200

H -3.79951200 1.44003500 -1.25596900

C -7.66738400 1.68746500 -1.53229100

C -7.91659500 -0.18106500 0.06226600

C -8.14552500 -2.05022100 1.65891800

H -4.27967000 -2.30258200 1.95655000

C -6.15808200 -3.08370100 2.58684800

C -6.85448900 2.53622500 -2.24502400

H -4.82689500 3.12888600 -2.71918400

C -7.56175100 -2.98876100 2.47589100

H -5.71859600 -3.83075600 3.23815300

H -7.31687400 3.27730200 -2.88768400

H -8.20492600 -3.65796200 3.03668900

C -9.36412500 -0.08803900 -0.04558800

C -9.96420700 0.87460800 -0.88058700

C -9.13507300 1.81396600 -1.66691400

C -11.35305300 0.95473900 -0.97739500

H -11.77820100 1.70845400 -1.63060900

C -12.16717900 0.09117300 -0.25606400

C -11.58542800 -0.86145800 0.57037300

C -10.19873600 -0.95862400 0.68221700

C -9.62108000 -1.98711300 1.57478000

H -13.24619800 0.16000300 -0.33735500

H -12.19539500 -1.54913500 1.14536400

O -10.33693300 -2.75390500 2.21737000

O -9.64532500 2.66085400 -2.39887100

**1** cation:

B 4.75081700 0.83998800 -0.56950500

N 4.54516600 2.32099700 -0.65355900

C 3.35593900 2.88753000 -1.00153000

C 5.40945100 3.22163200 -0.10898000

C 3.57025600 4.33395900 -0.96383500

C 4.85621500 4.54321500 -0.40449700

N 5.51586400 0.66639500 0.71846500

C 6.35008200 1.62441500 1.21143200

C 5.22751800 -0.29177400 1.64530700

C 6.89774500 1.08629400 2.45687800

C 6.19502000 -0.11421300 2.72831200

N 3.38189100 0.31730800 -0.20902400

C 3.16407800 -0.62845000 0.75019200

C 2.23568400 0.95357000 -0.57644300

C 1.72185800 -0.84217300 0.79043400

C 1.13665700 0.14684600 -0.03959700

N 4.10783000 -1.01416400 1.61251900

N 6.38126700 2.87113200 0.73610400

N 2.21836800 2.19271600 -1.07006000

C 2.74564300 5.40061900 -1.27842400

C 5.32791400 5.82057800 -0.15465200

C 4.50518800 6.89407600 -0.50386000

C 3.23920400 6.68810200 -1.05481900

H 6.30293800 5.98177900 0.29066600

H 4.85426800 7.90716000 -0.33816000

H 1.75494700 5.24253900 -1.68879400

C 7.83400400 1.58373700 3.34720900

C 8.08355000 0.84273300 4.50466600

C 7.39173400 -0.34004900 4.77170700

C 6.42274400 -0.82852400 3.89214700

H 8.35800200 2.51202400 3.15069500

H 8.82428500 1.19651300 5.21307600

H 7.60638400 -0.88664600 5.68318000

H 5.87234800 -1.73650000 4.11047900

C 0.93087500 -1.71657800 1.52002500

C -0.23253600 0.27426900 -0.15047400

C -1.03741500 -0.63252500 0.56485300

C -0.44933800 -1.61271000 1.38725900

H 1.37196600 -2.46244600 2.17101300

H -0.68193400 1.04231200 -0.76882600

H -1.09406300 -2.29247500 1.93196500

O 5.34633600 0.32785100 -1.75104600

C 5.59747400 -1.02289900 -1.87310200

C 4.64106300 -1.86099000 -2.44159000

C 6.81803100 -1.54836700 -1.47017100

C 4.91283500 -3.21508300 -2.59081600

H 3.69498400 -1.44539100 -2.77346300

C 7.07485000 -2.91001800 -1.62736700

H 7.56949700 -0.89149500 -1.04407400

C 6.13235100 -3.77559500 -2.18689900

H 4.15045200 -3.84384700 -3.03915500

H 8.03826900 -3.28539600 -1.30485300

C 6.38536000 -5.27648700 -2.37699400

C 6.29564400 -5.62578700 -3.87436600

H 6.47159400 -6.69602600 -4.02318100

H 5.31201400 -5.38883200 -4.28857000

H 7.04592200 -5.07443800 -4.44939800

C 5.32151800 -6.07825300 -1.60399200

H 5.36432700 -5.85370000 -0.53363800

H 4.31043000 -5.85642200 -1.95576600

H 5.49119400 -7.15203700 -1.73306600

C 7.76835400 -5.70136400 -1.86803200

H 8.57461800 -5.19232300 -2.40463600

H 7.88765400 -5.50374600 -0.79838000

H 7.89724900 -6.77658200 -2.02188400

C -2.45620200 -0.54910600 0.46057600

C -3.66137100 -0.47108300 0.37166800

H 2.62447900 7.54449300 -1.30843900

C -5.07702200 -0.37768200 0.26707000

C -5.65848400 0.65105700 -0.50987300

C -5.89625400 -1.31248200 0.94137300

C -4.86001300 1.60708900 -1.20084800

C -7.08080100 0.73983300 -0.60754600

C -7.31684400 -1.20860100 0.83412400

C -5.34008400 -2.36113500 1.72867600

C -5.43196200 2.59752100 -1.94989800

H -3.78120300 1.53704300 -1.12321900

C -7.64933800 1.78780600 -1.39860100

C -7.89804100 -0.18689900 0.06301100

C -8.12789100 -2.16323900 1.52556900

H -4.26173000 -2.43794100 1.80504900

C -6.14068900 -3.25919600 2.37791800

C -6.83779500 2.68594800 -2.04781000

H -4.81030600 3.31734500 -2.47032000

C -7.54516500 -3.15657600 2.27425000

H -5.70191800 -4.05200700 2.97317200

H -7.30023100 3.46815300 -2.63954300

H -8.18801000 -3.86413900 2.78605900

C -9.34737600 -0.08863100 -0.04132800

C -9.94703400 0.92749300 -0.80962000

C -9.11779700 1.91973600 -1.52843000

C -11.33575900 1.01278200 -0.90338400

H -11.76110600 1.80857700 -1.50441500

C -12.14962500 0.10126500 -0.24352000

C -11.56814100 -0.90472600 0.51713200

C -10.18151800 -1.00750500 0.62368600

C -9.60412000 -2.09471200 1.44450800

H -13.22856800 0.17428600 -0.32146900

H -12.17810500 -1.63041800 1.04321100

O -10.31937100 -2.90368500 2.03184300

O -9.62681100 2.81305800 -2.20217400

**1** anion:

B 4.74029500 0.85739000 -0.51124300

N 4.56718800 2.33575100 -0.58737700

C 3.39534100 2.92036100 -0.95602700

C 5.47220000 3.20986600 -0.06936600

C 3.64223900 4.35354100 -0.90583800

C 4.94021000 4.53444900 -0.35207200

N 5.51977800 0.64496100 0.75154400

C 6.40308700 1.56765900 1.22665900

C 5.24144700 -0.34524400 1.64269700

C 6.95386500 0.98993800 2.44521200

C 6.22819100 -0.20462300 2.70511400

N 3.37473000 0.34647500 -0.16442500

C 3.15362800 -0.63580400 0.75378500

C 2.23741500 0.98758500 -0.55275400

C 1.71578100 -0.83226800 0.77976300

C 1.13871100 0.17967100 -0.03704300

N 4.10391800 -1.04468900 1.60251100

N 6.44157700 2.82128800 0.76773500

N 2.24672000 2.23680500 -1.02409700

C 2.83822000 5.45260400 -1.20610700

C 5.43254600 5.81462700 -0.10009000

C 4.63238100 6.89960000 -0.42639000

C 3.34966500 6.72066800 -0.97303100

H 6.41607500 5.95437200 0.33506500

H 4.99892800 7.90597300 -0.25338100

H 1.84281500 5.31595100 -1.61428900

C 7.92271300 1.44124400 3.34001100

C 8.17299100 0.67960300 4.47258600

C 7.45591900 -0.50127600 4.72913900

C 6.47159400 -0.94770500 3.85892300

H 8.46705300 2.36004200 3.15108900

H 8.93263400 1.00205700 5.17688500

H 7.67453400 -1.06980600 5.62691100

H 5.90844300 -1.85124500 4.06534000

C 0.89732800 -1.71476600 1.48686100

C -0.23856700 0.31225700 -0.15000300

C -1.05852400 -0.59033000 0.54010700

C -0.47273200 -1.59601000 1.35301500

H 1.32675800 -2.48048700 2.12349900

H -0.67513000 1.09428000 -0.76054600

H -1.12711300 -2.28017900 1.88146800

O 5.32905900 0.34610400 -1.72064000

C 5.56646300 -0.99735300 -1.86601200

C 4.59763500 -1.82570300 -2.43185800

C 6.79085400 -1.54143400 -1.49518400

C 4.85937700 -3.17804100 -2.61108200

H 3.64559700 -1.40121000 -2.73380600

C 7.03882700 -2.90100900 -1.68166000

H 7.55127100 -0.89668900 -1.06634000

C 6.08288200 -3.75242100 -2.24011300

H 4.08440200 -3.79497600 -3.05491800

H 8.00539900 -3.28683000 -1.38067800

C 6.32380400 -5.25139400 -2.46012800

C 6.20150100 -5.57739900 -3.96047300

H 6.36741200 -6.64642400 -4.13006700

H 5.21117600 -5.32753200 -4.35041100

H 6.94300200 -5.02115800 -4.54238400

C 5.27258200 -6.06141600 -1.67846300

H 5.33949300 -5.85503000 -0.60576500

H 4.25547200 -5.82676400 -2.00337200

H 5.43244900 -7.13409100 -1.82984700

C 7.71480600 -5.69193900 -1.98717200

H 8.51245800 -5.17626600 -2.53048600

H 7.85663300 -5.51342300 -0.91693900

H 7.83571800 -6.76503400 -2.16255800

C -2.47426100 -0.50174700 0.43546300

C -3.68689500 -0.42799100 0.35135300

H 2.74912400 7.59202400 -1.21193600

C -5.09036100 -0.34378000 0.25570000

C -5.68986300 0.66769000 -0.56634700

C -5.91023900 -1.26992300 0.98251800

C -4.91519700 1.59156200 -1.29141000

C -7.11800400 0.74467000 -0.65641800

C -7.33652400 -1.17588200 0.88035500

C -5.35487800 -2.27331800 1.79749300

C -5.50264800 2.56797000 -2.08587300

H -3.83462900 1.53258300 -1.22208800

C -7.69871900 1.75076400 -1.47516600

C -7.92791400 -0.17372900 0.06474900

C -8.13685700 -2.09863400 1.60670900

H -4.27542800 -2.34339500 1.87394700

C -6.15512200 -3.16622800 2.49894600

C -6.88614900 2.64764000 -2.17793500

H -4.87727400 3.26618100 -2.63299900

C -7.53804500 -3.08000700 2.40482600

H -5.69593700 -3.92937600 3.11921900

H -7.36530500 3.40140500 -2.79226000

H -8.18175400 -3.76598000 2.94350000

C -9.34393300 -0.08972400 -0.03138700

C -9.96307000 0.90220300 -0.84114900

C -9.15478100 1.86685400 -1.59875700

C -11.35351900 0.96751100 -0.92348300

H -11.78831600 1.73769600 -1.55150900

C -12.16034500 0.07618300 -0.22512100

C -11.56621000 -0.89730000 0.57015400

C -10.17936100 -0.99525900 0.67943100

C -9.59970400 -2.04008200 1.53389800

H -13.24072800 0.13949000 -0.29995100

H -12.17050000 -1.60621500 1.12608400

O -10.31905500 -2.83716800 2.16568000

O -9.68428800 2.74069800 -2.31139700

**2**:

B -2.38572400 -0.00625000 -0.17972700

N -3.79251800 -0.04098400 -0.66076300

C -4.51075600 -1.19511400 -0.75352900

C -4.55684600 1.08011400 -0.78480200

C -5.82161100 -0.80126500 -1.24749900

C -5.85042500 0.62004700 -1.26710900

N -2.32555800 1.18304700 0.72453900

C -3.13882500 2.26593700 0.56480700

C -1.66393400 1.18056500 1.91644900

C -2.78579400 3.18396200 1.63649200

C -1.86073200 2.50817500 2.47788800

N -2.27792800 -1.16674200 0.75661300

C -1.61735400 -1.10503700 1.94750200

C -3.04650900 -2.28563300 0.62693000

C -1.76012700 -2.42388800 2.54476100

C -2.65648600 -3.15934900 1.72254000

N -1.23273400 0.05364300 2.48989400

N -4.19849500 2.25031500 -0.24859400

N -4.10570800 -2.33484100 -0.18571900

C -6.95676200 -1.54025000 -1.57777500

C -7.01449000 1.30286200 -1.61709700

C -8.12927700 0.55591900 -1.96844600

C -8.10076800 -0.84922200 -1.94899900

H -7.04263900 2.38693400 -1.61855600

H -9.04284200 1.06322500 -2.25986900

H -6.94070800 -2.62419200 -1.54919800

C -3.24689200 4.45819300 1.96525500

C -2.75637700 5.05284700 3.11828100

C -1.83962000 4.38550600 3.94899200

C -1.39374500 3.10718000 3.64708600

H -3.96426200 4.96899500 1.33231900

H -3.08738200 6.05008900 3.38822500

H -1.47930300 4.88001800 4.84479400

H -0.69808100 2.58773800 4.29681800

C -1.26999100 -2.97125400 3.72966900

C -3.06538100 -4.44193900 2.08596300

C -2.55190900 -4.98448700 3.25462000

C -1.66375300 -4.25794600 4.06651200

H -0.59662100 -2.40629700 4.36476200

H -3.76097900 -4.99858500 1.46754700

H -1.28440600 -4.71276200 4.97540700

O -1.46002200 -0.00340700 -1.30981300

H -8.99283300 -1.40108700 -2.22566500

C -0.11627300 0.00408600 -1.14683600

C 0.58842300 -1.21791300 -1.09154300

C 0.57610400 1.23301100 -1.09168900

C -0.05711300 -2.46653000 -1.32611400

C 1.99059700 -1.19670700 -0.83462400

C 1.97817400 1.22538600 -0.83380800

C -0.08089300 2.47531900 -1.32744000

C 0.61871600 -3.66186500 -1.28634400

H -1.10915400 -2.43803200 -1.57407300

C 2.67686400 -2.44555900 -0.74242500

C 2.67128300 0.01797100 -0.68072100

C 2.65272500 2.48025000 -0.73973400

H -1.13234400 2.43689000 -1.57649700

C 0.58383500 3.67689900 -1.28706200

C 2.00084800 -3.62083400 -0.95951700

C 1.96562400 3.64909100 -0.95729800

H 2.57330200 -4.53896400 -0.88783600

H 2.52897000 4.57272800 -0.88395900

C 4.09161300 0.02607700 -0.40607900

C 4.80875900 -1.17901100 -0.27047200

C 4.12396300 -2.48254100 -0.42878100

C 6.17297600 -1.15380200 0.00304300

H 6.67217100 -2.11201100 0.09607700

C 6.88506500 0.04054300 0.15143100

C 6.16566900 1.22496400 0.00738500

C 4.79608000 1.23421100 -0.26788100

C 4.09918200 2.53050400 -0.42390400

H 6.64964200 2.18853900 0.10268700

O 4.69583900 3.60190300 -0.30096200

O 4.73171500 -3.54748100 -0.30862400

H -2.84213500 -5.98664800 3.55186200

C -0.08063100 5.02433400 -1.58525300

C 0.66792900 5.71627000 -2.74046300

H 0.20021500 6.68048000 -2.96269900

H 1.71650100 5.90619600 -2.49683100

H 0.63633200 5.10582000 -3.64816900

C -0.01294400 5.91230300 -0.32847800

H -0.46918700 6.88608600 -0.53321400

H -0.55160200 5.45231800 0.50533900

H 1.01811600 6.08915500 -0.01003400

C -1.54941500 4.86876700 -1.99493200

H -1.65722200 4.25597000 -2.89515500

H -2.15244900 4.42204800 -1.20006700

H -1.97099700 5.85417600 -2.21255600

C -0.03331900 -5.01560000 -1.58336000

C 0.71375400 -5.69514700 -2.74686600

H 1.76658900 -5.87278000 -2.51237000

H 0.25597600 -6.66436800 -2.96804800

H 0.66716400 -5.08250600 -3.65244000

C -1.50711300 -4.87506700 -1.98023400

H -2.10828100 -4.43561400 -1.17985900

H -1.62915400 -4.26234600 -2.87869300

H -1.91990300 -5.86462800 -2.19591200

C 0.05442600 -5.90724500 -0.33044400

H -0.48081600 -5.45537600 0.50994900

H -0.39440500 -6.88456300 -0.53465800

H 1.08989000 -6.07571400 -0.02196100

C 8.38736500 0.00454700 0.45273600

C 8.98418300 1.41104400 0.58207600

H 8.52232400 1.97676900 1.39684300

H 10.05336200 1.33237300 0.79856000

H 8.87535500 1.98717800 -0.34172100

C 8.62384800 -0.74627300 1.77647200

H 8.25667200 -1.77513900 1.73208700

H 9.69468500 -0.78380800 2.00034900

H 8.11991300 -0.24275800 2.60718700

C 9.11808300 -0.72973500 -0.68695600

H 8.76491000 -1.75817700 -0.80050400

H 8.97185100 -0.21447100 -1.64125500

H 10.19256500 -0.76686300 -0.48122800

**2** cation:

B -2.35612200 -0.00385000 -0.23146300

N -3.76988800 -0.13984400 -0.70167000

C -4.43973500 -1.32837400 -0.70489900

C -4.61278700 0.92286100 -0.84460400

C -5.78056800 -1.03831700 -1.20897600

C -5.88889000 0.37224300 -1.29653700

N -2.38280700 1.23127000 0.63082700

C -3.28113400 2.24211300 0.44427500

C -1.76729400 1.31462600 1.84680100

C -3.00485500 3.23189500 1.48314900

C -2.05176400 2.65382800 2.35811700

N -2.20503800 -1.10904300 0.77869200

C -1.59438100 -0.94683300 1.98841200

C -2.93505900 -2.26117300 0.72552700

C -1.67603400 -2.23981800 2.66390600

C -2.52026400 -3.06102300 1.87576100

N -1.27314600 0.25051900 2.48046700

N -4.33057700 2.13777500 -0.37187900

N -3.98061500 -2.41848900 -0.08770100

C -6.86229300 -1.85721700 -1.48614600

C -7.07983200 0.97698500 -1.66155500

C -8.15801500 0.14564900 -1.97262800

C -8.05131900 -1.24391500 -1.88672800

H -7.17018800 2.05605300 -1.70930100

H -9.09881300 0.58822200 -2.28025100

H -6.78750100 -2.93517000 -1.40041900

C -3.55570100 4.47526400 1.74520900

C -3.11162700 5.15185300 2.88306900

C -2.17067800 4.58336600 3.74411600

C -1.63820900 3.31504200 3.50249000

H -4.29955400 4.91002500 1.08738800

H -3.50950000 6.13555500 3.10569100

H -1.85256900 5.13494300 4.62172600

H -0.92352200 2.86701800 4.18319400

C -1.17843200 -2.68180500 3.87823400

C -2.87663200 -4.33175800 2.29569200

C -2.34645200 -4.78515200 3.50536700

C -1.51279300 -3.97662400 4.28070700

H -0.54832400 -2.04777600 4.49126400

H -3.53879500 -4.95364300 1.70429300

H -1.12314400 -4.35951800 5.21726300

O -1.44157500 -0.00703200 -1.33984800

H -8.91103900 -1.85860800 -2.12892100

C -0.08879700 0.00400500 -1.16489700

C 0.60942800 -1.21762600 -1.10050900

C 0.59639700 1.23319500 -1.10614900

C -0.03231000 -2.46875700 -1.34219200

C 2.00904000 -1.19571600 -0.82841600

C 1.99617200 1.22610200 -0.83313400

C -0.05718300 2.47707600 -1.35283600

C 0.64317400 -3.66237300 -1.29111200

H -1.07803500 -2.44329500 -1.61606400

C 2.69416700 -2.44482300 -0.72524300

C 2.68764200 0.01923500 -0.67096100

C 2.66939300 2.48159000 -0.73318700

H -1.10329300 2.44050400 -1.62374200

C 0.60694500 3.67723200 -1.30403800

C 2.02282200 -3.62024000 -0.94581300

C 1.98642600 3.64973300 -0.95736800

H 2.59459700 -4.53792900 -0.86549600

H 2.54879100 4.57337000 -0.87848600

C 4.10859300 0.02807300 -0.38494400

C 4.82443800 -1.17582200 -0.24217900

C 4.13991700 -2.48010400 -0.39698600

C 6.18778800 -1.15059700 0.03564500

H 6.68664100 -2.10854200 0.13227400

C 6.89952000 0.04412900 0.18243400

C 6.18006800 1.22808300 0.03515200

C 4.81144500 1.23571600 -0.24476800

C 4.11444600 2.53192200 -0.40326300

H 6.66339800 2.19194000 0.13031400

O 4.70653000 3.60298800 -0.27103400

O 4.74320800 -3.54412800 -0.26261300

H -2.59146900 -5.78266700 3.85260800

C -0.05186600 5.02459200 -1.61281000

C 0.70935500 5.71077500 -2.76321900

H 0.24636300 6.67545100 -2.99223900

H 1.75599800 5.89866200 -2.51051700

H 0.68423000 5.09773300 -3.66922200

C 0.00569600 5.91593100 -0.35778700

H -0.44893400 6.88879500 -0.56913400

H -0.53950700 5.45849600 0.47357400

H 1.03372100 6.09440100 -0.03114000

C -1.51681300 4.87107400 -2.03589500

H -1.61931200 4.25328600 -2.93311000

H -2.12799400 4.43308700 -1.24203000

H -1.93404600 5.85608400 -2.26229100

C -0.00172200 -5.01654100 -1.59981700

C 0.76006900 -5.68789800 -2.75869000

H 1.81112600 -5.86194900 -2.51437700

H 0.30873600 -6.65807300 -2.98780900

H 0.71958900 -5.07191800 -3.66213500

C -1.47182500 -4.88033200 -2.01162100

H -2.08542900 -4.45076500 -1.21482000

H -1.58778200 -4.26314100 -2.90769500

H -1.87729500 -5.87038700 -2.23758400

C 0.07681700 -5.91295500 -0.34973800

H -0.46554100 -5.46499600 0.48841400

H -0.36943000 -6.88953300 -0.56178400

H 1.10942600 -6.08247000 -0.03299000

C 8.40161900 0.00922800 0.48365200

C 8.99668500 1.41616000 0.61576700

H 8.53377600 1.98003600 1.43123600

H 10.06563900 1.33798800 0.83300200

H 8.88855700 1.99368700 -0.30720200

C 8.63911300 -0.74441700 1.80557800

H 8.27400800 -1.77392700 1.75918400

H 9.71002700 -0.78065900 2.02863800

H 8.13514400 -0.24329200 2.63772900

C 9.13162200 -0.72182700 -0.65864400

H 8.77995000 -1.75059800 -0.77378300

H 8.98365200 -0.20474000 -1.61164500

H 10.20624800 -0.75764100 -0.45409900

**2** anion:

B -2.37212700 -0.00731500 -0.20475300

N -3.79884800 -0.07709000 -0.64569200

C -4.49505700 -1.24503600 -0.70547700

C -4.58532800 1.02581000 -0.77808500

C -5.82305100 -0.88652800 -1.17992300

C -5.87947700 0.53350500 -1.22561500

N -2.33582400 1.19783700 0.69345700

C -3.16860600 2.26262200 0.52530600

C -1.65539100 1.23080500 1.87088300

C -2.81713600 3.20856500 1.57481400

C -1.86704100 2.56559400 2.41282500

N -2.24184500 -1.14584900 0.76752700

C -1.56418200 -1.05007200 1.94336800

C -2.98762500 -2.28132500 0.66941800

C -1.67092500 -2.36068800 2.56904000

C -2.56445300 -3.12854600 1.77508100

N -1.19270700 0.12454100 2.46063700

N -4.24086900 2.21389300 -0.26941300

N -4.05881400 -2.36754300 -0.12338300

C -6.95065900 -1.65249100 -1.47424500

C -7.06361200 1.18685800 -1.56595000

C -8.17069100 0.41305100 -1.88121700

C -8.11488300 -0.99100100 -1.83582700

H -7.11232000 2.27009400 -1.58778000

H -9.09917800 0.89758300 -2.16451600

H -6.91287800 -2.73529600 -1.42601200

C -3.29582600 4.47973200 1.88810200

C -2.79726900 5.10503000 3.02195200

C -1.85448900 4.47040900 3.84847900

C -1.39092800 3.19435500 3.56188000

H -4.03185500 4.96645000 1.25745000

H -3.14118800 6.10157600 3.27841100

H -1.48635900 4.98849500 4.72771000

H -0.67313900 2.69984200 4.20703900

C -1.15091900 -2.87603000 3.75495400

C -2.94200600 -4.41132600 2.16851500

C -2.39950700 -4.92258100 3.33882400

C -1.51312500 -4.16420400 4.12224600

H -0.47721400 -2.28663100 4.36714700

H -3.63550800 -4.99316400 1.57109800

H -1.10846400 -4.59481500 5.03211700

O -1.48168000 -0.00858500 -1.33575700

H -9.00128200 -1.56496600 -2.08478100

C -0.11667000 0.00151200 -1.14790300

C 0.58100000 -1.23391200 -1.10485000

C 0.56674800 1.24415000 -1.10654900

C -0.05441400 -2.47356200 -1.34312500

C 1.98952400 -1.21704700 -0.84054600

C 1.97561400 1.24289500 -0.83996900

C -0.08122700 2.47659700 -1.34865600

C 0.63287100 -3.68672700 -1.30117900

H -1.10738300 -2.45006400 -1.58755900

C 2.67596700 -2.45283800 -0.75276500

C 2.66592000 0.01728400 -0.67887200

C 2.64897800 2.48597100 -0.74955800

H -1.13298600 2.44136200 -1.59639300

C 0.59319600 3.69721500 -1.30449100

C 1.99249700 -3.65514800 -0.98074900

C 1.95144600 3.68111400 -0.97839200

H 2.57199000 -4.56851200 -0.91481700

H 2.52017000 4.60091100 -0.90895400

C 4.05597300 0.02667800 -0.40765500

C 4.78030700 -1.18876100 -0.27139600

C 4.10693600 -2.48648000 -0.43956300

C 6.14319600 -1.15263300 0.00778600

H 6.64608200 -2.10947000 0.10175300

C 6.85340000 0.04409000 0.15984600

C 6.13419500 1.22894400 0.01128700

C 4.76495300 1.24745000 -0.26920600

C 4.07814300 2.53544800 -0.43443300

H 6.61956900 2.19231900 0.10619600

O 4.68840000 3.62150400 -0.31708700

O 4.72982900 -3.56329100 -0.32518300

H -2.66444000 -5.92506600 3.65819100

C -0.09555300 5.03753600 -1.60149800

C 0.64143600 5.75343800 -2.74912200

H 0.15748900 6.71109700 -2.96968800

H 1.68541000 5.95682000 -2.49793800

H 0.62651600 5.14688700 -3.66023700

C -0.05226200 5.92527600 -0.34365100

H -0.53148600 6.89026600 -0.54275900

H -0.57917100 5.44852900 0.48850400

H 0.97533500 6.11984200 -0.02494100

C -1.56123600 4.86600100 -2.01965500

H -1.65693300 4.25272900 -2.92095500

H -2.16210800 4.40818100 -1.22949700

H -1.99488800 5.84702400 -2.23717500

C -0.04060000 -5.03444400 -1.59914100

C 0.68636900 -5.72459400 -2.76881200

H 1.73833300 -5.91198500 -2.53910800

H 0.21512100 -6.68810300 -2.99143100

H 0.64384300 -5.10812500 -3.67235700

C -1.51672000 -4.88167700 -1.98640300

H -2.10837000 -4.43678900 -1.18189000

H -1.63977500 -4.26474800 -2.88187400

H -1.94027100 -5.86760400 -2.20164600

C 0.04095800 -5.93626400 -0.35334800

H -0.47641500 -5.47764100 0.49477200

H -0.42741800 -6.90592600 -0.55528500

H 1.07710400 -6.11960200 -0.05660000

C 8.35535600 0.00806900 0.46919400

C 8.95477900 1.41407600 0.59495000

H 8.48916700 1.98423100 1.40453500

H 10.02367200 1.33619900 0.81573500

H 8.84789900 1.98663100 -0.33136100

C 8.58891400 -0.73529200 1.79772200

H 8.21669100 -1.76258500 1.75739800

H 9.65916000 -0.77579800 2.02698300

H 8.08246800 -0.22593200 2.62355500

C 9.09663700 -0.73166000 -0.66017400

H 8.73969900 -1.75884700 -0.77392200

H 8.95916500 -0.21936400 -1.61755000

H 10.17006800 -0.77230500 -0.44635100

**4**:

C -2.23879000 0.02266100 0.03564000

C -1.02233600 0.02317400 0.02663200

C 0.40540600 0.01733300 0.01548000

C 1.11822300 1.23805700 0.00950200

C 1.10520600 -1.21102600 0.00924700

C 0.44411400 2.49272600 0.00518300

C 2.54706900 1.22260500 0.00671100

C 2.53413200 -1.21063900 0.00657900

C 0.41751400 -2.45849800 0.00470900

C 1.13853500 3.67074700 0.00088000

H -0.63947900 2.49675800 0.00273200

C 3.24618100 2.47116900 0.00384700

C 3.24358200 0.00226600 0.00646100

C 3.22004700 -2.46647400 0.00382700

H -0.66620800 -2.45143900 0.00244600

C 1.09982500 -3.64364000 0.00057600

C 2.55003500 3.65621300 0.00141900

H 0.60988400 4.61729700 -0.00330100

C 2.51140600 -3.64407500 0.00137900

H 0.56132600 -4.58463900 -0.00357100

H 3.10959200 4.58515000 -0.00103800

H 3.06121100 -4.57883000 -0.00091600

C 4.69837900 -0.00541300 0.00466400

C 5.42529200 1.20093100 0.00357600

C 4.72511800 2.50420100 0.00315600

C 6.81973900 1.18219600 0.00242300

H 7.34476900 2.13082600 0.00169500

C 7.51465600 -0.02031000 0.00207300

C 6.80704800 -1.21539700 0.00256500

C 5.41248400 -1.21936900 0.00367700

C 4.69853400 -2.51515700 0.00331700

H 8.59892000 -0.02603600 0.00123500

H 7.32198100 -2.16954500 0.00196100

O 5.30907900 -3.58310900 0.00261300

O 5.34695600 3.56561700 0.00216500

Si -4.09916500 0.00353200 0.00442500

C -4.64052600 1.82060400 0.43366900

C -3.81537400 2.37702100 1.61181700

C -4.39298800 2.77123600 -0.75302500

C -6.13375900 1.90465600 0.80357800

H -3.96240300 1.82352900 2.53917400

H -2.74432200 2.38043900 1.39198800

H -4.11685500 3.41532200 1.80046000

H -5.02944800 2.54985800 -1.61210300

H -4.61980300 3.79855000 -0.44079500

H -3.35078500 2.75496600 -1.08656500

H -6.40317400 2.95168200 0.99270100

H -6.78555200 1.54178700 0.00412900

H -6.37183900 1.34347500 1.71030100

C -4.55618900 -0.53299500 -1.80925600

C -3.66838600 0.19512700 -2.83903900

C -4.31243400 -2.03881200 -2.02413500

C -6.03112700 -0.23786600 -2.14123000

H -3.79271900 1.27805700 -2.82544600

H -2.60833900 -0.02329600 -2.68372600

H -3.93366600 -0.15074300 -3.84632300

H -4.97799800 -2.66629500 -1.42805600

H -4.49773200 -2.28306800 -3.07785600

H -3.27976200 -2.32478600 -1.80157000

H -6.25596500 -0.60043800 -3.15249000

H -6.72379700 -0.73547900 -1.45691700

H -6.25597800 0.83127700 -2.12653500

C -4.62596300 -1.29759700 1.35084600

C -3.72829300 -2.54976100 1.28519600

C -4.46453200 -0.73219300 2.77467100

C -6.09295600 -1.73636300 1.18044300

H -3.77552500 -3.06643400 0.32625800

H -2.68206700 -2.30234300 1.48491400

H -4.05049700 -3.26155900 2.05593200

H -5.15268500 0.08887500 2.98512800

H -4.68086400 -1.52680100 3.50002400

H -3.44514500 -0.38452600 2.96791700

H -6.36067200 -2.43046000 1.98737400

H -6.79071600 -0.89584900 1.23145500

H -6.26687500 -2.25882100 0.23687800

**4** cation:

C -2.23320000 0.06269800 0.04818600

C -1.01204300 0.06417100 0.04432600

C 0.39245300 0.04799600 0.03304600

C 1.12450000 1.27695500 0.02591800

C 1.08899600 -1.20188800 0.02478200

C 0.45692000 2.51715500 0.02581900

C 2.54696100 1.24960500 0.01779700

C 2.51167300 -1.21531500 0.01739200

C 0.38604300 -2.42264800 0.02321800

C 1.16443000 3.70263500 0.01971300

H -0.62594200 2.53199200 0.02813900

C 3.24891400 2.47478900 0.01183000

C 3.24631400 0.00689700 0.01382100

C 3.17853600 -2.46001700 0.01160300

H -0.69699900 -2.40725500 0.02465400

C 1.05984900 -3.62763100 0.01717100

C 2.56090000 3.67908100 0.01330000

H 0.63754100 4.64947700 0.01938600

C 2.45640900 -3.64416300 0.01214500

H 0.50607100 -4.55902600 0.01583500

H 3.13199800 4.60041000 0.00841100

H 3.00057600 -4.58166900 0.00748200

C 4.68982500 -0.01362100 0.00424000

C 5.42701900 1.18846400 -0.00109400

C 4.73263100 2.49565200 0.00278400

C 6.81740600 1.15971600 -0.01044000

H 7.35524000 2.10066100 -0.01437700

C 7.49732700 -0.05362600 -0.01473100

C 6.78309200 -1.24708600 -0.00972800

C 5.39243800 -1.23619800 -0.00044900

C 4.66109500 -2.52310000 0.00381100

H 8.58115800 -0.06902600 -0.02202800

H 7.29383600 -2.20300500 -0.01311600

O 5.24864300 -3.59596600 0.00062300

O 5.35032800 3.55143800 -0.00153800

Si -4.11241100 0.00943800 0.00202300

C -4.67300300 1.84183600 0.29793200

C -3.86994100 2.48733900 1.44565800

C -4.43190200 2.71371900 -0.94902300

C -6.17182500 1.92037700 0.64859200

H -4.02153000 1.99610100 2.40647500

H -2.79576200 2.49596500 1.23996300

H -4.18864800 3.53070900 1.55966200

H -5.05411000 2.42035400 -1.79665200

H -4.68561900 3.75344200 -0.70867700

H -3.38630400 2.70126800 -1.27192700

H -6.45924400 2.97203100 0.77003900

H -6.80903300 1.49913000 -0.13362600

H -6.40976700 1.41228100 1.58587700

C -4.49148000 -0.66403300 -1.78153100

C -3.59981800 0.02111200 -2.83749100

C -4.20605400 -2.17367000 -1.88902100

C -5.96685500 -0.42861200 -2.15904800

H -3.74516300 1.09986200 -2.89160700

H -2.53765600 -0.16838300 -2.65731600

H -3.84192600 -0.38976200 -3.82538700

H -4.86466300 -2.77745800 -1.26199700

H -4.36983500 -2.49080800 -2.92624600

H -3.16974800 -2.41795400 -1.63608900

H -6.15671400 -0.85529000 -3.15170200

H -6.66100500 -0.90695400 -1.46286300

H -6.22138500 0.63240200 -2.21178500

C -4.59792300 -1.21092200 1.43269600

C -3.66441200 -2.43787800 1.46046800

C -4.47438500 -0.54490900 2.81620100

C -6.05039500 -1.70031900 1.26992600

H -3.67103900 -3.01006200 0.53264700

H -2.63106300 -2.15156700 1.67650200

H -3.98910700 -3.11267900 2.26196500

H -5.18802200 0.26832000 2.96078600

H -4.68197500 -1.29521300 3.58897100

H -3.46798300 -0.15773500 3.00237300

H -6.30973300 -2.34618600 2.11784200

H -6.77159600 -0.87849900 1.25704700

H -6.19581700 -2.28914500 0.36144100

**4** anion:

C -2.23458300 0.00661800 0.04246400

C -1.01313800 0.00746900 0.03326500

C 0.40465200 0.00572600 0.02262100

C 1.11719100 1.24786800 0.01565600

C 1.11250300 -1.23913600 0.01629700

C 0.45080600 2.48783300 0.01427000

C 2.55126700 1.23661200 0.00996500

C 2.54656100 -1.23334900 0.01025700

C 0.44110600 -2.47649000 0.01588400

C 1.14968200 3.68892300 0.00927800

H -0.63353700 2.49418000 0.01572000

C 3.24621400 2.47581700 0.00574300

C 3.25201900 0.00030300 0.00794500

C 3.23674900 -2.47518500 0.00618100

H -0.64322800 -2.47886100 0.01842900

C 1.13559700 -3.68017900 0.01114700

C 2.53809500 3.68471200 0.00581000

H 0.60611700 4.62848900 0.00818500

C 2.52400800 -3.68136700 0.00697700

H 0.58847200 -4.61769300 0.01085700

H 3.10221000 4.61045200 0.00258100

H 3.08465700 -4.60922700 0.00385900

C 4.67301600 -0.00241200 0.00253400

C 5.40524400 1.21728200 -0.00030800

C 4.71082300 2.51161300 0.00106800

C 6.79954900 1.19295300 -0.00490200

H 7.32326900 2.14296900 -0.00678100

C 7.50046500 -0.00789100 -0.00711700

C 6.79490700 -1.20602000 -0.00500900

C 5.40053600 -1.22492100 -0.00042100

C 4.70116000 -2.51658700 0.00095000

H 8.58532500 -0.00998500 -0.01059000

H 7.31487800 -2.15809800 -0.00695800

O 5.32706800 -3.59470300 -0.00225400

O 5.34085900 3.58732400 -0.00178900

Si -4.07935200 0.00112400 0.00269900

C -4.62542300 1.80443100 0.49674500

C -3.79798700 2.31784500 1.69250900

C -4.37377400 2.79573800 -0.65523500

C -6.11778200 1.88370800 0.86973200

H -3.94570500 1.73177600 2.59978000

H -2.72756400 2.32299900 1.47076900

H -4.09605400 3.35018800 1.91831900

H -5.01091300 2.60742200 -1.52185300

H -4.59521400 3.81324300 -0.30760200

H -3.33157600 2.78463100 -0.98860200

H -6.38375900 2.92544500 1.09243700

H -6.77158400 1.54829400 0.05987200

H -6.35772000 1.29470500 1.75830200

C -4.56576700 -0.46381100 -1.82695000

C -3.67628000 0.29099500 -2.83548700

C -4.33598200 -1.96279000 -2.09760900

C -6.03888900 -0.14533900 -2.14336800

H -3.79276100 1.37391300 -2.78513100

H -2.61789000 0.06118900 -2.68699400

H -3.94570200 -0.01927100 -3.85369600

H -5.00561700 -2.60563400 -1.52252100

H -4.52518800 -2.16825000 -3.15930300

H -3.30538600 -2.26476500 -1.88715200

H -6.27210400 -0.47198200 -3.16539200

H -6.73348200 -0.65847600 -1.47244900

H -6.25400600 0.92482900 -2.09209400

C -4.64188700 -1.34163100 1.29729800

C -3.76160200 -2.60316500 1.19043900

C -4.47477300 -0.82953700 2.74036400

C -6.11376700 -1.75542900 1.11129200

H -3.82117900 -3.08993100 0.21646800

H -2.71109200 -2.37421400 1.38882500

H -4.08857500 -3.33401500 1.94173700

H -5.15231500 -0.00710400 2.97896400

H -4.70084400 -1.64592200 3.43865700

H -3.45114600 -0.50095600 2.94351900

H -6.39346800 -2.47226900 1.89451600

H -6.80055300 -0.90771200 1.18759300

H -6.29234500 -2.24431200 0.15058900

**5**:

C -3.05952100 0.05030300 0.03690200

C -1.84239900 0.04602300 0.04903600

C -0.41386300 0.02877200 0.04910100

C 0.31142800 1.24547000 0.04191400

C 0.27702100 -1.20811200 0.04418400

C -0.34256800 2.50846000 0.04925600

C 1.73798700 1.21301300 0.02758200

C 1.70379100 -1.21483200 0.02917600

C -0.41153100 -2.45282700 0.05346400

C 0.33912300 3.70308600 0.04076200

H -1.42607300 2.50232500 0.06196300

C 2.44249300 2.45350600 0.01814800

C 2.42231300 -0.01085600 0.01916600

C 2.37461200 -2.47348900 0.02073700

H -1.49464800 -2.41783900 0.06808000

C 0.23811600 -3.66540400 0.04456800

C 1.75546400 3.64434100 0.02478500

C 1.65540600 -3.64518000 0.02814900

H 2.34579400 4.55148500 0.01648500

H 2.22124900 -4.56775900 0.02033400

C 3.87295100 -0.03198700 0.00250200

C 4.61337900 1.16469300 -0.00692700

C 3.92304500 2.47544600 0.00039900

C 6.00396000 1.12843900 -0.02360100

H 6.51916300 2.08253100 -0.03023900

C 6.72025200 -0.07253600 -0.03183000

C 5.97567900 -1.25010800 -0.02235800

C 4.57942600 -1.24487200 -0.00567400

C 3.85406900 -2.53563500 0.00270300

H 6.45885500 -2.21864700 -0.02782100

O 4.45551900 -3.60987800 -0.00517500

O 4.55401100 3.53216000 -0.00843800

Si -4.91915500 0.01719400 -0.02865000

C -5.48574100 1.85618500 0.24592000

C -4.68369400 2.51107900 1.38816900

C -5.22864800 2.71171500 -1.00894500

C -6.98486400 1.95570200 0.58668900

H -4.84612800 2.03531900 2.35528700

H -3.60934700 2.49738100 1.18693200

H -4.98864900 3.56090700 1.48499500

H -5.85175600 2.41743000 -1.85579700

H -5.46617000 3.75896500 -0.78275300

H -4.18228100 2.67641000 -1.32700000

H -7.26501300 3.01206000 0.68817200

H -7.62195700 1.52482800 -0.19042700

H -7.23185100 1.46612200 1.53164400

C -5.34398700 -0.66314200 -1.80205000

C -4.44354700 -0.01117500 -2.87120700

C -5.08855600 -2.17911300 -1.89574500

C -6.81505000 -0.40630400 -2.18066200

H -4.57673500 1.06822400 -2.94606400

H -3.38479000 -0.20658900 -2.68051100

H -4.68798200 -0.43835400 -3.85226200

H -5.75774400 -2.76370000 -1.26136000

H -5.25709100 -2.50628100 -2.92961600

H -4.05727100 -2.43850700 -1.63838400

H -7.01932900 -0.84725300 -3.16480700

H -7.51637800 -0.85583900 -1.47230700

H -7.04806300 0.65873700 -2.25212300

C -5.46439700 -1.17712300 1.40656600

C -4.55560300 -2.42131800 1.45531700

C -5.33266200 -0.49987000 2.78386500

C -6.92460500 -1.64109400 1.24600700

H -4.58019700 -3.01175400 0.53893700

H -3.51594500 -2.14611300 1.65255600

H -4.88435500 -3.07454000 2.27376300

H -6.03107600 0.32871700 2.91705500

H -5.55526300 -1.23682700 3.56602100

H -4.31961000 -0.12852000 2.96594200

H -7.20210200 -2.27067900 2.10115100

H -7.62933700 -0.80541400 1.21691700

H -7.07750300 -2.23884700 0.34445700

C -0.42197100 5.03598000 0.06300200

C -1.40254900 5.09171700 -1.12268500

C -1.20799700 5.14504100 1.38309500

C 0.51988000 6.24282200 -0.03842400

H -0.87014100 5.01315800 -2.07545900

H -2.14164000 4.28786700 -1.08065500

H -1.94546900 6.04195100 -1.11357200

H -0.53332000 5.11261100 2.24402300

H -1.75689900 6.09153900 1.41618400

H -1.93256100 4.33377000 1.49078700

H -0.07210900 7.16226900 -0.03676900

H 1.21160500 6.29653600 0.80739400

H 1.10536600 6.22782800 -0.96277400

C -0.55874100 -4.97732000 0.06284100

C -1.36751000 -5.05917200 1.37090400

C -1.52259000 -5.01124900 -1.13726200

C 0.35128700 -6.20970100 -0.01941700

H -0.70574500 -5.03849500 2.24218200

H -2.07361200 -4.22998000 1.46240600

H -1.93986300 -5.99179500 1.40038200

H -0.97408400 -4.95445300 -2.08241500

H -2.09474200 -5.94422400 -1.13102100

H -2.23627200 -4.18406300 -1.11044800

H -0.26529500 -7.11283900 -0.01821500

H 0.94732700 -6.21752800 -0.93705700

H 1.03173400 -6.27548800 0.83470200

C 8.25226000 -0.05171300 -0.05071200

C 8.73530100 0.68662800 -1.31312700

C 8.76636400 0.68577300 1.19987500

C 8.84832700 -1.46443200 -0.05865800

H 8.38811900 0.18081100 -2.21927800

H 8.37679000 1.71899200 -1.34354400

H 9.82928900 0.71285200 -1.33595800

H 8.44176300 0.17929700 2.11399400

H 9.86058500 0.71215800 1.19564900

H 8.40858400 1.71806700 1.23986900

H 9.93977900 -1.39696300 -0.07229500

H 8.56325800 -2.03264400 0.83186400

H 8.54103200 -2.03198500 -0.94218900

**5** cation (CAM-B3LYP/6-31g(d,p)):

C -3.04942300 0.04388900 0.03278600

C -1.82874000 0.03924500 0.05666600

C -0.42298600 0.02390500 0.06360100

C 0.30873700 1.25549000 0.05966200

C 0.27791100 -1.22563800 0.06259800

C -0.35056600 2.49602800 0.07216800

C 1.72925300 1.22511500 0.04004900

C 1.69869700 -1.22945200 0.04274000

C -0.41149200 -2.45009300 0.07713700

C 0.33318600 3.70611100 0.06597300

H -1.43306300 2.48921900 0.08587000

C 2.42329600 2.45042100 0.02962400

C 2.42858300 -0.01106200 0.02933900

C 2.36352500 -2.47053900 0.03462800

H -1.49391300 -2.41784000 0.09416000

C 0.24410600 -3.67588600 0.07060500

C 1.73494100 3.65127100 0.04227900

C 1.64673800 -3.65459000 0.04788900

H 2.32915400 4.55551100 0.03282000

H 2.21893600 -4.57291700 0.03983300

C 3.86785700 -0.02879100 0.00483000

C 4.60584300 1.17001400 -0.00963400

C 3.90940800 2.47828900 0.00289600

C 5.99026400 1.13927600 -0.03583000

H 6.50524500 2.09287500 -0.04616200

C 6.70600300 -0.06533400 -0.04904700

C 5.96542000 -1.24826600 -0.03227500

C 4.57579200 -1.24178300 -0.00610000

C 3.84837900 -2.53286200 0.00963300

H 6.45182500 -2.21469700 -0.03969600

O 4.43694200 -3.60277600 0.00209300

O 4.52259000 3.53398200 -0.00831700

Si -4.92577900 0.01691500 -0.04630100

C -5.46579000 1.85871100 0.22001100

C -4.67424700 2.50340200 1.37447700

C -5.18677200 2.70943600 -1.03254700

C -6.96837000 1.96541500 0.54158000

H -4.86464600 2.03502500 2.33950600

H -3.59584000 2.47223900 1.19670200

H -4.96153400 3.55785600 1.46165400

H -5.80429300 2.42039900 -1.88487000

H -5.41552900 3.75844400 -0.81087600

H -4.13833500 2.66405700 -1.34252800

H -7.24368600 3.02172400 0.64467900

H -7.59708600 1.54314000 -0.24648300

H -7.23063800 1.47259100 1.48041700

C -5.29776000 -0.66715900 -1.82532900

C -4.37620400 -0.01465200 -2.87484700

C -5.04569600 -2.18303900 -1.91230400

C -6.76163000 -0.40417000 -2.22527200

H -4.50487600 1.06481600 -2.94908100

H -3.32118500 -0.21508400 -2.66898600

H -4.60360600 -0.43754500 -3.86053900

H -5.72268200 -2.76465300 -1.28432500

H -5.20414100 -2.51121000 -2.94626400

H -4.01806500 -2.44557300 -1.64457100

H -6.95109500 -0.84055000 -3.21318200

H -7.47463400 -0.85544100 -1.53034700

H -6.99068300 0.66135400 -2.29640000

C -5.45667400 -1.17645600 1.38793300

C -4.54007000 -2.41385600 1.44409800

C -5.34465900 -0.49502600 2.76383900

C -6.91249200 -1.64604700 1.20801600

H -4.54154000 -2.99656800 0.52276200

H -3.50701800 -2.13363200 1.66870100

H -4.88044300 -3.07583000 2.24911700

H -6.05059500 0.32821900 2.88663500

H -5.57088500 -1.23080400 3.54450500

H -4.33697700 -0.11601500 2.95794900

H -7.19788800 -2.27652000 2.05847400

H -7.61935500 -0.81283700 1.17215000

H -7.05252600 -2.24343800 0.30456000

C -0.43820600 5.02719900 0.09640100

C -1.41586600 5.07635300 -1.09167800

C -1.22567300 5.11020300 1.41735100

C 0.49551700 6.23956600 0.00704800

H -0.88323400 5.00577200 -2.04390400

H -2.15365600 4.27124700 -1.05316100

H -1.96244900 6.02303300 -1.07763900

H -0.55303100 5.07833500 2.27871900

H -1.78315200 6.05002500 1.45578800

H -1.94468900 4.29322900 1.51712600

H -0.10209100 7.15398700 0.01704600

H 1.18498200 6.28962900 0.85427600

H 1.08017400 6.23721000 -0.91722200

C -0.55780400 -4.97879400 0.09465000

C -1.37609300 -5.03559500 1.39779800

C -1.50942400 -5.01172600 -1.11490000

C 0.34883400 -6.21313400 0.03287100

H -0.72239600 -5.01191500 2.27396800

H -2.07957200 -4.20307000 1.47530800

H -1.95405900 -5.96316000 1.42999700

H -0.95360200 -4.96679600 -2.05536100

H -2.08547400 -5.94085200 -1.10433800

H -2.22132900 -4.18283300 -1.10072200

H -0.26996500 -7.11334500 0.04149600

H 0.94863000 -6.23414800 -0.88135300

H 1.02273700 -6.27002400 0.89213300

C 8.23402400 -0.04321000 -0.08108800

C 8.69812800 0.69801900 -1.34866200

C 8.75125800 0.69739100 1.16607500

C 8.83052600 -1.45463400 -0.09453700

H 8.34628500 0.19094800 -2.25130400

H 8.33735700 1.72911800 -1.37584900

H 9.79077500 0.72742300 -1.37951700

H 8.43610700 0.19112300 2.08262500

H 9.84431300 0.72532600 1.15190000

H 8.39340900 1.72901500 1.20768300

H 9.92066900 -1.38578600 -0.11887800

H 8.55588200 -2.02221100 0.79895300

H 8.51609600 -2.02178000 -0.97514500

**5** anion:

C -2.23320000 0.06269800 0.04818600

C -1.01204300 0.06417100 0.04432600

C 0.39245300 0.04799600 0.03304600

C 1.12450000 1.27695500 0.02591800

C 1.08899600 -1.20188800 0.02478200

C 0.45692000 2.51715500 0.02581900

C 2.54696100 1.24960500 0.01779700

C 2.51167300 -1.21531500 0.01739200

C 0.38604300 -2.42264800 0.02321800

C 1.16443000 3.70263500 0.01971300

H -0.62594200 2.53199200 0.02813900

C 3.24891400 2.47478900 0.01183000

C 3.24631400 0.00689700 0.01382100

C 3.17853600 -2.46001700 0.01160300

H -0.69699900 -2.40725500 0.02465400

C 1.05984900 -3.62763100 0.01717100

C 2.56090000 3.67908100 0.01330000

H 0.63754100 4.64947700 0.01938600

C 2.45640900 -3.64416300 0.01214500

H 0.50607100 -4.55902600 0.01583500

H 3.13199800 4.60041000 0.00841100

H 3.00057600 -4.58166900 0.00748200

C 4.68982500 -0.01362100 0.00424000

C 5.42701900 1.18846400 -0.00109400

C 4.73263100 2.49565200 0.00278400

C 6.81740600 1.15971600 -0.01044000

H 7.35524000 2.10066100 -0.01437700

C 7.49732700 -0.05362600 -0.01473100

C 6.78309200 -1.24708600 -0.00972800

C 5.39243800 -1.23619800 -0.00044900

C 4.66109500 -2.52310000 0.00381100

H 8.58115800 -0.06902600 -0.02202800

H 7.29383600 -2.20300500 -0.01311600

O 5.24864300 -3.59596600 0.00062300

O 5.35032800 3.55143800 -0.00153800

Si -4.11241100 0.00943800 0.00202300

C -4.67300300 1.84183600 0.29793200

C -3.86994100 2.48733900 1.44565800

C -4.43190200 2.71371900 -0.94902300

C -6.17182500 1.92037700 0.64859200

H -4.02153000 1.99610100 2.40647500

H -2.79576200 2.49596500 1.23996300

H -4.18864800 3.53070900 1.55966200

H -5.05411000 2.42035400 -1.79665200

H -4.68561900 3.75344200 -0.70867700

H -3.38630400 2.70126800 -1.27192700

H -6.45924400 2.97203100 0.77003900

H -6.80903300 1.49913000 -0.13362600

H -6.40976700 1.41228100 1.58587700

C -4.49148000 -0.66403300 -1.78153100

C -3.59981800 0.02111200 -2.83749100

C -4.20605400 -2.17367000 -1.88902100

C -5.96685500 -0.42861200 -2.15904800

H -3.74516300 1.09986200 -2.89160700

H -2.53765600 -0.16838300 -2.65731600

H -3.84192600 -0.38976200 -3.82538700

H -4.86466300 -2.77745800 -1.26199700

H -4.36983500 -2.49080800 -2.92624600

H -3.16974800 -2.41795400 -1.63608900

H -6.15671400 -0.85529000 -3.15170200

H -6.66100500 -0.90695400 -1.46286300

H -6.22138500 0.63240200 -2.21178500

C -4.59792300 -1.21092200 1.43269600

C -3.66441200 -2.43787800 1.46046800

C -4.47438500 -0.54490900 2.81620100

C -6.05039500 -1.70031900 1.26992600

H -3.67103900 -3.01006200 0.53264700

H -2.63106300 -2.15156700 1.67650200

H -3.98910700 -3.11267900 2.26196500

H -5.18802200 0.26832000 2.96078600

H -4.68197500 -1.29521300 3.58897100

H -3.46798300 -0.15773500 3.00237300

H -6.30973300 -2.34618600 2.11784200

H -6.77159600 -0.87849900 1.25704700

H -6.19581700 -2.28914500 0.36144100

**6**:

C -2.64939000 -0.11414800 0.00084700

C -1.91627400 -1.31226900 0.00041600

C -2.02981300 1.14634700 0.00012900

C -2.52638900 -2.59921900 0.00032300

C -0.48808800 -1.22945800 -0.00014700

C -0.60023500 1.19490300 -0.00095800

C -2.75543400 2.37189500 0.00019400

C -1.80273700 -3.76685300 -0.00029000

H -3.60771600 -2.64096600 0.00063400

C 0.25631900 -2.44690700 -0.00021200

C 0.15570800 0.01521300 -0.00077000

C 0.02983700 2.47491000 -0.00230400

H -3.83610300 2.31421400 0.00213300

C -2.14148100 3.60088000 -0.00195100

C -0.38854300 -3.66009600 -0.00034200

C -0.72345200 3.62391800 -0.00308100

H 0.23252700 -4.54649900 -0.00054600

H -0.18557600 4.56311100 -0.00449800

C 1.60650600 0.08315400 -0.00100400

C 2.38529600 -1.08833400 -0.00030600

C 1.73794900 -2.42014500 -0.00014800

C 3.77425600 -1.00765300 0.00027800

H 4.31975700 -1.94471700 0.00094100

C 4.45185000 0.21536400 0.00015800

C 3.66951700 1.36806300 -0.00101700

C 2.27389200 1.31745500 -0.00161300

C 1.50781200 2.58377900 -0.00285600

H 4.12092000 2.35183100 -0.00140700

O 2.07290700 3.67698700 -0.00410200

O 2.40116900 -3.45631400 -0.00018400

C -2.52237200 -5.12288100 -0.00059800

C -3.40477700 -5.23202400 1.25691400

C -3.40720900 -5.23045600 -1.25653100

C -1.53867300 -6.30002500 -0.00237000

H -2.80143000 -5.15809100 2.16678500

H -4.16444000 -4.44655300 1.28997700

H -3.92133700 -6.19683000 1.26866600

H -2.80564800 -5.15518800 -2.16747600

H -3.92357400 -6.19535700 -1.26855900

H -4.16718800 -4.44519700 -1.28714300

H -2.10028600 -7.23825100 -0.00307900

H -0.89913100 -6.29813400 -0.89014400

H -0.89820800 -6.30010500 0.88473300

C -2.98167500 4.88564000 0.00035700

C -3.89014400 4.90480200 -1.24305600

C -3.85230400 4.92122200 1.27029200

C -2.10944700 6.14740000 -0.02166300

H -3.29697700 4.87913800 -2.16219900

H -4.57625900 4.05392000 -1.25939900

H -4.49225400 5.81868100 -1.25212600

H -3.23158300 4.90869800 2.17135100

H -4.45540600 5.83440800 1.28531800

H -4.53542700 4.06904600 1.31828000

H -2.75403200 7.03068000 -0.02318000

H -1.46231000 6.21372600 0.85801100

H -1.48180900 6.19571300 -0.91660400

C 5.98385800 0.24343200 0.00135200

C 6.50461800 -0.47772600 1.25871000

C 6.50666200 -0.47869700 -1.25459100

C 6.53443200 1.67451600 0.00128600

H 6.15197700 0.01784400 2.16840500

H 6.17969000 -1.52101900 1.29417800

H 7.59905700 -0.46906500 1.26892800

H 6.15554300 0.01619600 -2.16524100

H 7.60111800 -0.47009900 -1.26300600

H 6.18173200 -1.52200000 -1.28978500

H 7.62755000 1.64203300 0.00229100

H 6.22107300 2.23227300 -0.88633600

H 6.21945600 2.23294800 0.88791300

Cl -4.39451500 -0.19457500 0.00198700

**6** cation:

C -2.65155400 -0.11715700 0.00577400

C -1.90422400 -1.32577000 0.00326000

C -2.02182200 1.15648700 0.00215500

C -2.51910000 -2.59234000 0.00319600

C -0.48103500 -1.24392000 -0.00007900

C -0.59750100 1.20904000 -0.00273900

C -2.75326100 2.35963400 0.00219300

C -1.79141200 -3.77660300 -0.00096100

H -3.59978600 -2.63586600 0.00578900

C 0.25360100 -2.44431700 -0.00168600

C 0.17572600 0.01655600 -0.00244900

C 0.02129700 2.47275200 -0.00783900

H -3.83328800 2.30099200 0.00834700

C -2.13989100 3.60696200 -0.00587200

C -0.39260800 -3.67133100 -0.00257900

C -0.73728800 3.63368200 -0.01041700

H 0.23178000 -4.55521300 -0.00472700

H -0.19869800 4.57236600 -0.01574600

C 1.61292000 0.08475000 -0.00396100

C 2.39383000 -1.08822400 -0.00206400

C 1.74117400 -2.41844700 -0.00244200

C 3.77732300 -1.00905100 0.00020200

H 4.32778500 -1.94254000 0.00205300

C 4.45162500 0.22060600 0.00063700

C 3.66922200 1.37782400 -0.00360800

C 2.27937000 1.32260600 -0.00588800

C 1.50466200 2.58551800 -0.01017700

H 4.12408200 2.35963100 -0.00474800

O 2.05376500 3.67820500 -0.01511000

O 2.38972400 -3.45495600 -0.00297400

C -2.52062600 -5.12189000 -0.00333300

C -3.39888700 -5.21940600 1.25893300

C -3.41035700 -5.20899600 -1.25835200

C -1.54523800 -6.30472400 -0.01279900

H -2.79258500 -5.15330800 2.16700700

H -4.15531100 -4.43098300 1.29494200

H -3.92122300 -6.18022300 1.26823500

H -2.81247500 -5.13378300 -2.17129600

H -3.93150900 -6.17039200 -1.27163800

H -4.16834100 -4.42152300 -1.28045000

H -2.11505800 -7.23722000 -0.01576900

H -0.90969400 -6.30515000 -0.90330400

H -0.90380700 -6.31465000 0.87340100

C -2.99137800 4.87840900 -0.00065500

C -3.93278700 4.86369500 -1.22009500

C -3.82462200 4.91346400 1.29522300

C -2.13155500 6.14622000 -0.06429800

H -3.36597400 4.83111300 -2.15510400

H -4.61519800 4.01001200 -1.20470900

H -4.54171600 5.77207100 -1.22319700

H -3.17860900 4.92507700 2.17790400

H -4.43925000 5.81795400 1.31198200

H -4.49563700 4.05397800 1.37464100

H -2.78550700 7.02176500 -0.06900800

H -1.46908600 6.23686300 0.80144900

H -1.52444300 6.18383100 -0.97371900

C 5.97988800 0.25110600 0.00580900

C 6.49169400 -0.47321800 1.26620600

C 6.50053300 -0.47316900 -1.25094200

C 6.52892700 1.68241500 0.00801700

H 6.13591000 0.02089000 2.17513900

H 6.17125800 -1.51778000 1.29836400

H 7.58542500 -0.46079100 1.27853800

H 6.15124700 0.02101200 -2.16234900

H 7.59433500 -0.46095100 -1.25552200

H 6.18011700 -1.51766900 -1.28534600

H 7.62141100 1.64847300 0.01236000

H 6.22038000 2.24022700 -0.88110400

H 6.21325100 2.24002100 0.89478400

Cl -4.36279900 -0.19783600 0.01219100

**6** anion:

C -2.63656000 -0.12475800 -0.00002900

C -1.90461700 -1.33786800 -0.00000300

C -2.03020200 1.15534000 -0.00002800

C -2.50160800 -2.61357700 0.00000400

C -0.46958600 -1.25028200 0.00002000

C -0.59325900 1.21134300 -0.00001900

C -2.75149200 2.36513500 -0.00004300

C -1.76121800 -3.79891000 0.00003200

H -3.58293700 -2.66378200 -0.00003000

C 0.27733500 -2.45264200 0.00005000

C 0.16601500 0.01601800 0.00000600

C 0.03065000 2.48197600 -0.00003700

H -3.83239900 2.30766400 -0.00003100

C -2.13228900 3.61850900 -0.00005000

C -0.37289100 -3.69665500 0.00004100

C -0.74099600 3.65506400 -0.00003800

H 0.25743500 -4.57569700 0.00007500

H -0.19990700 4.59163900 -0.00005000

C 1.58415100 0.08877800 0.00000800

C 2.37564400 -1.08974600 0.00004300

C 1.74241700 -2.41831400 0.00013100

C 3.76401800 -0.99270400 0.00002600

H 4.31641000 -1.92637400 0.00006400

C 4.43643600 0.23442100 -0.00000500

C 3.64895700 1.38419300 -0.00002600

C 2.25228500 1.33785600 -0.00002800

C 1.49103300 2.59457800 -0.00008400

H 4.09689800 2.36998600 -0.00005500

O 2.06480000 3.70403900 0.00001800

O 2.42434800 -3.46281700 -0.00014500

C -2.49531100 -5.14704500 0.00001400

C -3.38127100 -5.25275600 1.25546400

C -3.38186900 -5.25236700 -1.25504300

C -1.52546600 -6.33529100 -0.00039500

H -2.77659100 -5.18847600 2.16547400

H -4.12980700 -4.45683600 1.29237400

H -3.91112000 -6.21124900 1.26537900

H -2.77762000 -5.18784400 -2.16532300

H -3.91175900 -6.21083800 -1.26498300

H -4.13039100 -4.45640300 -1.29136200

H -2.09473800 -7.26976600 -0.00036700

H -0.88413300 -6.33683000 -0.88676700

H -0.88366500 -6.33705400 0.88563800

C -2.99672400 4.88695200 0.00002500

C -3.88907000 4.90416100 -1.25524900

C -3.88924600 4.90379800 1.25518700

C -2.14986700 6.16577800 0.00025500

H -3.28110800 4.90167200 -2.16535400

H -4.55397500 4.03714800 -1.29288700

H -4.51237400 5.80471300 -1.26431700

H -3.28140600 4.90100000 2.16537200

H -4.51253600 5.80435800 1.26446000

H -4.55416000 4.03677200 1.29243900

H -2.80924000 7.03903900 0.00023100

H -1.51172900 6.23111300 0.88652600

H -1.51146800 6.23124800 -0.88582000

C 5.96975900 0.26790100 0.00000200

C 6.49954800 -0.45028400 1.25520900

C 6.49955900 -0.45020200 -1.25525000

C 6.51731300 1.70037300 0.00005000

H 6.14767100 0.04515000 2.16545500

H 6.17485000 -1.49362200 1.29265500

H 7.59464800 -0.44044800 1.26336700

H 6.14767200 0.04528000 -2.16546500

H 7.59465900 -0.44034700 -1.26340800

H 6.17487900 -1.49354300 -1.29275800

H 7.61110200 1.67225300 0.00005300

H 6.19971300 2.25773200 -0.88635200

H 6.19970800 2.25767500 0.88648500

Cl -4.39383700 -0.21339600 -0.00006700

**8a**:

C -2.77121500 -0.40745500 -0.00002000

C -1.90576700 -1.52333400 0.00000400

C -2.26635500 0.90902700 0.00002200

C -2.37996400 -2.86189900 -0.00000500

C -0.49528600 -1.29039800 0.00003600

C -0.85597700 1.10723600 0.00009400

C -3.13102000 2.03314700 -0.00002500

C -1.54147100 -3.95393200 -0.00000700

H -3.44963700 -3.04888400 -0.00000500

C 0.36967000 -2.42402300 0.00001600

C 0.02019500 0.01274700 0.00008500

C -0.37090000 2.44636400 0.00016100

H -4.19717200 1.84143900 -0.00009500

C -2.65814000 3.32750700 0.00000300

C -0.14911400 -3.69892200 -0.00000600

C -1.25398700 3.50506400 0.00011100

H 0.56073500 -4.51612200 -0.00002000

H -0.82423100 4.49877900 0.00016400

C 1.44727900 0.23168300 0.00008500

C 2.34632600 -0.85359100 0.00001800

C 1.84113800 -2.24369300 0.00000900

C 3.71920200 -0.62481400 -0.00004000

H 4.36216100 -1.49814800 -0.00008900

C 4.26350900 0.66315100 -0.00003300

C 3.36388300 1.72724200 0.00008000

C 1.98044500 1.53294800 0.00014300

C 1.08555500 2.71147400 0.00029100

H 3.71109300 2.75252300 0.00011700

O 1.53263100 3.86074800 0.00050400

O 2.60698700 -3.20914800 -0.00001700

C -2.11721300 -5.37726500 0.00000600

C -2.98355200 -5.57928500 1.25726800

C -2.98366900 -5.57929600 -1.25716500

C -1.01556000 -6.44470100 -0.00004400

H -2.39069000 -5.44434700 2.16694900

H -3.82143200 -4.87773200 1.29344800

H -3.39777700 -6.59223800 1.26803200

H -2.39087100 -5.44447300 -2.16690600

H -3.39797700 -6.59221600 -1.26782600

H -3.82150000 -4.87768500 -1.29333700

H -1.47547000 -7.43674200 -0.00008100

H -0.37925500 -6.37612700 -0.88736700

H -0.37924500 -6.37619900 0.88727800

C -3.63767300 4.50949300 -0.00011100

C -4.52530400 4.43723000 -1.25654300

C -4.52578900 4.43711500 1.25597000

C -2.91326900 5.86153500 0.00009800

H -3.92030100 4.48932200 -2.16688900

H -5.10737700 3.51232800 -1.28991000

H -5.22928200 5.27534500 -1.26733500

H -3.92113100 4.48898100 2.16655800

H -5.22967400 5.27530900 1.26663800

H -5.10799300 3.51228200 1.28895900

H -3.65283100 6.66711900 0.00001900

H -2.28608200 5.98963600 0.88743700

H -2.28577500 5.98974800 -0.88700600

C 5.78453900 0.85247300 -0.00012100

C 6.38077200 0.19033500 1.25609400

C 6.38057900 0.19073900 -1.25664300

C 6.18205700 2.33341500 0.00008600

H 5.97915900 0.64618300 2.16631300

H 6.16675000 -0.88123600 1.29200000

H 7.46841900 0.31360900 1.26502800

H 5.97880500 0.64686400 -2.16665200

H 7.46822100 0.31404000 -1.26571800

H 6.16657500 -0.88082400 -1.29284900

H 7.27266700 2.41578700 0.00000500

H 5.81085300 2.85569500 -0.88683700

H 5.81100700 2.85540800 0.88724100

O -4.11387000 -0.52585200 -0.00006300

H -4.40026400 -1.44732200 -0.00016200

**8a** cation:

C -2.76052900 -0.46093800 -0.00014400

C -1.86198300 -1.57671700 -0.00013800

C -2.27650600 0.88311300 -0.00008700

C -2.31948400 -2.90273900 -0.00011200

C -0.46447700 -1.31715400 -0.00010400

C -0.87726100 1.10878700 -0.00020000

C -3.16554500 1.96695200 0.00004800

C -1.45379800 -3.99246000 0.00002000

H -3.38408500 -3.11044000 -0.00025000

C 0.41252300 -2.41727500 -0.00003500

C 0.03561900 0.01760600 -0.00017400

C -0.42412700 2.44013700 -0.00030400

H -4.22697700 1.75354100 0.00021500

C -2.71809900 3.28602300 -0.00004300

C -0.08032300 -3.71449400 0.00004400

C -1.33131000 3.49241800 -0.00027500

H 0.64731200 -4.51560400 0.00012300

H -0.91897800 4.49324800 -0.00042700

C 1.44839300 0.26150900 -0.00015800

C 2.36846800 -0.80764600 -0.00004700

C 1.88533400 -2.20734900 -0.00007800

C 3.73197500 -0.55628900 0.00007200

H 4.39398100 -1.41443500 0.00017100

C 4.24851200 0.74725000 0.00005200

C 3.32914700 1.79893600 -0.00013200

C 1.95560700 1.57486400 -0.00023100

C 1.03198100 2.73405100 -0.00037700

H 3.65987600 2.82923400 -0.00019500

O 1.44538700 3.88703900 -0.00054600

O 2.65754100 -3.15727300 -0.00014900

C -2.01110500 -5.41850600 0.00006400

C -2.87468800 -5.62311400 1.25959900

C -2.87456600 -5.62327000 -1.25950300

C -0.89411500 -6.46881500 0.00018600

H -2.28292600 -5.48258700 2.16875100

H -3.72294300 -4.93420700 1.29601300

H -3.27580600 -6.64049200 1.26889100

H -2.28266600 -5.48307900 -2.16861700

H -3.27585600 -6.64058800 -1.26858700

H -3.72271500 -4.93423500 -1.29624800

H -1.34063100 -7.46623600 0.00023800

H -0.25997800 -6.39219300 -0.88780900

H -0.26000400 -6.39205300 0.88818100

C -3.72908000 4.43534200 0.00019300

C -4.61253400 4.32856300 -1.25751000

C -4.61087100 4.32931800 1.25913000

C -3.04311900 5.80632800 -0.00070400

H -4.01046900 4.39851100 -2.16823800

H -5.17043800 3.38888200 -1.28867700

H -5.33930400 5.14591200 -1.26687200

H -4.00766300 4.40052000 2.16900100

H -5.33810300 5.14625700 1.26860000

H -5.16812900 3.38930400 1.29194200

H -3.80641300 6.58854000 -0.00045500

H -2.42109200 5.95388900 0.88697100

H -2.42222600 5.95325200 -0.88928700

C 5.76210400 0.96608200 0.00019200

C 6.36455700 0.31209500 1.25868700

C 6.36487500 0.31152200 -1.25785200

C 6.13168800 2.45397200 -0.00010200

H 5.95319400 0.75910300 2.16858000

H 6.17541100 -0.76398800 1.29259100

H 7.44848000 0.45962500 1.26757700

H 5.95378400 0.75815700 -2.16805300

H 7.44880800 0.45899800 -1.26650500

H 6.17568500 -0.76456600 -1.29134300

H 7.22027600 2.55366700 0.00001900

H 5.75378600 2.96926400 -0.88815400

H 5.75354900 2.96967500 0.88760900

O -4.06527900 -0.59580600 -0.00001200

H -4.37151400 -1.51515300 0.00002700

**8a** anion:

C -2.72136300 -0.58349500 0.03583400

C -1.79050700 -1.65248100 0.01660000

C -2.32304700 0.77406000 0.01519200

C -2.19380500 -2.99892100 0.01702900

C -0.39310000 -1.34032200 0.00116300

C -0.91377400 1.06729800 0.01989400

C -3.23663600 1.84705900 -0.00917200

C -1.27661900 -4.05758900 -0.00289400

H -3.25791100 -3.20218700 0.03360300

C 0.53126700 -2.40824800 -0.01509300

C 0.03019300 0.01287600 0.01070200

C -0.50682200 2.42140900 0.02613600

H -4.30168200 1.63941500 -0.05341000

C -2.83150900 3.18982100 -0.00200000

C 0.07846400 -3.73997200 -0.01902400

C -1.46782900 3.45399200 0.02057100

H 0.83891000 -4.50935600 -0.03315600

H -1.08992100 4.46741900 0.02694500

C 1.41397500 0.31264200 0.00741300

C 2.38456000 -0.72571000 -0.00960800

C 1.97177300 -2.14000600 -0.02429300

C 3.73817100 -0.40564700 -0.01250700

H 4.43523800 -1.23707400 -0.02626700

C 4.20305900 0.91551800 0.00124900

C 3.24164000 1.92366500 0.01736400

C 1.86904800 1.65516900 0.01991500

C 0.91260000 2.76976100 0.03312500

H 3.52820700 2.96780400 0.02753200

O 1.29837500 3.96151200 0.04700900

O 2.81175100 -3.06265400 -0.04195700

C -1.79209100 -5.50338600 -0.00513900

C -2.63675900 -5.75288400 1.25839100

C -2.66671200 -5.73757800 -1.25116600

C -0.65039000 -6.52726800 -0.02508400

H -2.03854700 -5.60263200 2.16258200

H -3.49783700 -5.08122500 1.30937100

H -3.01337000 -6.78142200 1.26602100

H -2.09000900 -5.57652100 -2.16738400

H -3.04382900 -6.76591200 -1.26230000

H -3.52837000 -5.06507900 -1.27371800

H -1.06777400 -7.53886700 -0.02612300

H -0.02710000 -6.42381700 -0.91824500

H -0.00553900 -6.43447100 0.85384400

C -3.89267200 4.29887300 -0.02972100

C -4.73929900 4.17310500 -1.31046900

C -4.81231300 4.16928400 1.19911800

C -3.26829600 5.69967500 -0.00887800

H -4.11227700 4.26903800 -2.20233000

H -5.25553700 3.21079000 -1.36509500

H -5.49906600 4.96141400 -1.34143400

H -4.23839000 4.26199800 2.12639900

H -5.57227100 4.95789200 1.18850500

H -5.33151300 3.20730800 1.22030000

H -4.06242000 6.45232100 -0.02719400

H -2.67274600 5.86586000 0.89364900

H -2.62665400 5.87202100 -0.87793600

C 5.71117100 1.19548500 -0.00252000

C 6.35664200 0.55584400 1.24098700

C 6.34424000 0.58890900 -1.26877100

C 6.02153700 2.69725400 0.01575900

H 5.93396900 0.97596900 2.15896700

H 6.20433500 -0.52660300 1.26508100

H 7.43596000 0.74195500 1.24629000

H 5.91303400 1.03345800 -2.17114800

H 7.42357400 0.77472200 -1.27953300

H 6.19102200 -0.49246700 -1.32010700

H 7.10569200 2.84525100 0.01272500

H 5.61427200 3.20796100 -0.86206000

H 5.62237100 3.18470800 0.91037500

O -4.04564200 -0.94362300 0.05167300

H -4.59849200 -0.19070700 0.29085400

**3a**:

C 5.29728800 0.00005900 0.00036900

C 4.09033500 0.00059500 -0.00009100

C 2.66108100 0.00039200 -0.00007100

C 1.95814500 1.22508700 -0.00005600

C 1.95847300 -1.22449900 -0.00004700

C 2.63970100 2.47656200 -0.00003900

C 0.52911600 1.21690800 -0.00004800

C 0.52944300 -1.21671000 -0.00004000

C 2.64036800 -2.47578600 -0.00001600

C 1.95054400 3.65726700 -0.00001200

H 3.72346900 2.47667500 -0.00004500

C -0.16354300 2.46903600 -0.00003900

C -0.17324700 0.00000600 -0.00004000

C -0.16287700 -2.46902100 -0.00003000

H 3.72413700 -2.47561000 -0.00001700

C 1.95153000 -3.65668000 0.00001500

C 0.53847600 3.65012500 -0.00001600

H 2.48363200 4.60131600 0.00001200

C 0.53946400 -3.64992200 -0.00000300

H 2.48487500 -4.60058300 0.00005100

H -0.01609100 4.58203500 -0.00000100

H -0.01485100 -4.58198100 0.00001100

C -1.62906100 -0.00019200 0.00000500

C -2.34946400 1.20965800 0.00003800

C -1.64252700 2.50954900 -0.00005700

C -3.74397200 1.19836100 0.00013900

H -4.26406000 2.14969200 0.00016300

C -4.44498700 -0.00057100 0.00020500

C -3.74364900 -1.19931400 0.00013700

C -2.34913800 -1.21023600 0.00003700

C -1.64185100 -2.50993500 -0.00005100

H -5.52925500 -0.00071700 0.00030700

H -4.26348200 -2.15078500 0.00015800

O -2.25778000 -3.57435100 -0.00020500

O -2.25874200 3.57379900 -0.00014000

H 6.36590500 -0.00007100 0.00039400

**3a** cation:

C 5.27915000 -0.00000800 0.00002700

C 4.07075800 -0.00002300 0.00001500

C 2.65919300 -0.00001900 0.00000700

C 1.95075500 1.23832400 0.00000400

C 1.95073900 -1.23835200 0.00000500

C 2.63852400 2.46858900 0.00000900

C 0.52725500 1.23235600 -0.00000800

C 0.52723800 -1.23236500 -0.00000300

C 2.63849200 -2.46862700 0.00000600

C 1.94779300 3.66408600 0.00000300

H 3.72185700 2.46804100 0.00001800

C -0.15554400 2.46703400 -0.00001700

C -0.19021400 0.00000000 -0.00000200

C -0.15557600 -2.46703400 -0.00000500

H 3.72182500 -2.46809300 0.00001100

C 1.94774500 -3.66411500 -0.00000200

C 0.55118000 3.66117400 -0.00001200

H 2.48832400 4.60315100 0.00000700

C 0.55113200 -3.66118400 -0.00001000

H 2.48826100 -4.60318800 0.00000000

H -0.00582000 4.59112300 -0.00002200

H -0.00588000 -4.59112500 -0.00001400

C -1.63352600 0.00000900 0.00000100

C -2.35315200 1.21272200 0.00000200

C -1.63938200 2.50978400 -0.00004600

C -3.74357800 1.20395100 0.00003300

H -4.26834900 2.15222500 0.00003300

C -4.44042400 0.00002800 0.00005900

C -3.74359400 -1.20390500 0.00004400

C -2.35316800 -1.21269500 0.00001700

C -1.63941500 -2.50976600 0.00000900

H -5.52437500 0.00003500 0.00008900

H -4.26837800 -2.15217100 0.00005700

O -2.24073600 -3.57439600 -0.00008300

O -2.24068900 3.57442200 -0.00004300

H 6.34964500 -0.00000100 0.00001800

**3a** anion:

C 5.29551100 -0.00000300 -0.00036000

C 4.08325000 0.00002500 -0.00026700

C 2.66380400 0.00001600 -0.00013200

C 1.95655000 1.24348800 -0.00000700

C 1.95656600 -1.24345700 -0.00003300

C 2.62602200 2.48238100 0.00018500

C 0.52236700 1.23494600 -0.00004000

C 0.52238400 -1.23494200 -0.00006800

C 2.62606600 -2.48233000 0.00014800

C 1.92898100 3.68421200 0.00029700

H 3.71045700 2.48782200 0.00026400

C -0.17008300 2.47549500 0.00004300

C -0.18046000 -0.00000300 -0.00012400

C -0.17004500 -2.47550000 -0.00002000

H 3.71050200 -2.48774700 0.00019000

C 1.92904700 -3.68417300 0.00027600

C 0.54024700 3.68270700 0.00020500

H 2.47395600 4.62292900 0.00046100

C 0.54031300 -3.68269600 0.00018100

H 2.47404200 -4.62288000 0.00044100

H -0.02195200 4.60960000 0.00027800

H -0.02185500 -4.60960200 0.00023500

C -1.60205800 -0.00001200 -0.00011800

C -2.33172500 1.22090400 -0.00006000

C -1.63476400 2.51401200 -0.00000400

C -3.72608700 1.19945300 -0.00002500

H -4.24791400 2.15050100 0.00002100

C -4.42925200 -0.00002700 -0.00002300

C -3.72607300 -1.19950100 -0.00007200

C -2.33171100 -1.22093500 -0.00013500

C -1.63473100 -2.51404100 -0.00026600

H -5.51410800 -0.00003200 0.00001700

H -4.24788900 -2.15055400 -0.00008900

O -2.26266300 -3.59068600 0.00022100

O -2.26270900 3.59065000 -0.00007300

H 6.36302500 0.00014100 -0.00048000

**SubPC-Ar**:

B -0.60656200 -0.00001200 -0.65485200

N -2.04494700 -0.01377000 -1.04420600

C -2.77716700 -1.15850300 -1.11220900

C -2.79546200 1.11823500 -1.12492400

C -4.10939300 -0.74427800 -1.52876200

C -4.12083000 0.67803100 -1.53675000

N -0.48676800 1.18151500 0.25986100

C -1.27970800 2.28212400 0.13603800

C 0.23385200 1.16197800 1.41434600

C -0.84842400 3.19496900 1.18559700

C 0.09711700 2.49502900 1.98417700

N -0.46777700 -1.16928900 0.27293500

C 0.25230100 -1.12525600 1.42711300

C -1.24301900 -2.28377500 0.16153200

C 0.13688800 -2.45384400 2.01183300

C -0.79725300 -3.17776000 1.22122500

N 0.67586200 0.02487500 1.96059300

N -2.38498400 2.28460100 -0.61539100

N -2.34807800 -2.31236600 -0.58969000

C -5.27057200 -1.46561800 -1.80270800

C -5.29345700 1.37742200 -1.81879400

C -6.43575600 0.64764200 -2.11406700

C -6.42445600 -0.75772600 -2.10609300

H -5.30756300 2.46181400 -1.81169700

H -7.35702500 1.16892700 -2.35184800

H -5.26712700 -2.54994400 -1.78330800

C -1.25433800 4.48327400 1.53041400

C -0.69139600 5.06729800 2.65604700

C 0.24352800 4.37564600 3.44499000

C 0.63689300 3.08374200 3.12668800

H -1.98623100 5.01311900 0.93063700

H -0.97953000 6.07488200 2.93674800

H 0.66049300 4.86155600 4.32079500

H 1.34785700 2.54599100 3.74431200

C 0.68591900 -3.02096200 3.16085300

C -1.18252500 -4.46845200 1.58052300

C -0.61038600 -5.03068100 2.71259300

C 0.31324500 -4.31533000 3.49366700

H 1.38813500 -2.46495600 3.77229400

H -1.90583200 -5.01664400 0.98684600

H 0.73781000 -4.78465400 4.37484900

O 0.24066600 0.00035900 -1.81733700

C 1.60637700 0.01231800 -1.68474600

C 2.31859500 -1.18559000 -1.63432300

C 2.30081100 1.21600700 -1.64581900

C 3.70421200 -1.16582000 -1.53792100

H 1.77997900 -2.12676200 -1.67788400

C 3.69189000 1.21951700 -1.54889100

H 1.75031300 2.14983100 -1.69834300

C 4.42751400 0.03373400 -1.49018700

H 4.22847900 -2.11559500 -1.50195500

H 4.19647200 2.17779700 -1.52212600

C 5.95768900 0.00275700 -1.38563600

C 6.54188700 -0.73250700 -2.60645400

H 7.63452300 -0.76459600 -2.54163000

H 6.18109000 -1.76257500 -2.67048100

H 6.27076100 -0.22213100 -3.53587500

C 6.37091500 -0.74040000 -0.10143900

H 5.97543000 -0.23611400 0.78572000

H 6.00616500 -1.77099900 -0.09352000

H 7.46225600 -0.77174200 -0.01748000

C 6.56536300 1.41031600 -1.33938200

H 6.33787300 1.98253500 -2.24387200

H 6.21048000 1.97893700 -0.47446600

H 7.65393100 1.33433000 -1.26149100

H -7.33719000 -1.29645100 -2.33783100

H -0.88233800 -6.03953300 3.00466300

**SubPC-Ar** cation:

B -0.56244200 -0.00029800 -0.70204000

N -2.00553700 -0.03641100 -1.09988200

C -2.73792000 -1.18454500 -1.12833900

C -2.78501000 1.07871500 -1.16612700

C -4.08211000 -0.79643000 -1.55491600

C -4.11161400 0.62087900 -1.57878900

N -0.48210400 1.19325600 0.21617200

C -1.31260500 2.26839800 0.09747500

C 0.18621400 1.18770500 1.40359900

C -0.92349300 3.20130000 1.15511500

C 0.01504200 2.52430800 1.97340500

N -0.43335900 -1.15811400 0.25503900

C 0.23424300 -1.08584000 1.44064000

C -1.21775000 -2.27047200 0.17205800

C 0.11944000 -2.40921300 2.05359300

C -0.78938000 -3.15164200 1.25869800

N 0.63904000 0.06898100 1.97277300

N -2.40385800 2.25789100 -0.66995600

N -2.30833900 -2.33005100 -0.59408200

C -5.22078100 -1.53961200 -1.81608700

C -5.28015800 1.30662500 -1.86398400

C -6.41809500 0.55209800 -2.15815300

C -6.38899300 -0.84354500 -2.13460700

H -5.30949100 2.39019600 -1.86397000

H -7.34403700 1.06025000 -2.40341000

H -5.20473500 -2.62285400 -1.77977200

C -1.36747000 4.47246700 1.47784500

C -0.83236100 5.07442800 2.61889900

C 0.09274200 4.40778000 3.42452900

C 0.51874200 3.11269600 3.12108000

H -2.10018300 4.98393600 0.86419800

H -1.14627600 6.07691000 2.88772500

H 0.48304200 4.90287300 4.30671100

H 1.22220300 2.58911500 3.75826900

C 0.64771100 -2.93809000 3.21901700

C -1.17838900 -4.42970200 1.62277600

C -0.61808500 -4.97090400 2.78204900

C 0.27731100 -4.23958400 3.56476100

H 1.32826500 -2.36459900 3.83776800

H -1.88830700 -4.99172100 1.02686800

H 0.68807100 -4.68879900 4.46207000

O 0.28942800 -0.00194900 -1.83727900

C 1.65990000 0.01175000 -1.68584400

C 2.36725400 -1.18687700 -1.62438500

C 2.34809800 1.21716300 -1.64065900

C 3.75159400 -1.16535400 -1.51102100

H 1.83075200 -2.12878300 -1.67739100

C 3.73793700 1.22093500 -1.52651900

H 1.79883300 2.15084500 -1.70653700

C 4.47278000 0.03527400 -1.45766800

H 4.27638500 -2.11429800 -1.46958100

H 4.24222600 2.17903400 -1.49767300

C 6.00187200 0.00573400 -1.34059500

C 6.59457900 -0.72636200 -2.55929100

H 7.68649600 -0.75758200 -2.48633800

H 6.23537300 -1.75664300 -2.62835300

H 6.33011000 -0.21383400 -3.48934600

C 6.40500100 -0.74032200 -0.05491600

H 6.00265700 -0.23842600 0.83057800

H 6.04261900 -1.77179000 -0.05239000

H 7.49548100 -0.77009000 0.03712300

C 6.60714500 1.41395800 -1.28636000

H 6.38809100 1.98746000 -2.19204700

H 6.24414700 1.98068600 -0.42351900

H 7.69480200 1.33870400 -1.19861700

H -7.29269700 -1.39796300 -2.36183100

H -0.88828500 -5.97670700 3.08375800

**SubPC-Ar** anion:

B -0.62251500 -0.00051400 -0.61862000

N -2.05082600 0.04389900 -1.02739900

C -2.80696800 -1.07333300 -1.16067700

C -2.76427300 1.21571700 -1.10741200

C -4.10877500 -0.60215500 -1.56684600

C -4.07657500 0.83575400 -1.51657500

N -0.43416600 1.16448300 0.29588000

C -1.21363700 2.29508300 0.20608800

C 0.25983500 1.08053200 1.47986200

C -0.72133800 3.18130000 1.27191500

C 0.18884000 2.43100200 2.05863900

N -0.52949100 -1.19694100 0.27167800

C 0.20120200 -1.21635800 1.43541300

C -1.31821000 -2.28848900 0.10107200

C 0.02169700 -2.52159100 1.97936900

C -0.92271100 -3.20834900 1.13822200

N 0.64852200 -0.05333300 2.01379300

N -2.30031900 2.37057800 -0.52587100

N -2.39470100 -2.29280400 -0.72829300

C -5.29767200 -1.27317000 -1.88492100

C -5.25015300 1.56750300 -1.78072400

C -6.40335600 0.88302500 -2.10623900

C -6.43092300 -0.53168600 -2.16131300

H -5.24199300 2.65192000 -1.73425700

H -7.31164100 1.43733900 -2.32287500

H -5.32212400 -2.35819000 -1.91327800

C -1.05489300 4.48356100 1.62449800

C -0.45552000 5.03845300 2.75237100

C 0.44370900 4.29748500 3.52900000

C 0.76458900 2.98388900 3.19613200

H -1.76342800 5.05291700 1.03208500

H -0.69126000 6.05882400 3.03713400

H 0.89321200 4.75347400 4.40524900

H 1.44993000 2.40487000 3.80607400

C 0.51609900 -3.15710600 3.13467800

C -1.34670300 -4.50514300 1.46212700

C -0.83135900 -5.11498500 2.59001200

C 0.09526100 -4.43943000 3.42152900

H 1.22094200 -2.64518300 3.78219400

H -2.06700100 -5.01785300 0.83216300

H 0.47793700 -4.94376800 4.30376400

O 0.23975100 -0.01507200 -1.79918100

C 1.59764500 -0.01158600 -1.67045700

C 2.30324100 -1.20598000 -1.50806000

C 2.31126500 1.18086900 -1.75143100

C 3.68971900 -1.19295300 -1.42422200

H 1.75458000 -2.14043300 -1.45036200

C 3.70335700 1.17867300 -1.66807400

H 1.76895400 2.11168500 -1.88393600

C 4.42773500 -0.00382200 -1.50103500

H 4.20357400 -2.14099500 -1.29731100

H 4.21792000 2.12989100 -1.73689800

C 5.95847000 -0.04269000 -1.40507000

C 6.52594400 -0.90492600 -2.54822200

H 7.61881500 -0.94455200 -2.48722800

H 6.15170000 -1.93128300 -2.50544500

H 6.25412100 -0.48797200 -3.52302900

C 6.37520800 -0.65609800 -0.05517100

H 5.99250700 -0.05970000 0.77882500

H 5.99751300 -1.67550700 0.06030700

H 7.46701800 -0.69272700 0.02397200

C 6.58293700 1.35465100 -1.50687600

H 6.35213200 1.83535500 -2.46243500

H 6.24272500 2.01210600 -0.70106800

H 7.67155500 1.27391500 -1.43132100

H -7.35708000 -1.03560200 -2.41905400

H -1.14130800 -6.12244600 2.84915600

# References

[1] G. E. Morse, A. S. Paton, A. Lough, T. P. Bender. *Dalton Trans.* **2010**, *39* (16), 3915–3922.

[2] A. V. Muñoz, H. Gotfredsen, M. Jevric, A. Kadziola, O. Hammerich, M. B. Nielsen. *J. Org. Chem.* **2018**, *83* (4), 2227–2234.

[3] P. Ribar, L. Valenta, T. Šolomek, M. Juríček. *Angew. Chem. Int. Ed.* **2021**, *60* (24), 13521–13528.

[4] P. Ribar, T. Šolomek, L. Le Pleux, D. Häussinger, A. Prescimone, M. Neuburger, M. Juríček. *Synthesis* **2017**, *49* (4), 899–909.

[5] R. C. Clark, J. S. Reid. *Acta Cryst.* **1995**, *A51*, 887–897.

[6] CrysAlisPro(version 1.171.40.68a). Rigaku Oxford Diffraction Ltd: Yarnton, Oxfordshire, England 2019.

[7] O. V. Dolomanov, L. J. Bourhis, R. J. Gildea, J. A. K. Howard, H. Puschmann. *J. Appl. Cryst.* **2009**, *42*, 339–341.

[8] G. M. Sheldrick. *Acta Cryst.* **2015**, *A71*, 3–8.

[9] G. M. Sheldrick. *Acta Cryst.* **2015**, *C71*, 3–8.

[10] A. L. Spek. *Acta Cryst.* **2009**, *D65*, 148–155.

[11] A. L. Spek. *Acta Cryst.* **2015**, *C71*, 9–18.

[12] T. Yanai, D. P. Tew, N. C. Handy. *Chem. Phys. Lett.* **2004**, *393* (1–3), 51–57.

[13] Y. Zhao, D. G. Truhlar. *Theor. Chem. Acc.* **2008**, *120* (1–3), 215–241.

[14] M. M. Francl, W. J. Pietro, W. J. Hehre, J. S. Binkley, M. S. Gordon, D. J. DeFrees, J. A. Pople. *J. Chem. Phys.* **1982**, *77* (7), 3654–3665.

[15] Gaussian 16 (Revision A.03). Gaussian Inc: Wallingford, Oxfordshire, England 2016.

[16] J. E. Bartmess. *J. Phys. Chem.* **1994**, *98* (25), 6420–6424.

[17] N. Ree, C. L. Andersen, M. D. Kilde, O. Hammerich, M. B. Nielsen, K. V. Mikkelsen. *Phys. Chem. Chem. Phys.* **2018**, *20* (11), 7438–7446.
